# Systematic analysis of global trends in otitis media burden across Socio-Demographic Index levels, 1990–2021: findings from the global burden of disease study 2021

**DOI:** 10.3389/fpubh.2026.1694807

**Published:** 2026-03-12

**Authors:** Can Wang, Yuyin Liu, Chao Liao, Lu Zhang, Yuanlun Xie, Li Tian

**Affiliations:** 1 Department of Otolaryngology, Hospital of Chengdu University of Traditional Chinese Medicine, Chengdu, China; 2 School of Clinical Medicine, Chengdu University of Traditional Chinese Medicine, Chengdu, China; 3 Department of Otolaryngology, Leshan Traditional Chinese Medicine Hospital, Leshan, China; 4 School of Electronic Information and Electrical Engineering, Chengdu University, Chengdu, China

**Keywords:** age-period-cohort analysis, Frontier analysis, global burden of disease, otitis media, Socio-Demographic Index

## Abstract

**Background:**

Otitis media (OM) represents a major global health burden, particularly in children. While its association with socioeconomic development is recognized, the complex temporal dynamics across developmental stages remain unclear. This study analyzes the multidimensional relationship between the Socio-Demographic Index (SDI) and OM burden to inform targeted health strategies.

**Methods:**

Using Global Burden of Disease (GBD) Study 2021 data from 204 countries, we assessed OM prevalence, incidence, and years lived with disability (YLDs). We employed Spearman's correlation, Age-Period-Cohort (APC) modeling stratified by SDI, and Frontier analysis to evaluate disease burden patterns and control efficiency.

**Results:**

From 1990–2021, Age-standardized rate (ASR) of prevalence and YLDs decreased by 11.18 and 11.64% respectively, while incidence paradoxically increased by 3.75%. Strong negative correlations existed between SDI and disease burden (ρ = −0.928 for prevalence), except for incidence in High-SDI regions showing positive correlation with development. APC analysis revealed universal early childhood burden peaks that decreased with increasing SDI. Low-SDI regions showed minimal improvement (7.41% prevalence reduction) despite highest burden potential. Recent birth cohorts demonstrated declining prevalence but rising incidence. Frontier analysis identified unexpected efficiency patterns: some low-income countries achieved optimal incidence control while certain high-income nations showed significant gaps.

**Conclusion:**

Global OM epidemiology shows divergent trends: decreasing prevalence alongside rising incidence, with pronounced disparities across SDI levels. High-SDI regions face emerging incidence challenges while Low-SDI regions show the slowest improvement despite highest burden. The 0–5 age group demonstrates concerning upward trends across multiple SDI levels, not just in resource-limited settings. These findings support SDI and age-stratified intervention strategies, prioritizing early childhood programs universally, with resource intensity calibrated to regional burden.

## 1 Introduction

Otitis media (OM) is the second most common pediatric disease after upper respiratory viral infections and a leading cause of preventable hearing loss worldwide, posing a persistent challenge to global public health (1, 2). According to the Global Burden of Disease (GBD) Study 2021, otitis media accounted for 391 million episodes globally in 2021. Approximately 60% of children experience at least one episode of acute otitis media (AOM) before the age of 3 years, and up to 80% experience at least one episode by age 5 or during their lifetime (3–5). Its clinical spectrum is broad, ranging from self-limiting AOM to conditions that can cause permanent hearing impairment, such as chronic suppurative otitis media (CSOM) and otitis media with effusion (OME) (5–7). OM is commonly associated with viral upper respiratory infections and bacterial pathogens including Streptococcus pneumoniae, nontypeable Haemophilus influenzae, and Moraxella catarrhalis; additionally, host susceptibility factors, including anatomical immaturity, genetic predisposition, and environmental exposures such as daycare attendance and secondhand smoke, are also associated with disease occurrence (7–9). The burden is particularly pronounced for children: recurrent episodes not only cause acute symptoms like otalgia and fever but, more critically, the associated conductive hearing loss occurs during a crucial period of language and cognitive development, potentially having profound adverse effects on academic performance, social skills, and future career prospects (10–12). The treatment of otitis media and its complications also imposes a substantial economic burden on healthcare systems; for instance, the estimated treatment cost for otitis media in children under five in the United States alone was as high as $4.8 billion in 2020 (13).

In many high-income countries, interventions like the pneumococcal conjugate vaccine, improved sanitation, and standardized antibiotic therapies have effectively reduced the burden of severe OM (14). However, the global landscape of OM burden remains highly heterogeneous. To develop cost-effective and targeted prevention strategies, it is critical to understand these variations, especially across different Socio-Demographic Index (SDI) levels. Extensive previous research has outlined overall trends and indicated a significant correlation with socioeconomic development (15, 16). However, these foundational studies have primarily focused on macro-level descriptions of burden metrics, without fully exploring the complex temporal dynamics driving these long-term trends and their deep-seated interplay with socioeconomic development.

The GBD study provides an unparalleled platform for systematically assessing the spatiotemporal distribution, long-term trends, and association with social development for specific diseases (17, 18). This study aims to leverage the latest data from GBD 2021 to conduct a comprehensive and in-depth analysis of the burden of otitis media (including prevalence, incidence, and years lived with disability) globally, regionally, and across 204 countries and territories from 1990 to 2021. For the first time, this study systematically introduces the APC model, stratified by the SDI, to deconstruct the long-term trends of the otitis media burden from multiple dimensions. It also utilizes Frontier analysis to assess the potential efficiency of disease control efforts in countries at different developmental stages. Through this research, we expect to reveal the complex epidemiological characteristics of the global burden of otitis media and clarify the multidimensional relationship between socioeconomic development and disease burden, thereby providing crucial evidence-based support for optimizing global health resource allocation and formulating more targeted regional intervention strategies.

## 2 Methods

### 2.1 Data source

The data for this study were derived from the publicly available results of the Global Burden of Disease Study 2021 (GBD 2021). GBD 2021 (https://vizhub.healthdata.org/gbd-results/) is a systematic and comprehensive analysis that evaluates health losses caused by 371 diseases and injuries across 204 countries and territories from 1990 to 2021. The overall disease burden analysis in GBD 2021 utilized 100,983 data sources. These data sources were diverse, including vital registration systems, epidemiological surveillance data, disease registry data, household surveys, literature, and healthcare utilization data. To ensure consistent estimates across all global regions, particularly in areas where original data were scarce, the GBD study employed the Bayesian meta-regression tool DisMod-MR 2.1. This model can integrate different types of data and generate coherent epidemiological metric estimates (19).

We extracted the following OM burden metrics from the GBD 2021 database: prevalence, incidence, and years lived with disability (YLDs). For each metric, we obtained both absolute numbers and age-standardized rates (expressed per 100,000 population). Data were extracted at global, 5 SDI regions, 21 GBD regions, and 204 countries levels. All data were further stratified by age group and sex.

### 2.2 Socio-Demographic Index

The GBD studies conduct annual evaluations of the SDI, a comprehensive indicator of socioeconomic development that affects health outcomes worldwide. The SDI integrates three key components through geometric mean calculations: total fertility rate for populations under 25 years (TFU25), average educational attainment for individuals aged 15 and above, and lag distributed income per capita, producing a composite score from 0 to 1 where higher values indicate greater socioeconomic progress (20). Based on SDI values, countries are classified into five developmental categories: High SDI (>0.81), High-middle SDI (0.70–0.81), Middle SDI (0.61–0.69), Low-middle SDI (0.46–0.60), and Low SDI (<0.46) (18).

### 2.3 Data Analysis

First, we extracted the numbers and age-standardized rates (ASRs) of OM prevalence, incidence, and YLDs at global, regional, and national levels for 1990 and 2021, along with the percentage changes between these time points. To explore the relationship between OM burden and socio-demographic development, we calculated Spearman's rank correlation coefficient (rho) between ASRs and SDI values across 204 countries and territories for 2021. We applied locally weighted regression (LOESS) with a smoothing span of 0.5 to visualize the non-linear relationship patterns between OM burden and SDI.

APC analysis is a fundamental epidemiological framework that simultaneously estimates the effects of age, period, and cohort to decompose disease time trends into independent temporal dimensions (21, 22). We utilized the National Cancer Institute's APC web tool (https://analysistools.cancer.gov/apc/) to analyze the prevalence, incidence, and YLDs of OM across global and five SDI regions. Data were structured in 5-year age groups (from under 5 years to 95 + years) and 5-year time periods (from 1992–1996 to 2017–2021), with birth cohorts derived from the relationship between period and age. The analysis generated four key estimable functions: longitudinal age curves (model-based age-specific rates adjusted for period and cohort effects), period relative risks (rate ratios quantifying temporal changes across calendar periods), cohort relative risks (rate ratios measuring generational differences), and local drifts (age-specific annual percentage changes). Additionally, we calculated net drift values representing the overall annual percentage change as indicators of long-term secular trends.

To identify the theoretical minimum OM burden achievable at each SDI level, we employed Frontier analysis to determine the lowest potentially achievable burden based on development status (23). Unlike traditional regression models, Frontier analysis identifies the theoretical minimum disease burden each country could achieve at its SDI level, establishing benchmarks for optimal performance and quantifying improvement potential. We defined “improvement potential” as the difference between a country's observed burden and the frontier value at its corresponding SDI level. We employed a bootstrap approach with 100 iterations to ensure robust frontier estimation, sampling with replacement and excluding super-efficient observations (outliers) to avoid undue influence on envelopment estimators. The final frontier curve was derived using LOESS regression with a span parameter of 0.2, providing optimal balance between capturing local variations and maintaining smoothness. This approach enabled benchmarking of countries against best performers at their respective SDI levels and comparison of changes in relative efficiency between 1990 and 2021.

Statistical significance was defined as *p* < 0.001 to control for multiple comparisons. Following GBD convention, we report uncertainty intervals (UIs) which capture model uncertainty, while confidence intervals (CIs) are used specifically for percentage change estimates. All statistical analyses were performed using R software (version 4.4.2), with the exception of the APC analysis, which was conducted using the web-based tool.

## 3 Results

### 3.1 Global trends in otitis media disease burden

From 1990 to 2021, the epidemiological characteristics of OM demonstrated significant temporal changes globally. Regarding prevalence, the global number of OM cases increased from 100.32 million in 1990 to 121.23 million cases in 2021, representing a 20.9% increase. However, the age-standardized prevalence rate (ASPR) showed a declining trend, decreasing from 1,794.39 per 100,000 population in 1990 to 1,593.74 per 100,000 population in 2021, representing an 11.18% reduction. Males had a higher ASPR than females (1,672.30 vs. 1,513.31 per 100,000 population in 2021), with both sexes showing similar declining trends (Table 1).

**Table 1:** Prevalence of otitis media in 1990 and 2021.

**Location**	1990		2021		**Percentage change in ASPR from 1990 to 2021 (%, 95% CI)**
	**Number (millions, 95% UI)**	**ASPR (per 100,000, 95% UI)**	**Number (millions, 95% UI)**	**ASPR (per 100,000, 95% UI)**	
Global	100.32 (86.44, 116.08)	1,794.39 (1,540.86, 2,066.35)	121.23 (104.26, 139.98)	1,593.74 (1,374.43, 1,833.01)	−11.18 (−13.13, −9.31)
**Sex**					
Male	53.49 (45.95, 61.95)	1,887.9 (1,623.59,2,168.85)	64.57 (55.46, 74.33)	1,672.3 (1,443.28, 1,925.41)	−11.42 (−13.52, −9.4)
Female	46.83 (40.42, 54.2)	1,700.12 (1,456.31, 1,962.44)	56.66 (48.9, 65.72)	1,513.31 (1,307.78, 1,744.19)	−10.99 (−13.02, −9.02)
**SDI**					
High SDI	9.1 (7.86, 10.57)	1,127.55 (985.05, 1,297.09)	9.18 (7.89, 10.62)	1,014.96 (885.51, 1,158.07)	−9.99 (−11.85, −8.13)
High-middle SDI	16.36 (14.06, 19.12)	1,556.6 (1,340.69, 1,799.29)	14.5 (12.47, 16.97)	1,276.52 (1,110.72, 1,472.22)	−17.99 (−20.7, −15.39)
Middle SDI	34 (29.05, 39.54)	1,866.3 (1,605.74,21,52.38)	34.92 (30.01, 40.52)	1,513.89 (1,313.16, 1,746.04)	−18.88 (−22.06, −15.69)
Low-middle SDI	28.46 (24.52, 33.18)	2,222.9 (1,893.76, 2,559.1)	37.35 (31.92, 43.3)	1,889.25 (1,617.56, 2,177.85)	−15.01 (−17.25, −12.51)
Low SDI	12.32 (10.61, 14.4)	2,210.08 (1,870.5, 2,563.53)	25.19 (21.7, 29.67)	2,046.25 (1,733.15, 2,366.82)	−7.41 (−8.96, −5.93)
**Region**					
High-income Asia Pacific	1.84 (1.570, 2.17)	1,142.52 (991.12, 1,326.44)	1.49 (1.26, 1.72)	1,007.34 (871.46, 1,160.8)	−11.83 (−15.6, −7.12)
High-income North America	2.51 (2.2, 2.87)	981.65 (863.87, 1,107.22)	2.88 (2.51, 3.28)	933.67 (822.78, 1,051.56)	−4.89 (−6.9, −2.75)
Western Europe	3.99 (3.39, 4.67)	1,163.16 (1,001.38, 1,345.11)	3.85 (3.25, 4.53)	1,051.98 (904.93, 1,214.86)	−9.56 (−11.31, −7.9)
Australasia	0.16 (0.14, 0.19)	875.78 (753.61, 1,017.1)	0.21 (0.18, 0.24)	811.13 (704.01, 938.35)	−7.38 (−10.86, −4.42)
Andean Latin America	0.66 (0.57, 0.76)	1,594.48 (1,373.14, 1,850.95)	0.94 (0.81, 1.09)	1,429.39 (1,235.47, 1,652.36)	−10.35 (−713.45, −7.54)
Tropical Latin America	2.5 (2.17, 2.91)	1,538.42 (1,340.1, 1,781.54)	3.02 (2.61, 3.53)	1,423.13 (1,239.68, 1,655.18)	−7.49 (−9.44, −5.38)
Central Latin America	2.87 (2.51, 3.27)	1,566.05 (1,365.91, 1,812.43)	3.63 (3.15, 4.2)	1,483.23 (1,295.56, 1,704.76)	−5.29 (−6.54, −4.01)
Southern Latin America	0.61 (0.52, 0.7)	1,212.56 (1,044.9, 1,398.46)	0.66 (0.57, 0.78)	1,080.67 (931.66, 1,249.58)	−10.88 (−13.59, −8.06)
Caribbean	0.58 (0.5, 0.67)	1,584.86 (1,362.27, 1,825.86)	0.72 (0.62, 0.84)	1,570.79 (1,355.97, 1,819.98)	−0.89 (−2.98, 1.35)
Central Europe	1.67 (1.43, 1.96)	1,398.03 (1,202.53, 1,620.41)	1.2 (1.02, 1.41)	1,212.22 (1,040.46, 1,406.69)	−13.29 (−15.13, −11.38)
Eastern Europe	3.1 (2.7, 3.59)	1462.37 (1276.34,1683.01)	2.5 (2.17, 2.89)	1,414.91 (1,236.75, 1,625.44)	−3.25 (−4.45, −2.18)
Central Asia	1.1 (0.94, 1.28)	1,493.37 (1,267.3, 1,738.92)	1.34 (1.16, 1.57)	1,397.8 (1,200.86, 1,629.87)	−6.4 (−8.41, −4.49)
North Africa and Middle East	6.31 (5.48, 7.33)	1,670.69 (1,434.93, 1,946.75)	9.65 (8.29, 11.22)	1,527.13 (1,317.92, 1,775.13)	−8.59 (−10.43, −6.92)
South Asia	29.5 (25.24, 34.42)	2,445.9 (2,081.33, 2,818.64)	38.05 (32.32, 44.39)	2,048.28 (1,755.16, 2,369.76)	−16.26 (−18.9, −13.39)
Southeast Asia	8.8 (7.56, 10.18)	1,760.94 (1,507.51, 2,024.82)	9.67 (8.29, 11.26)	1,433.26 (1,235.42, 1,661.23)	−18.61 (−21.38, −15.58)
East Asia	23.06 (19.43, 27.23)	1 852.48 (1,590.31, 2,149.96)	17.37 (15, 20.34)	1,335.55 (1,162.35, 1,545.94)	−27.9 (−31.86, −24.02)
Oceania	0.12 (0.1, 0.14)	1,660.62 (1,420.42, 1,926.43)	0.23 (0.2, 0.27)	1,564.23 (1,332.02, 1,817.08)	−5.8 (−9.05, −2.39)
Western Sub-Saharan Africa	4.38 (3.78, 5.13)	2,041.9 (1,730.53, 2,381.44)	10.49 (9.08, 12.24)	1,913.38 (1,622.44, 2,213.17)	−6.29 (−7.74, −4.82)
Eastern Sub-Saharan Africa	4.38 (3.81, 5.13)	2,034.1 (1,720.66, 2,347.05)	8.95 (7.72, 10.49)	1,878.28 (1,604.28, 2,180.55)	−7.66 (−9.46, −5.83)
Central Sub-Saharan Africa	1.17 (1.01, 1.37)	1,899.1 (1,597.13, 2,216.92)	3.03 (2.58, 3.55)	2,028.76 (1,715.76, 2,362.44)	6.83 (2.28, 11.27)
Southern Sub-Saharan Africa	1.01 (0.89, 1.18)	1,708.48 (1,490.8, 1,957.75)	1.36 (1.18, 1.58)	1,661.57 (1,445.55, 1,910.02)	−2.75 (−4.27, −1.31)

UI, uncertainty interval; CI, Confidence interval; ASPR, age-standardized prevalence rate; SDI, Socio-Demographic Index.

Incidence data revealed that global new cases of OM increased from 316.04 million in 1990 to 391.33 million in 2021, representing a 23.8% increase. The age-standardized incidence rate (ASIR) increased from 5,329.29 per 100,000 population in 1990 to 5,529.10 per 100,000 population in 2021, with an increase of 3.75%. Females had a higher ASIR than males (5,750.94 vs. 5,315.08 per 100,000 population in 2021), and both sexes demonstrated increasing trends (Table 2).

**Table 2:** Incidence of otitis media in 1990 and 2021.

**Location**	1990		2021		**Percentage change in ASIR from 1990 to 2021 (%, 95% CI)**
	**Number (millions, 95% UI)**	**ASIR (per 100,000, 95% UI)**	**Number (millions, 95% UI)**	**ASIR (per 100,000, 95% UI)**	
Global	316.04 (233.18, 431.91)	5,329.29 (3,969.64, 7,226.44)	391.33 (292.41, 525.45)	5,529.1 (4,104.7, 7,511.93)	3.75 (3.2, 4.3)
**Sex**					
Male	154.46 (113.47, 211.76)	5,073.74 (3,751.75, 6,916.85)	192.46 (142.61, 260.99)	5,315.08 (3,920.83, 7,247.72)	4.76 (3.99, 5.56)
Female	161.58 (121.01, 219.26)	5,593.29 (4,207.79, 7,537.44)	198.87 (150.13, 265.52)	5,750.94 (4,314.37, 7,768.79)	2.82 (2.23, 3.5)
**SDI**					
High SDI	31.2 (25.07, 39.41)	4,460.37 (3,499.14, 5,738.44)	32.19 (26.42, 39.78)	4,619.95 (3,655.99, 5,920.52)	3.58 (2.16, 6.28)
High-middle SDI	45.08 (34.53, 59.5)	4,580.43 (3,483.63, 6,114.42)	42.5 (33.26, 55.36)	4,605.87 (3,494.03, 6,167.44)	0.56 (0.16, 0.98)
Middle SDI	97.65 (72.51, 133.05)	5,039.84 (3,780.27, 6,804.4)	105.61 (79.92, 141.15)	5,181.53 (3,858.97, 7,027.36)	2.81 (2.03, 3.56)
Low-middle SDI	94.72 (68.2, 131.97)	6,072.51 (4,460.9, 8,292.08)	118.64 (86.9, 161.82)	6,048.17 (4,439.74, 8,243.27)	−0.4 (−0.92, 0.13)
Low SDI	47.15 (33.92, 65.44)	6,179.7 (4,523.54, 8,461.17)	92.12 (66.42, 127.52)	6,133.79 (4,494.81, 8,374.16)	−0.74 (−1.21, −0.25)
**Region**					
High-income Asia Pacific	5.41 (4.27, 6.86)	4,043.21 (3,105.51, 5,286.27)	4.45 (3.61, 5.51)	4,276.84 (3,249.37, 5,598.59)	5.78 (1.88, 25.14)
High-income North America	9.14 (7.76, 10.84)	3,957.1 (3,323.98, 4,736.56)	10.35 (8.99, 11.88)	4,189.18 (3,565.25, 4,940.81)	5.86 (2.7, 9.05)
Western Europe	14.79 (11.5, 19.29)	5,404.35 (4,086.67, 7,312.96)	14.74 (11.55, 19.04)	5,419.03 (4,093.69, 7,336.8)	0.27 (0.08, 0.44)
Australasia	0.85 (0.65, 1.11)	5,043.4 (3,752.26, 6,647.72)	1.1 (0.85, 1.42)	5,042.59 (3,752.06, 6,646.18)	−0.02 (−0.07, 0.03)
Andean Latin America	2.51 (1.83, 3.51)	5,182.76 (3,849.48, 7,118.18)	3.28 (2.46, 4.49)	5,178.6 (3,846.33, 7,111.45)	−0.08 (−0.15, −0.02)
Tropical Latin America	9.78 (7.24, 13.39)	5,662.32 (4,242.24, 7,674.05)	10.65 (8.12, 14.16)	5,656.9 (4,241.6, 7,671.86)	−0.1 (−0.34, 0.11)
Central Latin America	11.44 (8.42, 15.75)	5,438.76 (4,107.87, 7,356.38)	12.12 (9.24, 16.27)	5,431.18 (4,098.97, 7,346.72)	−0.14 (−0.28, −0.02)
Southern Latin America	2.53 (1.88, 3.36)	4,962.77 (3,682.07, 6,561.22)	2.57 (1.96, 3.37)	4,959.19 (3,678.12, 6,557.93)	−0.07 (−0.17, 0.02)
Caribbean	2.03 (1.51, 2.75)	5,182 (3,867.6, 6,961.79)	2.15 (1.63, 2.84)	5,178.41 (3,870.89, 6,932.51)	−0.07 (−0.91, 0.69)
Central Europe	4.28 (3.29, 5.68)	4,000.82 (2,972.65, 5,373.06)	3.04 (2.45, 3.79)	3,922.46 (3,024.1, 5,092.75)	−1.96 (−5.54, 2.7)
Eastern Europe	9.22 (7.02, 12.34)	4,730.39 (3,560.45, 6,428.7)	7 (5.47, 9.19)	4,733.59 (3,560.77, 6,435.95)	0.07 (−0.25, 0.4)
Central Asia	3.64 (2.64, 5.06)	4,383.03 (3,270.49, 5,961.89)	4.25 (3.15, 5.8)	4,380.57 (3,270.21, 5,960.8)	−0.06 (−0.24, 0.14)
North Africa and Middle East	24.09 (17.45, 33.67)	5,263.21 (3,928.29, 7,191.85)	32.82 (24.46, 44.92)	5,232.56 (3,901.12, 7,161.98)	−0.58 (−1.02, −0.22)
South Asia	94.76 (67.78, 132.81)	6,588.57 (4,807.38, 9,048.17)	113.74 (83.4, 155.47)	6,606.1 (4,817.88, 9,077.17)	0.27 (−0.13, 0.61)
Southeast Asia	25.71 (18.9, 35.26)	4,632.39 (3,461.3, 6,256.26)	28.86 (21.48, 39.05)	4,666.98 (3,426.1, 6,369.43)	0.75 (−1.51, 2.98)
East Asia	49.53 (38.09, 66.05)	4,162.39 (3,186.58, 5,587.36)	44.2 (34.5, 57.25)	4,161.14 (3,185.68, 5,589.49)	−0.03 (−0.14, 0.1)
Oceania	0.41 (0.29, 0.57)	4,601.04 (3,371.91, 6,313.11)	0.79 (0.57, 1.1)	4,600.95 (3,373.54, 6,313.74)	0 (−0.14, 0.13)
Western Sub-Saharan Africa	17.82 (12.9, 24.53)	6,049.64 (4,466.57, 8,219.27)	42.44 (30.7, 59.07)	6,063.55 (4,474.64, 8,246.03)	0.23 (0.02, 0.46)
Eastern Sub-Saharan Africa	18.96 (13.53, 26.41)	6,325.1 (4,601.2, 8,566.24)	36.68 (26.2, 50.68)	6,323.13 (4,613.79, 8,546.51)	−0.03 (−0.4, 0.6)
Central Sub-Saharan Africa	4.95 (3.57, 6.88)	5,813.66 (4,287.62, 7,933.15)	11.04 (7.96, 15.36)	5,813.94 (4,287.57, 7,932.96)	0 (−0.03, 0.04)
Southern Sub-Saharan Africa	4.19 (3.05, 5.82)	6,168.15 (4,566.3, 8,390.72)	5.04 (3.72, 6.86)	6,156.35 (4,555.24, 8,367.61)	−0.19 (−0.4, 0.03)

UI, uncertainty interval; CI, confidence interval; ASIR, age-standardized incidence rate; SDI, Socio-Demographic Index.

In terms of disease burden, global YLDs attributed to OM increased from 2.03 million in 1990 to 2.45 million in 2021, representing a 20.7% increase. However, the age-standardized YLDs rate (ASYR) demonstrated a declining trend, decreasing from 36.36 per 100,000 population in 1990 to 32.13 per 100,000 population in 2021, with a reduction of 11.64%. Males had a higher ASYR than females (34.03 vs. 30.18 per 100,000 population in 2021), with comparable declining trends in both sexes (Table 3).

**Table 3:** YLDs of otitis media in 1990 and 2021.

**Location**	1990		2021		**Percentage change in ASYR from 1990 to 2021 (%, 95% CI)**
	**Number (95% UI)**	**ASYR (per 100,000, 95% UI)**	**Number (95% UI)**	**ASYR (per 100,000, 95% UI)**	
Global	2,031,542 (1,179,583, 3,282,828)	36.36 (21.27, 58.24)	2,451,433 (1,428,370, 3,935,796)	32.13 (18.74, 51.51)	−11.64 (−13.85, −9.84)
**Sex**					
Male	1,092,427 (635,179, 1,762,895)	38.58 (22.56, 61.73)	1,317,567 (768,925, 2,123,991)	34.03 (19.86, 54.75)	−11.8 (−14.01, −9.82)
Female	939,115 (545,945, 1,515,832)	34.12 (19.96, 54.7)	1,133,866 (659,169, 1,825,543)	30.18 (17.58, 48.26)	−11.53 (−13.75, −9.46)
**SDI**					
High SDI	177,568 (103,621, 284,590)	21.73 (12.6, 34.72)	178,321 (104,694, 286,644)	19.29 (10.99, 30.79)	−11.22 (−13.25, −9.27)
High-middle SDI	328,941 (191,975, 530,287)	31.17 (18.33, 49.96)	289,745 (169,152, 466,888)	25.26 (14.53, 40.56)	−18.98 (−22, −16.32)
Middle SDI	690,157 (399,476, 1,120,539)	37.86 (22.19, 60.57)	704,498 (411,592, 1,141,091)	30.38 (17.69, 48.88)	−19.77 (−23.19, −16.73)
Low-middle SDI	583,306 (335,770, 935,856)	45.62 (26.7, 73.53)	763,239 (445,040, 1,231,234)	38.47 (22.39, 61.79)	−15.67 (−18.03, −13.28)
Low SDI	250,026 (144,716, 399,362)	45.15 (26.54, 72.42)	513,909 (298,220, 826,506)	41.78 (24.43, 67.13)	−7.46 (−9.19, −5.69)
**Region**					
High-income Asia Pacific	36,333 (21,316, 59,931)	22.17 (12.89, 36.17)	29,172 (17,158, 47,347)	19.22 (10.96, 30.76)	−13.3 (−17.19, −9.6)
High-income North America	48,713 (28,613, 78,690)	18.84 (10.96, 30.6)	55,705 (32,767, 89,731)	17.72 (10.14, 28.67)	−5.97 (−8.32, −3.8)
Western Europe	76,808 (44,874, 121,349)	21.85 (12.56, 34.57)	73,777 (43,332, 119,075)	19.52 (11.19, 30.93)	−10.67 (−12.81, −8.71)
Australasia	3,020 (1,707, 4,929)	16.27 (9.18, 26.44)	3,842 (2,221, 6,084)	14.89 (8.49, 23.91)	−8.49 (−13.75, −3.62)
Andean Latin America	12,546 (7,220, 20,232)	30.97 (17.95, 50.1)	18,176 (10,648, 29,150)	27.6 (16.21, 44.4)	−10.85 (−14.99, −6.67)
Tropical Latin America	49,766 (28,757, 80,826)	30.61 (17.75, 49.08)	59,984 (35,118, 97,042)	28.14 (16.32, 45.27)	−8.05 (−10.68, −5.51)
Central Latin America	56,119 (32,815, 90,802)	30.89 (17.94, 49.38)	71,814 (41,969, 115,037)	29.18 (16.98, 46.66)	−5.55 (−7.39, −3.84)
Southern Latin America	11,704 (6,833, 18,602)	23.35 (13.65, 36.96)	12,815 (7,583, 20,409)	20.56 (11.97, 32.96)	−11.95 (−16.01, −7.46)
Caribbean	11,491 (6,658, 18,623)	31.4 (18.32, 49.93)	14,269 (8,333, 22,874)	31.07 (18.17, 49.54)	−1.06 (−3.82, 1.84)
Central Europe	33,939 (19,855, 55,033)	28.27 (16.57, 45.39)	24,058 (14,000, 38,873)	24.31 (14.06, 39.27)	−13.99 (−16.29, −11.78)
Eastern Europe	62,531 (36,568, 100,826)	29.53 (17.32, 47.41)	50,325 (29,460, 81,666)	28.5 (16.66, 45.52)	−3.49 (−5.13, −1.83)
Central Asia	22,106 (12,986, 36,110)	30.14 (17.83, 48.81)	27,041 (15,671, 44,029)	28.11 (16.32, 45.68)	−6.72 (−9.65, −3.87)
North Africa and Middle East	127,597 (74,972, 203,587)	33.9 (19.85, 53.68)	195,271 (113,986, 307,902)	30.83 (17.97, 48.65)	−9.05 (−11.28, −7.12)
South Asia	607,766 (347,888, 974,556)	50.37 (29.42, 81.23)	780,621 (454,125, 1,263,651)	41.77 (24.34, 67.24)	−17.08 (−20.03, −14.28)
Southeast Asia	180,460 (105,590, 291,045)	36.1 (21.17, 57.77)	196,491 (113,262, 317,392)	29.01 (16.72, 46.67)	−19.64 (−22.72, −16.55)
East Asia	467,492 (270,533, 758,666)	37.41 (21.84, 59.59)	348,986 (204,080, 567,549)	26.58 (15.35, 43)	−28.96 (−33.43, −25.02)
Oceania	2,366 (1,388, 3,847)	33.17 (19.42, 53.7)	4,628 (2,657, 7,568)	31.18 (18.09, 50.7)	−6 (−10.45, −1.49)
Western Sub-Saharan Africa	90,602 (52,428, 144,311)	42.39 (24.77, 68.13)	217,612 (127,359, 348,460)	39.66 (23.28, 63.77)	−6.44 (−8.08, −4.8)
Eastern Sub-Saharan Africa	86,825 (50,644, 139,655)	40.85 (23.87, 65.34)	178,306 (104,417, 287,547)	37.65 (22.1, 60.49)	−7.83 (−9.92, −5.89)
Central Sub-Saharan Africa	23,524 (13,726, 37,475)	38.45 (22.38, 61.98)	61,712 (35,650, 98,694)	41.41 (24.14, 66.66)	7.71 (1.79, 13.45)
Southern Sub-Saharan Africa	19,833 (11,662, 32,039)	33.65 (19.79, 54.05)	26,828 (15,713, 43,306)	32.59 (19.15, 52.27)	−3.17 (−5.36, −1.28)

UI, uncertainty interval; CI, confidence interval; ASYR, age-standardized YLDs rate; SDI, Socio-Demographic Index.

### 3.2 Socio-Demographic Index level impact

Regions with different Socio-Demographic Index (SDI) levels demonstrated significant disparities in OM disease burden. In terms of prevalence, Low SDI regions had the highest ASPR in 2021 at 2,046.25 per 100,000 population, followed by Low-middle SDI regions at 1,889.25 per 100,000 population, while High SDI regions had the lowest ASPR at 1,014.96 per 100,000 population. Regarding temporal trends, all SDI level regions showed declining trends in ASPR, but with varying magnitudes of reduction. Middle SDI regions demonstrated the largest reduction at 18.88%, followed by High-middle SDI regions at 17.99%, while Low SDI regions showed the smallest reduction at 7.41% (Table 1).

Incidence data showed that Low SDI regions had the highest ASIR in 2021 at 6,133.79 per 100,000 population, followed by Low-middle SDI regions at 6,048.17 per 100,000 population, while High-middle SDI regions had the lowest ASIR at 4,605.87 per 100,000 population. In terms of temporal trends, High SDI, High-middle SDI, and Middle SDI regions all showed increasing trends in ASIR, with increases of 3.58%, 0.56%, and 2.81%, respectively, while Low-middle SDI and Low SDI regions demonstrated declining trends with reductions of 0.40 and 0.74%, respectively (Table 2).

### 3.3 Regional variations

Significant regional disparities were observed in OM disease burden across different geographical areas in 2021. For prevalence, South Asia demonstrated the highest ASPR at 2,048.28 per 100,000 population, followed by Central Sub-Saharan Africa at 2,028.76 per 100,000 population and Western Sub-Saharan Africa at 1,913.38 per 100,000 population. In contrast, Australasia had the lowest ASPR at 811.13 per 100,000 population, followed by High-income North America at 933.67 per 100,000 population and High-income Asia Pacific at 1,007.34 per 100,000 population. Regarding temporal trends in ASPR from 1990 to 2021, East Asia showed the largest reduction at 27.90%, followed by Southeast Asia at 18.61 and South Asia at 16.26%. Notably, Central Sub-Saharan Africa was the only region to demonstrate an increase in ASPR, with a 6.83% rise (Table 1).

For ASIR in 2021, South Asia maintained the highest burden at 6,606.10 per 100,000 population, while Central Europe had the lowest at 3,922.46 per 100,000 population. Regarding temporal trends in ASIR, High-income North America and High-income Asia Pacific showed notable increases of 5.86 and 5.78% respectively, while most other regions demonstrated relatively modest changes ranging from small decreases to increases of less than 2% (Table 2).

YLDs analysis revealed that South Asia had the highest ASYR at 41.77 per 100,000 population, followed by Central Sub-Saharan Africa at 41.41 per 100,000 population and Western Sub-Saharan Africa at 39.66 per 100,000 population. Australasia demonstrated the lowest ASYR at 14.89 per 100,000 population, followed by High-income North America at 17.72 per 100,000 population and High-income Asia Pacific at 19.22 per 100,000 population (Table 3).

### 3.4 Country-level disease burden rankings

At the country level, substantial variations in OM burden were evident in 2021. For prevalence, Somalia ranked highest globally with an exceptionally high rate of 2,925.50 per 100,000 population, while Monaco, Australia, and Singapore demonstrated the lowest rates (Supplementary Table S1). For incidence, Pakistan led the rankings, followed by Spain and Ethiopia, while Taiwan (Province of China) had the lowest rates (Supplementary Table S2). YLDs patterns showed similar distribution to prevalence, with Somalia again ranking highest (60.21 per 100,000 population) and Monaco showing the lowest burden (Supplementary Table S3).

**Figure 1:**
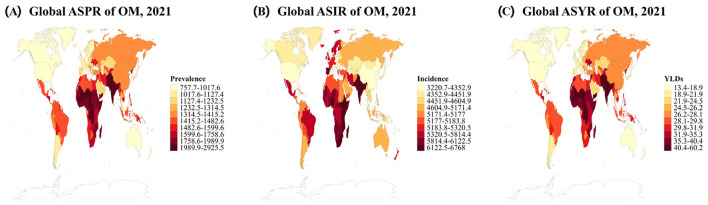
Global distribution of age-standardized rates of otitis media (OM) burden in 2021. **(A)** Age-standardized prevalence rates (ASPR) per 100,000. **(B)** Age-standardized incidence rates (ASIR) per 100,000 population. **(C)** Age-standardized years lived with disability rates (ASYR) per 100,000 population. Maps display data from 204 countries using 10-level color gradient from light yellow (lowest rates) to dark red (highest rates).

The global distribution maps revealed distinct geographical clustering patterns, with Sub-Saharan African and South Asian countries consistently showing the highest burden levels across all three metrics, while high-income countries in Europe, North America, and Asia-Pacific regions demonstrated substantially lower burden levels (Figure 1). This geographical pattern remained remarkably consistent across prevalence, incidence, and YLDs, indicating clear regional disparities in OM disease burden.

### 3.5 Correlation between socio-demographic development and disease burden

**Figure 2:**
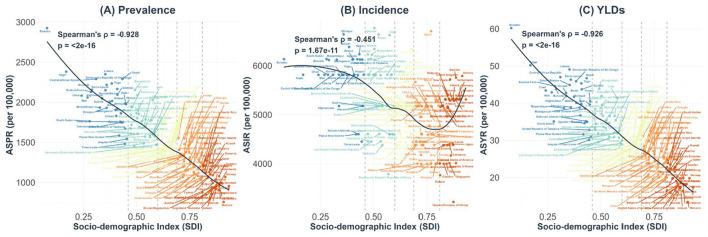
Correlation between socio-demographic index and age-standardized rates of otitis media burden in 204 countries and territories, 2021. Scatter plots illustrating the relationship between the Socio-demographic Index (SDI) and three metrics of otitis media burden: for **(A)** Age-standardized prevalence rates (ASPR), **(B)** Age-standardized incidence rates (ASIR), and **(C)** Age-standardized years lived with disability (YLDs) rates (ASYR), all age-standardized per 100,000 population. Countries are stratified into five SDI quintiles and color-coded accordingly: low SDI (<0.46), low-middle SDI (0.46–0.60), middle SDI (0.61–0.69), high-middle SDI (0.70–0.81), and high SDI (>0.81). The fitted curves represent locally weighted scatterplot (LOESS) regression. Vertical dashed lines denote SDI category thresholds. Spearman's rank correlation coefficients (ρ) and corresponding *p*-values are presented in each panel.

The scatter plot analysis revealed strong inverse correlations between Socio-Demographic development and otitis media disease burden across 204 countries and territories in 2021 (Figure 2). For prevalence, a robust negative correlation was observed between SDI and ASPR (Spearman's ρ = −0.928, *p* < 2e−16), indicating that countries with higher socioeconomic development generally demonstrated lower otitis media prevalence.

The correlation between SDI and ASIR was relatively weaker but still statistically significant (Spearman's ρ = −0.451, *p* = 1.67e−11), suggesting a moderate negative relationship between socioeconomic development and disease incidence. Notably, the fitted curve showed that in High SDI regions, ASIR actually increased with rising SDI levels, indicating a distinct pattern where more developed countries may experience higher incidence despite lower prevalence and YLDs. For YLDs, the correlation was similar to that of prevalence, showing a strong negative association with SDI (Spearman's ρ = −0.926, *p* < 2e−16).

### 3.6 Age-period-cohort analysis

#### 3.6.1 Temporal trends and net drift

The APC analysis revealed significant temporal trends in otitis media burden across different SDI levels from 1992 to 2021 (Supplementary Table S4). Net drift, representing the overall annual percentage change, showed that globally, prevalence demonstrated a significant annual decline of 0.661% per year, while incidence showed a modest annual increase of 0.032% per year. YLDs exhibited a similar declining pattern to prevalence with a net drift of 0.670% per year.

The temporal trends varied substantially across SDI levels. Middle SDI regions showed the steepest annual decline in prevalence (−1.033% per year) and YLDs (−1.054% per year), followed by High-middle SDI regions with annual decreases of 0.975 and 0.991% respectively. In contrast, Low SDI regions demonstrated the smallest annual reductions in both prevalence (−0.414% per year) and YLDs (−0.403% per year). For incidence, High-middle SDI regions showed the largest annual decline (−0.071% per year), followed by High SDI regions with a slight annual decline (−0.018% per year), while Middle SDI regions exhibited a modest annual increase (0.034% per year).

#### 3.6.2 Age effects

**Figure 3:**
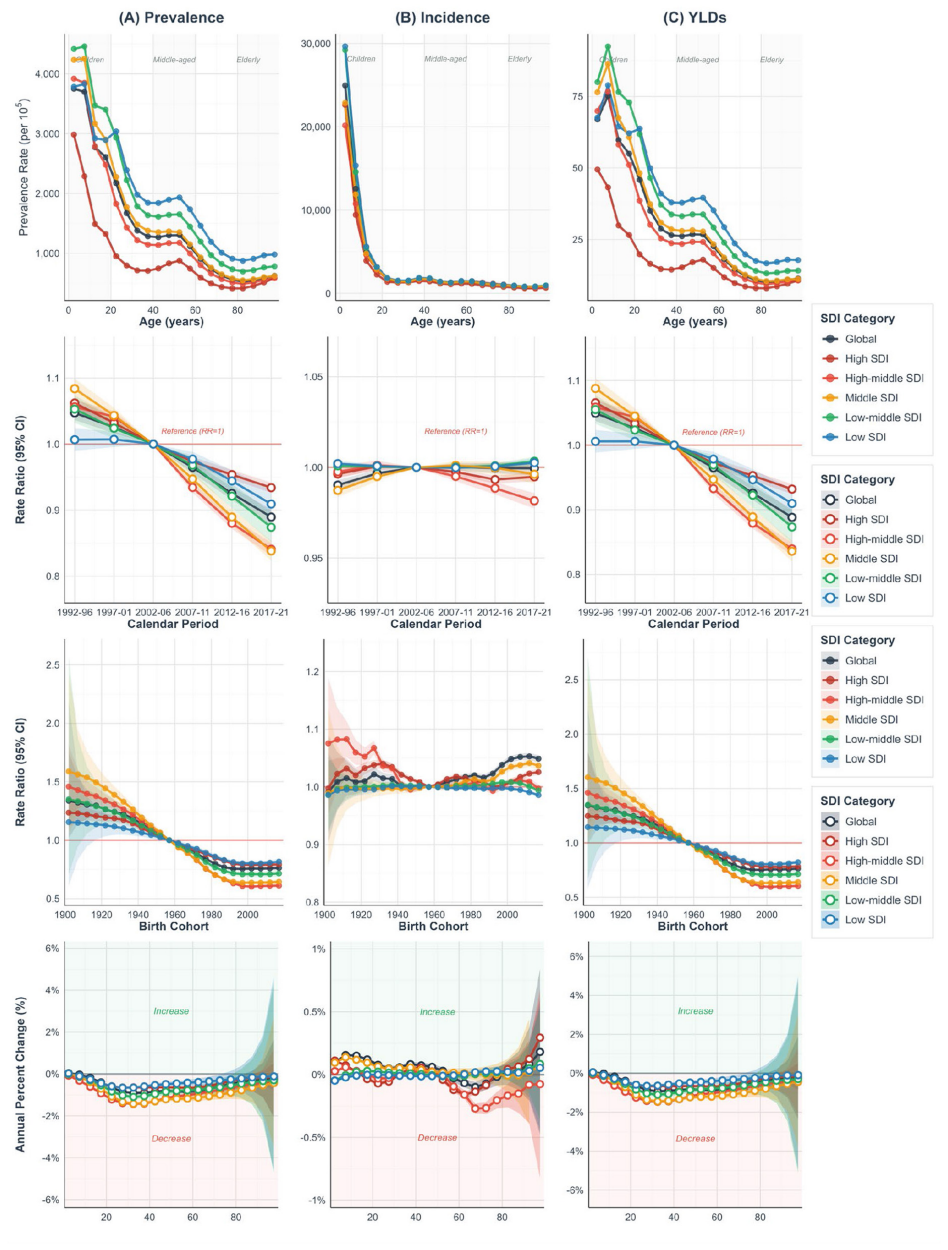
Age-period-cohort analysis of otitis media disease burden across global and Socio-demographic index regions, 1992-2021. This figure presents APC analysis for three indicators: **(A)** Prevalence, **(B)** Incidence, and **(C)** Years lived with disability (YLDs) across global and five Socio-Demographic Index (SDI) regions. Row 1 shows longitudinal age curves with shaded regions indication pediatric, middle-aged, and geriatric populations. Row 2 displays period rate ratios across six 5-year intervals (reference period: 2007–2011, *RR* = 1.0). Row 3 presents cohort rate rations for birth cohorts 1900–2020. Row 4 illustrates local drifts showing age-specific annual percent change, with green/red backgrounds indicating increasing-decreasing trends. Lines represent Global (dark blue) and SDI quintiles. High (Dark red), High-middle (red), Middle (orange), Low-middle (green), and Low (blue). Shaded areas indicate 95% UI.

The longitudinal age curves revealed distinct age-related patterns in otitis media burden (Figure 3, Row 1; Supplementary Table S6). For prevalence, the disease peaked in early childhood, with the highest age-standardized rate observed in the 0–4 age group at 3,750.64 per 100,000 population. This was followed by a rapid decline to 1,379.41 per 100,000 population in the 30–34 age group, and a gradual decrease to 905.09 per 100,000 population in the 60–64 age group, before slightly increasing in the oldest age groups. All five SDI regions exhibited similar age trends, demonstrating the intrinsic stability of age effects. However, a clear gradient was observed across SDI levels, with an overall downward shift across all age groups as SDI levels increased. This SDI gradient was most pronounced in childhood and gradually diminished with advancing age.

Incidence patterns showed even more dramatic age effects. Globally and across all SDI levels, incidence rates were extremely high in early childhood, peaking at 24,966.29 per 100,000 population in the 0–4 age group, then dropping sharply to 4,891.37 per 100,000 population in the 10–14 age group, and stabilizing at much lower levels in adulthood. Similar to prevalence, incidence demonstrated significant SDI-related differences, particularly evident in childhood, with these differences gradually diminishing and almost disappearing after age 20 years. The age distribution pattern of YLDs was similar to prevalence, with the highest burden in early childhood (66.99 per 100,000 population in the 0–4 age group) and showing a general declining trend with advancing age.

#### 3.6.3 Period effects

All SDI levels demonstrated clear declining period trends, but with significant differences in the magnitude of decline (Figure 3, Row 2; Supplementary Table S6). Globally, prevalence showed consistent decline from 1992 to 2021, with rate ratios decreasing from 1.047 to 0.889. Middle SDI regions exhibited the steepest decline (from 1.084 to 0.838, a 22.7% reduction), followed by High-middle SDI regions (from 1.056 to 0.841, a 20.4% reduction), while Low SDI regions showed the smallest decline (from 1.007 to 0.909, a 9.7% reduction).

Incidence period effects were more complex across different SDI levels. High-middle SDI regions demonstrated the most pronounced declining trend (from 0.998 to 0.982), while High SDI regions showed relatively stable patterns with a notable uptick in the final period (2017–2021), rising from 0.993 to 0.995. YLDs period effect patterns were highly consistent with prevalence across all SDI levels.

#### 3.6.4 Cohort effects

Birth cohort analysis revealed significant generational differences in otitis media burden, with consistent patterns across all SDI levels (Figure 3, Row 3; Supplementary Table S6). Globally, compared to the reference cohort (born in 1957), earlier birth cohorts (born around 1897–1922) showed higher disease burden, with prevalence cohort rate ratios ranging from 1.35 to 1.09. Recent birth cohorts (born after 1962) demonstrated progressively lower burden, with cohort rate ratios declining to approximately 0.76 for the most recent cohorts.

All SDI levels exhibited similar cohort effect patterns, with earlier generations showing higher burden and recent generations showing lower burden. High-middle SDI regions displayed the most pronounced cohort effects, with early cohort prevalence rate ratios reaching 1.49 and recent cohorts as low as 0.61. Notably, High SDI regions showed a slight upturn in recent birth cohorts, with the most recent cohorts displaying slightly higher rates compared to previous cohorts.

Incidence cohort effects differed markedly from prevalence patterns. Globally, early birth cohorts (1897–1912) had relatively low incidence cohort rate ratios (0.96–1.02), which gradually increased over time but showed sustained decline during 1932–1947, then began to increase again. Recent birth cohorts (1997–2017) showed a sustained upward trend, reaching cohort rate ratios of 1.02–1.05. This fluctuating pattern was more evident in High, High-middle, and Middle SDI regions, while the fluctuation was less apparent in Low and Low-middle SDI regions, indicating opposite generational trends between incidence and prevalence. YLDs cohort effect patterns were consistent with prevalence, showing gradual improvement from early to recent periods.

#### 3.6.5 Local drift

Age-specific local drift analysis revealed complex heterogeneous temporal trends across different age groups and SDI levels (Figure 3, Row 4; Supplementary Table S6). For prevalence, most age groups globally showed declining trends, but the magnitude of improvement was relatively smaller at both ends of the age spectrum (infants and older adult populations). School-age children and adolescents (10–29 years) demonstrated the most significant declining trends, with annual percentage changes ranging from −0.18 to −0.85%. Notably, the 0–4 year age group showed slight burden increases in global, Middle SDI, and Low SDI regions.

Incidence local drift patterns were distinctly different and more complex. Globally, all age groups from 0 to 54 years showed increasing trends, with positive local drifts (0.037%−0.151%), indicating rising incidence rates. Age groups after 55–59 years showed negative trends, indicating declining incidence rates, except in the very older adult groups (80–95 + years) where positive values reappeared.

Local drift patterns differed significantly across SDI levels. Low and Low-middle SDI regions showed relatively small fluctuations across all age groups, while other regions demonstrated larger variations. High, High-middle, and Middle SDI regions all showed upward trends in incidence rates during infancy and early childhood. Among older adult patients, High-middle SDI regions showed a clear declining trend, whereas High SDI regions showed a clear upward trend after age 80 years.

### 3.7 Frontier analysis results

#### 3.7.1 Efficiency frontier characteristics

**Figure 4:**
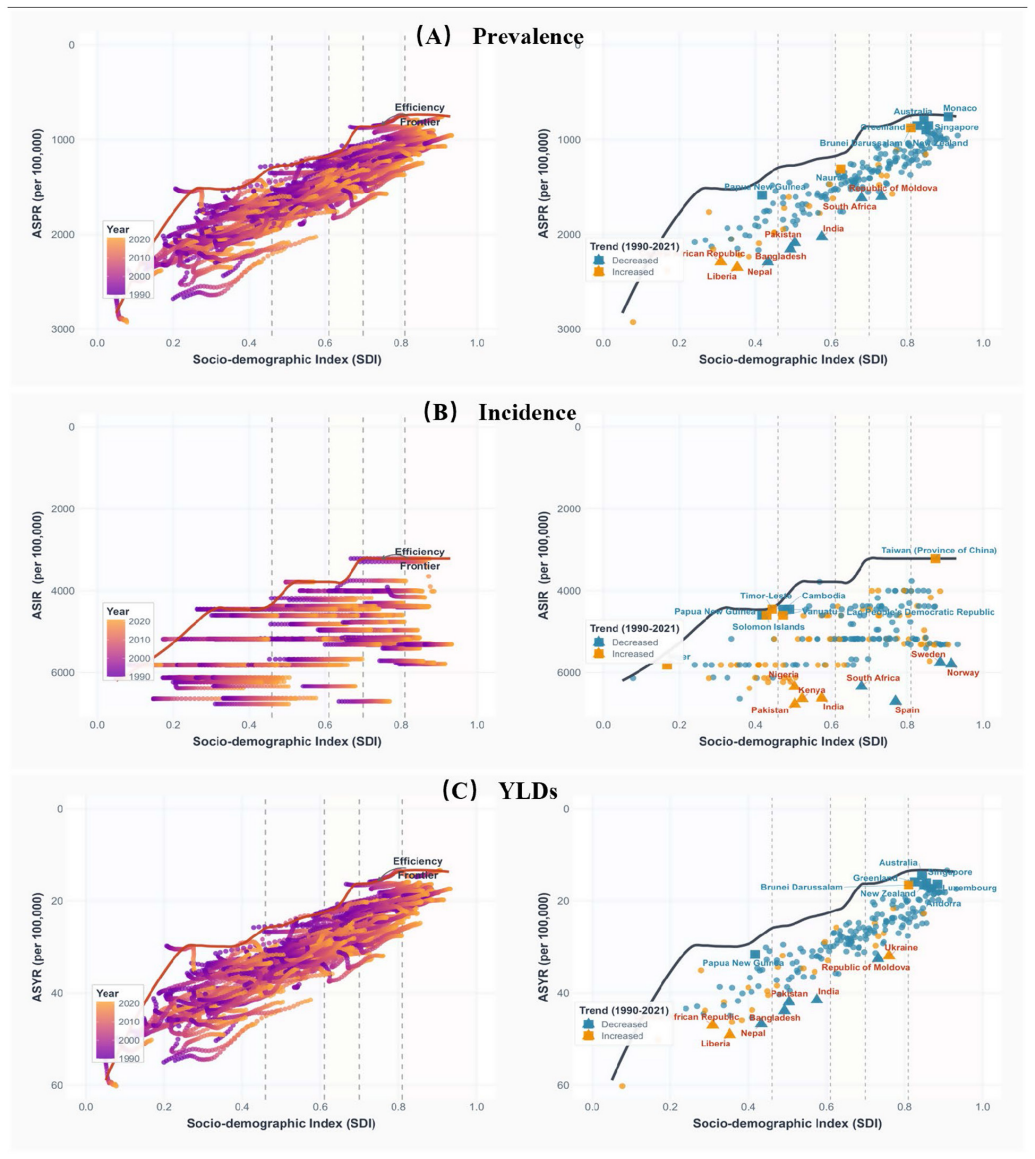
Frontier analysis of global otitis media burden by Socio-demographic Index, 1990–2021. Efficiency frontier analysis for **(A)** Age-standardized prevalence rates (ASPR), **(B)** Age-standardized incidence rates (ASIR), and **(C)** Age-standardized years lived with disability (YLDs) rates (ASYR). Left panels show temporal trends (1990–2021) with color gradient indicating year. Right panels show 2021 data with countries colored by trend (blue = decrease, orange = increase since 1990), Countries furthest from (red triangles) and closest to (blue squares) the efficiency frontier are labeled. The black curve represents the efficiency frontier calculated through data envelopment analysis (100 bootstrap iterations. Vertical demarcate SDI quintiles. All rates are age standardized per 100,000 population.

Based on data from 204 countries and territories, this study constructed efficiency frontiers for prevalence, incidence, and YLDs, revealing the optimal conditions at different SDI levels (Figure 4; Supplementary Table S7). Prevalence and YLDs frontiers both exhibited smooth negative slope declining patterns, though with different magnitudes. The frontier of prevalence showed a non-linear characteristic with frontier values dropping sharply from 2642.92 per 100,000 population in the Low SDI range (0.077–0.4) to approximately 1,520 per 100,000 population, continuing to decline to approximately 800 per 100,000 population in the Medium SDI range (0.4–0.8), and converging to 741.17 per 100,000 population in the High SDI range (0.8–0.933).

Similarly, the YLDs frontier demonstrated a relatively smooth decline from approximately 25–30 per 100,000 population in Low SDI areas to 13.4–16.4 per 100,000 population in High SDI areas, maintaining stable declining slopes throughout the SDI spectrum. In stark contrast, the incidence frontier demonstrated a distinctive “step-wise” distribution pattern, exhibiting three distinct horizontal intervals: High SDI interval (SDI > 0.6, frontier values stabilized at 3,219.12 per 100,000 population), Medium SDI interval (SDI 0.4–0.6, frontier values approximately 3,788–4,200 per 100,000 population), and Low SDI interval (SDI <0.4, frontier values approximately 4,450 per 100,000 population), indicating that the relationship between incidence and SDI exhibits distinct segmented characteristics rather than continuous linear patterns.

#### 3.7.2 Efficiency distribution characteristics

The efficiency distributions of the three indicators exhibited varying degrees of heterogeneity and differentiated patterns. For prevalence and YLDs, efficiency distributions followed conventional development patterns where high-income countries generally outperformed low-income countries. Prevalence top performers were led by Monaco (effective difference 16.26 per 100,000 population), Australia (51.75 per 100,000 population), and Singapore (109.98 per 100,000 population), while worst performers included Nepal (929.46 per 100,000 population), Bangladesh (876.18 per 100,000 population), and India (831.37 per 100,000 population). YLDs showed similar patterns with Monaco (0.008 per 100,000 population), Australia (1.063 per 100,000 population), and Greenland (2.396 per 100,000 population) leading efficiency rankings, while Nepal (19.906 per 100,000 population), Liberia (19.247 per 100,000 population), and Bangladesh (18.366 per 100,000 population) exhibited the largest efficiency gaps.

The incidence efficiency distribution exhibited a remarkable “inverse development gradient” characteristic, contrary to conventional expectations. The best performers were primarily low-income countries such as Democratic People's Republic of Korea (effective difference 0 per 100,000 population), Somalia (0.09 per 100,000 population), and Niger (1.49 per 100,000 population), with these countries nearly achieving theoretical optimal levels. Conversely, many high-income developed countries demonstrated significant efficiency losses, with countries such as Spain, Sweden, and the United Kingdom showing effective differences exceeding 2,000 per 100,000 population, substantially higher than the theoretical frontier values corresponding to their development levels.

## 4 Discussion

### 4.1 Dual trends in global disease burden

Our comprehensive analysis using the GBD database revealed a key dual trend in the global burden of OM from 1990 to 2021: with global population growth, absolute disease numbers increased significantly (total OM cases by 20.9%, incident cases by 23.8%, and YLDs by 20.7%); yet after controlling for demographic changes, ASPR and ASYR decreased by 11.18 and 11.64% respectively, indicating declining population-level disease risk that reflects improved healthcare systems and preventive measures.

Particularly noteworthy is our finding that ASIR increased by 3.75%, forming a stark contrast with the declining ASPR. This epidemiological paradox reveals the complexity of OM disease dynamics: the occurrence rate of new cases is rising while overall prevalence levels are declining. Even more thought-provoking is that this trend shows significant differences across regions with different socioeconomic development levels, especially the emergence of a “reverse development gradient” phenomenon in High-SDI regions—incidence rates increase with higher socioeconomic development levels, contrary to traditional disease epidemiological patterns. These findings collectively suggest that the global epidemiological pattern of OM is undergoing profound transformation, possibly related to multiple factors including modern lifestyle changes, medical practice evolution, and demographic transitions. Comprehensive understanding of these complex trends requires integration of multi-dimensional epidemiological indicators and stratified analytical approaches to accurately grasp the spatiotemporal evolution characteristics of disease burden and the underlying driving mechanisms.

### 4.2 Association between socioeconomic development and disease burden

Our study demonstrates a strong negative correlation between the SDI and OM burden, consistent with previous literature on the impact of socioeconomic factors on ear diseases (24, 25). Low-SDI regions had the highest prevalence, while High-SDI regions had the lowest prevalence, indicating the crucial role of socioeconomic development in reducing OM burden. In our APC analysis, the consistent pattern of decreasing prevalence and YLD across all age groups with increasing SDI levels further validates the robustness of this association.

Socioeconomic development may affect OM burden through multiple pathways: higher income levels are typically associated with better sanitation, nutrition, and housing conditions, which can reduce the risk of upper respiratory tract infections that lead to OM (26–28); developed regions generally have more comprehensive healthcare systems and higher-quality medical services, enabling timely and effective treatment and preventing progression to chronic states (29–31); improved education levels can promote better health behaviors and disease prevention awareness, including good personal hygiene and timely medical care (32–34).

However, the “reverse development gradient” phenomenon we observed deserves special attention: incidence rates in High-SDI regions increase with rising SDI, contrary to traditional expectations. This may stem from several factors: the superior diagnostic capabilities and medical record systems in high-income countries may lead to higher reporting rates (35–37); modern lifestyle transitions also constitute driving forces for actual infection risk: the widespread adoption of daycare centers has intensified respiratory pathogen transmission among children (38). Additionally, allergic rhinitis is more prevalent in High-SDI regions, a factor significantly associated with increased OM risk (39–41).

### 4.3 From age-socioeconomic interaction mechanisms to precision prevention and control strategies: differentiated intervention needs revealed by SDI-stratified APC analysis

Our APC analysis goes beyond simple epidemiological description, helping to explore the multi-level mechanisms of OM burden evolution, including the combined effects of biological factors (age effects), socio-historical changes (cohort effects), and healthcare systems and public health interventions (period effects) on disease prevalence patterns. Particularly, through the SDI-stratified analysis perspective, we were able to reveal differentiated patterns in disease burden evolution across regions with different socioeconomic development levels, providing an empirical basis for targeted prevention and control strategies.

#### 4.3.1 Age effects: childhood as the critical period for socioeconomic impact on OM burden

Age effects showed characteristic patterns of early childhood peaks followed by stable decline in adulthood, possibly reflecting the important influence of human physiological development on disease susceptibility. This pattern aligns with anatomical research findings: factors such as the horizontal position of the eustachian tube in infants and toddlers, shorter length, incomplete cartilage development, and adenoid hypertrophy collectively affect its function, while with growth and development, these anatomical features gradually mature and disease risk correspondingly decreases (42, 43). An enlightening finding in the study is that the impact of socioeconomic factors on OM burden is primarily concentrated in childhood. Different SDI regions showed significant burden differences during childhood, with childhood OM burden significantly reduced as SDI levels increased, while these differences almost completely disappeared after age 20. This phenomenon suggests that socioeconomic factors (such as healthcare accessibility, sanitary conditions, and nutritional status) have important effects on childhood OM risk, while adult OM may be primarily dominated by intrinsic biological factors. A study utilizing US commercial insurance data demonstrated that lower socioeconomic status not only increases the risk of disease in children but is also closely associated with undertreatment and elevated complication risk (31). A recent systematic review further confirmed that socioeconomic deprivation consistently increases the risk of otitis media in children, with disparities evident in both disease prevalence and access to surgical interventions across multiple populations (44).

From a public health perspective, this finding highlights the potential value of focusing on childhood OM burden globally, especially for Low-SDI regions, because children not only face the highest disease risk but can also achieve the most significant health benefits through appropriate interventions, with higher cost-effectiveness.

#### 4.3.2 Period effects: regional imbalance between improvement potential and improvement velocity

Period effect analysis further revealed the temporal evolution characteristics of global OM burden and regional differences. From 1992 to 2021, global prevalence decreased from 1.047 to 0.889, but this improvement was not evenly distributed. The middle-SDI region showed the largest decrease (22.7%), while the Low-SDI region showed the smallest decrease (9.7%). This differentiated period effect reflects regional imbalances in healthcare system development and public health interventions. Combined with efficiency Frontier analysis results, we found that although Low-SDI regions have the greatest theoretical improvement potential (greatest distance from the efficiency frontier), their actual improvement rate is relatively slow, possibly related to resource constraints and health system capacity building challenges. This finding suggests that effective interventions targeting Low-SDI regions may have significant marginal benefits, providing reference for optimal allocation of global health resources.

#### 4.3.3 Cohort effects: declining prevalence alongside rising incidence

Cohort effects showed that compared to the reference cohort born in 1957, early birth cohorts (1897–1922) had higher disease burden (cohort rate ratios 1.09–1.35), while recent cohorts (after 1962) showed a continuous trend of burden reduction (cohort rate ratios approximately 0.76). This improvement trend across multiple generations showed surprising consistency across all SDI regions, indicating the universality of global health progress, albeit at varying speeds. However, incidence cohort effects presented a markedly different pattern: recent birth cohorts (1997–2017) showed an upward trend (cohort rate ratios 1.02–1.05), forming a stark contrast with the continuous decline in prevalence. This contradictory phenomenon of decreasing prevalence but increasing incidence reflects the dual effect of modern medicine on OM: on one hand, improved treatment methods successfully shortened disease duration and reduced symptom severity, leading to decreased overall prevalence; on the other hand, the occurrence rate of initial infections did not show corresponding reduction and even increased somewhat. This finding is consistent with the results of Uraguchi et al., who demonstrated in their nationwide longitudinal study of Japanese children that the cumulative incidence of OM was notably higher in the 2010 birth cohort compared to the 2001 birth cohort, despite the introduction of pneumococcal conjugate vaccines (45). Of course, this increase in incidence may also be partially attributable to improved diagnostic efficiency, including advances in otoscopic techniques, increased parental awareness of ear infections, enhanced healthcare access, and more standardized diagnostic criteria leading to better case ascertainment (37).

This phenomenon is consistent with the “reverse development gradient” discussed earlier but provides supplementary evidence from a temporal dimension in cohort analysis. The increased incidence in newer generations may be related to their unique experiences growing up in modern environments and medical conditions, including increased exposure to collective childcare environments during infancy, exposure to new environmental pathogens, and potential antibiotic-resistant strain problems in the context of widespread antibiotic use.

#### 4.3.4 Local drift analysis: the prevention and control dilemma in infants and toddlers

Local drift analysis revealed more refined temporal trend changes from an age dimension, showing differential responses of different age groups to OM prevention and control measures. Prevalence local drift showed that school-age children and adolescents (10–30 years) achieved the most significant improvement (−0.18%– −0.85%), while the highest burden 0–5 age group showed upward trends in multiple SDI regions. This age-specific pattern suggests that despite infants and toddlers having prevalence rates as high as 3,750.64/100,000 (far higher than other age groups), existing prevention and control measures may have relatively limited effectiveness for this high-burden group. Incidence local drift further revealed more widespread age-specific patterns: globally, a broad age range of 0–55 years showed upward trends (0.037%−0.151%), while age groups above 55 showed downward trends (except for very older adult populations). This differential trend between age groups suggests that current prevention and control strategies may not fully consider the age distribution characteristics of the disease, requiring more attention to populations showing upward trends, particularly strengthening early childhood treatment and prevention measures from birth to middle age.

Integrating these APC analysis findings, we observed that OM burden presents complex dynamic change patterns, influenced by the combined effects of age physiological characteristics, socioeconomic development levels, and historical periods. Particularly, the interaction pattern between SDI and age (SDI differences mainly affecting children rather than adults) provides an important perspective for understanding how socioeconomic factors shape disease burden. The study also revealed noteworthy differentiated prevention and control needs. Low-SDI regions have the heaviest burden and greatest improvement potential but relatively slow improvement rates, requiring strengthened basic medical services and health infrastructure. High-SDI regions, while achieving significant success in prevalence control, face challenges of rising incidence rates, showing “reverse development gradient” phenomena. These rising incidence rates require identification and reduction of contributing factors, which may include antibiotic use patterns, collective childcare environments, and risk factors related to modern lifestyles such as allergic diseases. Additionally, improved diagnostic technology and enhanced medical awareness may lead to more cases being detected. However, these hypotheses require further research verification. Meanwhile, early childhood, as the age group with the heaviest disease burden, shows less than ideal improvement trends, highlighting the importance of strengthening infant and toddler health interventions. These findings provide empirical evidence for optimal allocation of global OM prevention and control resources, helping to achieve greater public health impact and health equity progress.

### 4.4 Efficiency frontier analysis: best practice benchmarks at different SDI levels

Efficiency Frontiers analysis revealed capability differences among countries in addressing OM challenges under similar resource conditions. The efficiency boundaries for prevalence and YLD showed smooth declining patterns, indicating that the ability to control long-term disease impacts generally improved with socioeconomic development. However, the “step-wise” distribution of incidence boundaries revealed special relationship patterns between socioeconomic development and disease prevention: regions with SDI values below 0.4, between 0.4–0.6, and above 0.6 each formed relatively stable efficiency platforms, indicating obvious jump-like changes in incidence control efficiency at different development stages, rather than continuous improvement with SDI.

Efficiency differences between countries were significant: high-efficiency countries such as Monaco, Australia, and Singapore demonstrated the effectiveness achievable through optimized health systems, while South Asian countries showed significant efficiency gaps. Particularly noteworthy is the abnormal efficiency distribution in incidence control: low-income countries such as North Korea, Somalia, and Niger approached theoretical optimal levels in incidence control, while some Western European high-income countries performed poorly, showing efficiency gaps inconsistent with resource levels.

These findings indicate that achieving effective OM control depends not only on economic resources but is also closely related to health system organization, environmental conditions, and lifestyle factors. Countries furthest from the efficiency frontier, such as South Asia and sub-Saharan Africa regions, have the potential to achieve significant improvements under existing resource conditions by learning best practices from frontier countries.

### 4.5 Implications for policy and intervention strategies

Based on the comprehensive findings of this study, we propose the following policy recommendations:

1) **Stratified Intervention Strategies**: countries at different SDI levels should have different intervention priorities. Low-SDI regions should prioritize improving basic medical conditions, sanitary facilities, and healthcare accessibility. High-SDI regions need to identify and reduce potential factors causing increased incidence rates, focusing on rational antibiotic use, infection control in collective childcare environments, and allergic disease management, while also considering potential changes in incidence data due to improved diagnostic efficiency.
2) **Strengthening Early Childhood Interventions**: targeting the persistently high burden and even upward trends in the 0–5 age group, strengthen infant and toddler OM prevention and control measures, including promoting breastfeeding, expanding pneumococcal and Haemophilus influenzae vaccine coverage, reducing environmental tobacco exposure, and improving parents' ability to recognize early symptoms. Simultaneously strengthen prevention strategies across the entire life cycle from children to middle-aged adults.
3) **Healthcare System Efficiency Optimization**: learn from the experiences of best-performing countries in efficiency Frontiers analysis to optimize health system structure and operating mechanisms. Particularly for countries with large efficiency gaps, policy innovation and system improvements may achieve health benefits that exceed current socioeconomic levels.
4) **Enhanced Surveillance and Research**: establish more comprehensive, standardized global OM surveillance systems, particularly in Low-SDI regions, providing more accurate disease burden estimates and intervention effectiveness assessments. Conduct in-depth research on specific causes of incidence changes in different regions, exploring the impact of modern lifestyle factors on OM risk.

### 4.6 Study limitations

This study has several limitations that need consideration. First, the GBD database relies on data reported by countries, and data quality and completeness may vary by region, particularly in resource-poor areas. Second, inconsistencies in OM diagnostic criteria across countries may affect the comparability of disease burden estimates. Additionally, our study could not distinguish burden characteristics of different OM subtypes (acute, chronic, otitis media with effusion), which may have significant differences in etiology, clinical presentation, and disease progression.

## 5 Conclusion

This comprehensive assessment of global OM burden revealed several key epidemiological characteristics: global OM showed “dual trends” from 1990 to 2021 (increasing absolute case numbers but decreasing age-standardized rates), while disease burden was strongly negatively correlated with socioeconomic development levels, but High-SDI regions showed “reverse development gradient” phenomena in incidence rates. APC analysis confirmed that early childhood is the peak disease period, and in the highest burden 0–5 age group, disease burden was not only not effectively controlled but even showed upward trends in multiple SDI regions; particularly noteworthy is that Low-SDI regions, despite having the heaviest burden and greatest improvement potential, showed relatively slow improvement rates, possibly related to resource constraints and insufficient health system capacity. Meanwhile, incidence rates in the broad 0–55 age range generally increased, indicating that OM prevention and control faces cross-age challenges. Efficiency Frontier analysis provided optimal prevention and control references for countries at different SDI levels. Based on these findings, our study highlights the importance of focusing on early childhood interventions and implementing SDI-stratified prevention and control measures. Through developing intervention strategies targeting the highest burden but relatively limited improvement 0–5 age group and addressing new challenges of rising incidence rates across broad age ranges, we can expect to achieve maximum reduction in global OM disease burden with minimal investment, promoting universal improvement in global child health.

## Data Availability

The original contributions presented in the study are included in the article/Supplementary material, further inquiries can be directed to the corresponding authors.
