# Supplementary material for: Systematic analysis of global trends in otitis media burden across Socio-Demographic Index levels, 1990–2021: findings from the global burden of disease study 2021
Source: Front Public Health. 2026 Mar 12;14:1694807. doi: 10.3389/fpubh.2026.1694807 (PMC13017811; doi:10.3389/fpubh.2026.1694807)
Supplement: Supplementary file 1 [file Data_Sheet_1.docx]

[**Table S1. ASRs of Prevalence in 204 Countries and Territories, 2021** 1](#_Toc207145008)

[**Table S2. ASR of Incidence in 204 Countries and Territories, 2021** 10](#_Toc207145009)

[**Table S3. ASR of YLDs in 204 Countries and Territories, 2021** 18](#_Toc207145010)

[**Table S4. Net Drift values (percent change per year) for prevalence, incidence, and YLDS of OM by SDI level, 1990-2021.** 27](#_Toc207145011)

[**Table S5. Statistical tests for Age-Period-Cohort effects for prevalence, incidence, and YLDs of OM by SDI level, 1990-2021.** 28](#_Toc207145012)

[**Table S6. Estimated parameters from Age-Period-Cohort analysis for prevalence, incidence, and YLDs of OM by SDI level, 1990-2021.** 35](#_Toc207145013)

[**Table S7. Age-Standardized Rates and Frontier Analysis Results for Otitis Media by Country, 2021** 75](#_Toc207145014)

| **Table S1. ASRs of Prevalence in 204 Countries and Territories, 2021** | | |
| --- | --- | --- |
| **Rank** | **Country** | **Prevalence per 100,000 (95% UI)** |
| 1 | Somalia | 2925.50 (2451.33, 3494.39) |
| 2 | Niger | 2381.99 (1981.32, 2822.83) |
| 3 | Liberia | 2349.97 (1968.25, 2776.01) |
| 4 | Nepal | 2291.21 (1943.20, 2668.48) |
| 5 | Central African Republic | 2290.43 (1920.94, 2703.44) |
| 6 | Democratic Republic of the Congo | 2235.34 (1882.69, 2627.60) |
| 7 | Sierra Leone | 2217.02 (1855.29, 2602.84) |
| 8 | Togo | 2166.25 (1822.75, 2537.17) |
| 9 | Burundi | 2156.93 (1824.41, 2509.80) |
| 10 | Bangladesh | 2154.90 (1844.67, 2504.12) |
| 11 | Guinea-Bissau | 2150.86 (1800.22, 2527.02) |
| 12 | Burkina Faso | 2129.23 (1801.71, 2515.36) |
| 13 | Gambia | 2094.95 (1760.36, 2448.46) |
| 14 | Pakistan | 2087.68 (1792.70, 2406.60) |
| 15 | Chad | 2082.74 (1757.37, 2459.82) |
| 16 | Mali | 2060.89 (1741.00, 2415.91) |
| 17 | Afghanistan | 2049.88 (1729.33, 2393.61) |
| 18 | Guinea | 2049.83 (1732.52, 2390.89) |
| 19 | India | 2026.90 (1739.06, 2344.54) |
| 20 | Malawi | 2017.32 (1721.41, 2352.09) |
| 21 | Benin | 2006.68 (1703.59, 2355.48) |
| 22 | Haiti | 1982.73 (1684.37, 2308.43) |
| 23 | Yemen | 1977.95 (1650.09, 2318.39) |
| 24 | Zimbabwe | 1945.91 (1654.28, 2266.36) |
| 25 | Cameroon | 1938.84 (1641.18, 2258.12) |
| 26 | Mozambique | 1934.13 (1630.10, 2260.86) |
| 27 | Ethiopia | 1931.58 (1665.72, 2238.22) |
| 28 | Senegal | 1917.03 (1619.84, 2244.90) |
| 29 | Madagascar | 1905.12 (1612.94, 2247.18) |
| 30 | Sao Tome and Principe | 1899.32 (1623.27, 2207.83) |
| 31 | Mauritania | 1865.05 (1572.14, 2174.12) |
| 32 | Côte d'Ivoire | 1862.64 (1574.35, 2182.82) |
| 33 | Lesotho | 1853.01 (1591.20, 2130.49) |
| 34 | Bhutan | 1847.01 (1584.19, 2137.67) |
| 35 | Eritrea | 1830.31 (1553.47, 2147.68) |
| 36 | Nigeria | 1800.74 (1538.35, 2085.54) |
| 37 | Sudan | 1788.52 (1513.95, 2081.70) |
| 38 | Democratic People's Republic of Korea | 1776.59 (1492.33, 2082.81) |
| 39 | Ghana | 1771.19 (1507.59, 2064.72) |
| 40 | Kiribati | 1769.24 (1510.29, 2056.45) |
| 41 | South Sudan | 1763.30 (1490.32, 2091.95) |
| 42 | Palestine | 1759.28 (1505.65, 2060.91) |
| 43 | Uganda | 1757.56 (1489.54, 2046.73) |
| 44 | Syrian Arab Republic | 1756.12 (1508.65, 2045.56) |
| 45 | Rwanda | 1753.00 (1484.61, 2040.58) |
| 46 | Solomon Islands | 1748.80 (1486.13, 2028.15) |
| 47 | Kenya | 1734.22 (1492.62, 2008.92) |
| 48 | Comoros | 1720.68 (1453.31, 2010.49) |
| 49 | Tajikistan | 1700.45 (1437.36, 2004.62) |
| 50 | Honduras | 1694.04 (1460.18, 1951.27) |
| 51 | Cabo Verde | 1682.24 (1430.06, 1968.19) |
| 52 | Nicaragua | 1680.03 (1456.70, 1949.10) |
| 53 | Vanuatu | 1672.21 (1426.20, 1954.48) |
| 54 | United Republic of Tanzania | 1671.96 (1424.55, 1950.93) |
| 55 | Micronesia (Federated States of) | 1629.96 (1381.81, 1904.82) |
| 56 | Kyrgyzstan | 1620.30 (1368.67, 1890.51) |
| 57 | South Africa | 1613.32 (1410.54, 1865.96) |
| 58 | Cambodia | 1609.91 (1365.42, 1889.62) |
| 59 | Marshall Islands | 1609.38 (1370.51, 1877.35) |
| 60 | Djibouti | 1606.34 (1371.56, 1875.48) |
| 61 | Zambia | 1605.23 (1355.99, 1889.81) |
| 62 | Congo | 1603.37 (1370.52, 1880.68) |
| 63 | Republic of Moldova | 1599.92 (1364.52, 1865.61) |
| 64 | Tuvalu | 1596.25 (1353.80, 1846.29) |
| 65 | Papua New Guinea | 1586.14 (1346.80, 1843.32) |
| 66 | Morocco | 1586.08 (1369.85, 1847.78) |
| 67 | Eswatini | 1574.59 (1350.82, 1825.00) |
| 68 | Ukraine | 1573.16 (1358.86, 1808.26) |
| 69 | Guatemala | 1573.00 (1354.41, 1821.70) |
| 70 | El Salvador | 1565.65 (1347.04, 1817.74) |
| 71 | Iran (Islamic Republic of) | 1554.33 (1348.29, 1790.90) |
| 72 | Jordan | 1549.73 (1333.73, 1803.62) |
| 73 | Angola | 1542.89 (1325.91, 1787.89) |
| 74 | Bolivia (Plurinational State of) | 1540.76 (1322.49, 1774.84) |
| 75 | Namibia | 1527.42 (1327.19, 1778.38) |
| 76 | Egypt | 1524.73 (1313.75, 1769.11) |
| 77 | Philippines | 1522.47 (1318.53, 1763.81) |
| 78 | Jamaica | 1521.32 (1311.56, 1762.53) |
| 79 | Cuba | 1519.19 (1300.04, 1763.14) |
| 80 | Belize | 1517.19 (1303.47, 1752.70) |
| 81 | Tunisia | 1500.33 (1283.78, 1744.28) |
| 82 | Guyana | 1497.62 (1298.47, 1739.26) |
| 83 | Myanmar | 1486.40 (1258.92, 1740.54) |
| 84 | Timor-Leste | 1481.65 (1255.97, 1725.32) |
| 85 | Libya | 1478.98 (1261.53, 1724.55) |
| 86 | Venezuela (Bolivarian Republic of) | 1471.93 (1270.04, 1709.25) |
| 87 | Mexico | 1471.13 (1284.65, 1689.46) |
| 88 | Tonga | 1470.99 (1258.30, 1702.99) |
| 89 | Samoa | 1470.84 (1259.06, 1720.26) |
| 90 | Dominica | 1469.01 (1262.57, 1706.79) |
| 91 | Algeria | 1460.82 (1259.33, 1697.49) |
| 92 | Lebanon | 1458.59 (1259.16, 1696.40) |
| 93 | Indonesia | 1456.77 (1261.02, 1689.92) |
| 94 | Saint Vincent and the Grenadines | 1455.45 (1249.02, 1691.00) |
| 95 | Lao People's Democratic Republic | 1453.49 (1235.95, 1695.40) |
| 96 | Ecuador | 1444.61 (1255.12, 1677.27) |
| 97 | Colombia | 1438.16 (1247.85, 1652.49) |
| 98 | Botswana | 1438.10 (1241.07, 1669.44) |
| 99 | Uzbekistan | 1437.64 (1230.25, 1670.24) |
| 100 | Iraq | 1427.60 (1228.76, 1662.28) |
| 101 | Brazil | 1424.96 (1241.36, 1658.30) |
| 102 | Saint Lucia | 1424.43 (1228.46, 1645.10) |
| 103 | Tokelau | 1421.80 (1220.03, 1656.20) |
| 104 | Costa Rica | 1408.68 (1220.79, 1639.97) |
| 105 | Armenia | 1408.36 (1203.65, 1636.92) |
| 106 | Viet Nam | 1406.07 (1187.37, 1634.61) |
| 107 | Grenada | 1400.25 (1208.68, 1611.52) |
| 108 | Suriname | 1393.46 (1204.83, 1603.12) |
| 109 | Paraguay | 1390.19 (1203.85, 1612.65) |
| 110 | Peru | 1385.77 (1194.64, 1605.19) |
| 111 | Barbados | 1383.83 (1192.87, 1603.57) |
| 112 | Georgia | 1380.87 (1188.02, 1610.96) |
| 113 | Russian Federation | 1373.88 (1203.74, 1581.06) |
| 114 | Dominican Republic | 1373.47 (1189.13, 1588.42) |
| 115 | Mongolia | 1349.05 (1159.82, 1576.76) |
| 116 | Fiji | 1348.97 (1157.07, 1567.48) |
| 117 | Albania | 1346.31 (1149.54, 1572.46) |
| 118 | Belarus | 1344.67 (1165.09, 1552.15) |
| 119 | Gabon | 1344.30 (1151.70, 1570.52) |
| 120 | Bosnia and Herzegovina | 1343.23 (1145.83, 1575.56) |
| 121 | China | 1332.11 (1158.46, 1541.88) |
| 122 | Panama | 1331.43 (1149.59, 1546.85) |
| 123 | Türkiye | 1322.89 (1127.74, 1531.16) |
| 124 | Sri Lanka | 1315.56 (1121.95, 1547.14) |
| 125 | Nauru | 1310.32 (1124.85, 1527.21) |
| 126 | North Macedonia | 1309.56 (1109.39, 1515.87) |
| 127 | Niue | 1296.89 (1106.68, 1515.33) |
| 128 | Antigua and Barbuda | 1292.44 (1111.09, 1493.24) |
| 129 | Thailand | 1290.72 (1105.80, 1501.18) |
| 130 | Azerbaijan | 1288.93 (1108.42, 1502.46) |
| 131 | Serbia | 1284.78 (1101.51, 1494.94) |
| 132 | Latvia | 1273.79 (1102.53, 1485.10) |
| 133 | American Samoa | 1265.66 (1086.26, 1467.51) |
| 134 | Equatorial Guinea | 1264.71 (1083.92, 1473.70) |
| 135 | Saint Kitts and Nevis | 1262.40 (1092.11, 1473.40) |
| 136 | Montenegro | 1260.76 (1085.80, 1476.36) |
| 137 | Maldives | 1256.23 (1074.54, 1480.02) |
| 138 | Palau | 1253.01 (1080.17, 1466.61) |
| 139 | Estonia | 1246.21 (1083.44, 1445.42) |
| 140 | Lithuania | 1245.89 (1082.15, 1452.82) |
| 141 | Bahamas | 1244.23 (1073.14, 1437.85) |
| 142 | Turkmenistan | 1242.97 (1066.65, 1457.49) |
| 143 | Bulgaria | 1231.33 (1056.00, 1428.84) |
| 144 | Bahrain | 1230.59 (1057.53, 1441.89) |
| 145 | Trinidad and Tobago | 1224.60 (1052.16, 1424.09) |
| 146 | Poland | 1219.33 (1053.40, 1415.40) |
| 147 | Puerto Rico | 1212.39 (1043.29, 1401.78) |
| 148 | Saudi Arabia | 1209.73 (1042.86, 1417.77) |
| 149 | Kazakhstan | 1204.07 (1035.27, 1425.46) |
| 150 | Croatia | 1194.94 (1015.73, 1390.82) |
| 151 | Mauritius | 1194.45 (1014.00, 1397.62) |
| 152 | Romania | 1194.44 (1023.92, 1391.77) |
| 153 | Oman | 1193.48 (1022.43, 1388.53) |
| 154 | Hungary | 1179.02 (1001.09, 1370.40) |
| 155 | Kuwait | 1162.55 (1006.99, 1352.86) |
| 156 | United States Virgin Islands | 1161.14 (1002.96, 1353.01) |
| 157 | United Arab Emirates | 1155.90 (991.70, 1341.94) |
| 158 | Malaysia | 1153.84 (983.17, 1365.49) |
| 159 | Seychelles | 1152.54 (980.29, 1356.56) |
| 160 | Slovakia | 1146.47 (981.77, 1344.39) |
| 161 | Spain | 1132.60 (965.48, 1306.83) |
| 162 | Slovenia | 1130.52 (969.33, 1320.85) |
| 163 | Czechia | 1125.33 (962.84, 1325.41) |
| 164 | Bermuda | 1121.86 (967.77, 1300.38) |
| 165 | Greece | 1117.97 (947.32, 1297.36) |
| 166 | Northern Mariana Islands | 1109.70 (950.20, 1298.99) |
| 167 | Cook Islands | 1104.51 (948.51, 1291.40) |
| 168 | Qatar | 1101.36 (942.32, 1280.98) |
| 169 | Argentina | 1092.88 (942.54, 1266.25) |
| 170 | United Kingdom | 1089.90 (937.28, 1261.26) |
| 171 | Sweden | 1077.93 (920.34, 1244.61) |
| 172 | France | 1072.71 (918.71, 1240.86) |
| 173 | Israel | 1071.87 (903.97, 1245.89) |
| 174 | Norway | 1070.05 (920.01, 1241.67) |
| 175 | Uruguay | 1067.27 (918.52, 1242.85) |
| 176 | Cyprus | 1058.66 (899.45, 1227.79) |
| 177 | Italy | 1054.79 (914.72, 1219.53) |
| 178 | Chile | 1053.90 (910.25, 1214.04) |
| 179 | Portugal | 1053.04 (893.26, 1234.96) |
| 180 | Malta | 1041.61 (889.77, 1218.68) |
| 181 | Japan | 1035.90 (901.36, 1197.60) |
| 182 | Guam | 1033.13 (880.15, 1206.48) |
| 183 | Belgium | 1024.49 (867.47, 1197.57) |
| 184 | Iceland | 1001.39 (853.08, 1164.49) |
| 185 | Denmark | 996.39 (845.23, 1158.79) |
| 186 | Finland | 995.21 (843.85, 1153.58) |
| 187 | Netherlands | 990.76 (836.66, 1153.53) |
| 188 | Germany | 988.92 (847.30, 1153.20) |
| 189 | San Marino | 978.36 (831.40, 1141.40) |
| 190 | Taiwan (Province of China) | 969.33 (835.97, 1131.44) |
| 191 | Republic of Korea | 958.09 (819.44, 1109.86) |
| 192 | Switzerland | 956.80 (821.37, 1108.07) |
| 193 | Ireland | 953.94 (809.97, 1104.11) |
| 194 | Andorra | 941.91 (804.02, 1097.49) |
| 195 | Austria | 940.02 (801.66, 1104.57) |
| 196 | United States of America | 934.80 (821.50, 1051.57) |
| 197 | Canada | 921.95 (795.89, 1062.84) |
| 198 | Luxembourg | 905.14 (766.07, 1051.40) |
| 199 | New Zealand | 898.34 (764.94, 1039.42) |
| 200 | Brunei Darussalam | 876.74 (755.24, 1025.27) |
| 201 | Greenland | 852.31 (735.07, 982.86) |
| 202 | Singapore | 851.70 (733.24, 997.27) |
| 203 | Australia | 793.21 (684.78, 919.06) |
| 204 | Monaco | 757.70 (637.13, 889.70) |

| **Table S2. ASR of Incidence in 204 Countries and Territories, 2021** | | |
| --- | --- | --- |
| **Rank** | **Country** | **Incidence per 100,000 (95% UI)** |
| 1 | Pakistan | 6768.00 (4938.04, 9218.64) |
| 2 | Spain | 6702.59 (5333.10, 8584.72) |
| 3 | Ethiopia | 6639.68 (4856.82, 9029.70) |
| 4 | Kenya | 6637.70 (4856.39, 9046.74) |
| 5 | India | 6624.71 (4829.40, 9046.91) |
| 6 | Malawi | 6382.92 (4664.69, 8435.31) |
| 7 | Nigeria | 6342.18 (4681.61, 8634.91) |
| 8 | South Africa | 6335.66 (4684.71, 8634.99) |
| 9 | Nepal | 6234.13 (4565.30, 8488.51) |
| 10 | Bhutan | 6229.64 (4559.06, 8486.67) |
| 11 | Bangladesh | 6223.39 (4533.86, 8478.82) |
| 12 | Djibouti | 6140.04 (4479.00, 8339.01) |
| 13 | South Sudan | 6135.14 (4478.40, 8343.44) |
| 14 | Somalia | 6134.46 (4480.29, 8345.92) |
| 15 | Eritrea | 6132.10 (4478.98, 8344.73) |
| 16 | Uganda | 6131.84 (4478.96, 8345.12) |
| 17 | Rwanda | 6127.45 (4477.33, 8345.37) |
| 18 | Comoros | 6127.37 (4475.56, 8342.38) |
| 19 | Mozambique | 6125.02 (4475.38, 8344.35) |
| 20 | United Republic of Tanzania | 6124.74 (4474.36, 8341.92) |
| 21 | Madagascar | 6124.00 (4473.84, 8341.96) |
| 22 | Zambia | 6121.82 (4472.26, 8339.30) |
| 23 | Burundi | 6117.02 (4468.49, 8335.98) |
| 24 | Eswatini | 5818.45 (4292.14, 7934.53) |
| 25 | Zimbabwe | 5817.60 (4290.56, 7937.25) |
| 26 | Ghana | 5817.18 (4291.55, 7935.24) |
| 27 | Guinea | 5817.14 (4291.11, 7936.64) |
| 28 | Guinea-Bissau | 5817.06 (4291.33, 7935.91) |
| 29 | Togo | 5816.87 (4290.48, 7933.67) |
| 30 | Burkina Faso | 5816.69 (4289.61, 7937.39) |
| 31 | Lesotho | 5816.64 (4290.26, 7935.15) |
| 32 | Central African Republic | 5816.31 (4289.70, 7935.66) |
| 33 | Angola | 5816.04 (4289.24, 7936.83) |
| 34 | Namibia | 5816.00 (4289.54, 7935.86) |
| 35 | Benin | 5815.72 (4289.81, 7935.21) |
| 36 | Mauritania | 5815.54 (4289.59, 7935.26) |
| 37 | Niger | 5815.46 (4289.93, 7934.62) |
| 38 | Equatorial Guinea | 5814.86 (4292.73, 7920.87) |
| 39 | Gambia | 5814.83 (4288.39, 7935.00) |
| 40 | Gabon | 5814.75 (4287.80, 7938.25) |
| 41 | Botswana | 5814.62 (4288.01, 7933.32) |
| 42 | Senegal | 5814.48 (4288.92, 7930.78) |
| 43 | Cameroon | 5814.22 (4288.08, 7932.88) |
| 44 | Mali | 5814.10 (4287.46, 7933.20) |
| 45 | Congo | 5813.61 (4286.53, 7933.45) |
| 46 | Chad | 5813.42 (4287.12, 7933.59) |
| 47 | Democratic Republic of the Congo | 5813.21 (4287.02, 7931.60) |
| 48 | Sierra Leone | 5812.75 (4285.67, 7933.44) |
| 49 | Liberia | 5811.76 (4285.42, 7930.79) |
| 50 | Côte d'Ivoire | 5810.87 (4283.86, 7927.74) |
| 51 | Sao Tome and Principe | 5807.63 (4284.52, 7923.06) |
| 52 | Cabo Verde | 5807.24 (4284.17, 7922.61) |
| 53 | Norway | 5791.00 (4278.90, 7799.98) |
| 54 | Sweden | 5757.84 (4265.54, 7787.34) |
| 55 | United Kingdom | 5721.19 (4264.90, 7871.17) |
| 56 | Mexico | 5680.67 (4255.79, 7693.08) |
| 57 | Brazil | 5675.50 (4254.30, 7688.30) |
| 58 | Iran (Islamic Republic of) | 5669.87 (4269.29, 7692.03) |
| 59 | New Zealand | 5409.01 (3997.57, 7307.44) |
| 60 | Ireland | 5325.88 (3943.71, 7274.25) |
| 61 | San Marino | 5323.54 (3962.12, 7226.01) |
| 62 | Netherlands | 5321.18 (3932.30, 7271.76) |
| 63 | Israel | 5320.62 (3959.86, 7223.99) |
| 64 | Monaco | 5319.57 (3868.60, 7328.32) |
| 65 | Portugal | 5319.37 (3888.87, 7323.65) |
| 66 | Belgium | 5317.95 (3927.90, 7306.36) |
| 67 | Cyprus | 5317.09 (3943.97, 7267.24) |
| 68 | Andorra | 5316.10 (3930.54, 7260.46) |
| 69 | Denmark | 5313.03 (3944.22, 7136.87) |
| 70 | Austria | 5313.02 (3923.56, 7299.80) |
| 71 | Switzerland | 5311.00 (3942.83, 7134.41) |
| 72 | Germany | 5308.88 (3942.51, 7359.14) |
| 73 | France | 5307.65 (3965.85, 7193.25) |
| 74 | Greece | 5298.96 (3902.75, 7121.72) |
| 75 | Luxembourg | 5298.68 (3947.18, 7066.68) |
| 76 | Finland | 5286.38 (3916.26, 7200.57) |
| 77 | Malta | 5285.12 (3878.69, 7191.05) |
| 78 | Iceland | 5284.51 (3914.38, 7198.52) |
| 79 | Barbados | 5189.95 (3835.89, 7084.38) |
| 80 | Syrian Arab Republic | 5185.87 (3853.43, 7121.43) |
| 81 | Puerto Rico | 5185.72 (3899.52, 7094.75) |
| 82 | Bermuda | 5185.14 (3887.75, 7005.23) |
| 83 | Antigua and Barbuda | 5183.79 (3841.34, 6999.78) |
| 84 | Bahamas | 5183.66 (3859.23, 6917.75) |
| 85 | United States Virgin Islands | 5183.57 (3825.91, 7029.45) |
| 86 | Costa Rica | 5182.29 (3849.89, 6967.06) |
| 87 | Ecuador | 5181.31 (3848.34, 7115.61) |
| 88 | Guatemala | 5180.99 (3884.55, 6936.98) |
| 89 | Bolivia (Plurinational State of) | 5180.99 (3848.03, 7115.38) |
| 90 | Haiti | 5180.09 (3883.47, 6936.60) |
| 91 | Honduras | 5179.22 (3883.63, 6934.53) |
| 92 | El Salvador | 5179.12 (3883.85, 6933.59) |
| 93 | Yemen | 5179.03 (3846.78, 7112.69) |
| 94 | Morocco | 5178.89 (3846.51, 7112.75) |
| 95 | Belize | 5178.73 (3882.06, 6935.67) |
| 96 | Türkiye | 5178.35 (3845.92, 7112.08) |
| 97 | Jamaica | 5178.32 (3820.46, 7109.32) |
| 98 | Palestine | 5178.21 (3845.88, 7112.22) |
| 99 | Saint Lucia | 5177.82 (3837.69, 6996.19) |
| 100 | Paraguay | 5177.72 (3845.65, 7111.66) |
| 101 | Libya | 5177.49 (3845.06, 7111.61) |
| 102 | Guyana | 5177.13 (3881.48, 6934.13) |
| 103 | Algeria | 5177.04 (3844.80, 7110.83) |
| 104 | Venezuela (Bolivarian Republic of) | 5176.92 (3859.25, 7100.65) |
| 105 | Afghanistan | 5176.80 (3846.19, 7108.79) |
| 106 | Peru | 5176.30 (3844.68, 7107.83) |
| 107 | Trinidad and Tobago | 5176.30 (3818.89, 7107.41) |
| 108 | Sudan | 5176.26 (3844.44, 7109.68) |
| 109 | Tunisia | 5176.17 (3845.13, 7108.58) |
| 110 | Colombia | 5176.11 (3881.56, 6931.73) |
| 111 | Nicaragua | 5176.10 (3881.72, 6931.28) |
| 112 | Suriname | 5175.73 (3881.25, 6930.04) |
| 113 | Cuba | 5175.52 (3844.35, 6961.70) |
| 114 | Dominican Republic | 5175.45 (3880.16, 6931.95) |
| 115 | Iraq | 5175.41 (3843.61, 7108.92) |
| 116 | Saint Kitts and Nevis | 5175.07 (3845.35, 7015.90) |
| 117 | Egypt | 5174.93 (3842.86, 7107.65) |
| 118 | Saint Vincent and the Grenadines | 5172.97 (3854.61, 7095.60) |
| 119 | Panama | 5172.84 (3879.21, 6929.33) |
| 120 | Dominica | 5172.19 (3843.17, 7011.61) |
| 121 | Jordan | 5171.76 (3839.67, 7105.68) |
| 122 | Grenada | 5170.19 (3853.48, 7092.92) |
| 123 | Lebanon | 5169.79 (3864.60, 7099.97) |
| 124 | Kuwait | 5166.63 (3797.24, 7001.16) |
| 125 | Oman | 5164.08 (3831.92, 7099.92) |
| 126 | Saudi Arabia | 5162.13 (3831.75, 7093.65) |
| 127 | Bahrain | 5154.71 (3846.37, 7082.42) |
| 128 | United Arab Emirates | 5148.68 (3839.40, 7078.71) |
| 129 | Qatar | 5140.43 (3809.87, 6941.95) |
| 130 | Chile | 4970.13 (3674.57, 6677.07) |
| 131 | Australia | 4967.12 (3695.37, 6548.29) |
| 132 | Uruguay | 4956.19 (3673.81, 6570.86) |
| 133 | Argentina | 4955.49 (3673.46, 6570.87) |
| 134 | Indonesia | 4821.53 (3554.80, 6569.79) |
| 135 | Philippines | 4820.53 (3555.68, 6568.34) |
| 136 | Ukraine | 4774.91 (3601.34, 6560.83) |
| 137 | Russian Federation | 4765.67 (3574.77, 6488.43) |
| 138 | Kiribati | 4609.04 (3384.25, 6324.09) |
| 139 | Fiji | 4605.60 (3378.83, 6321.05) |
| 140 | Vanuatu | 4605.38 (3379.16, 6320.10) |
| 141 | Marshall Islands | 4604.86 (3378.12, 6320.41) |
| 142 | Micronesia (Federated States of) | 4604.85 (3377.88, 6319.98) |
| 143 | Nauru | 4604.14 (3379.46, 6318.12) |
| 144 | Solomon Islands | 4602.80 (3376.89, 6316.58) |
| 145 | Tonga | 4602.46 (3389.51, 6233.25) |
| 146 | American Samoa | 4602.35 (3405.87, 6272.18) |
| 147 | Niue | 4601.97 (3405.80, 6273.24) |
| 148 | Tokelau | 4600.96 (3374.72, 6314.82) |
| 149 | Samoa | 4600.67 (3387.38, 6230.92) |
| 150 | Papua New Guinea | 4600.05 (3373.66, 6313.27) |
| 151 | Cook Islands | 4598.96 (3428.60, 6229.27) |
| 152 | Tuvalu | 4598.45 (3372.03, 6311.30) |
| 153 | Northern Mariana Islands | 4594.03 (3365.33, 6254.17) |
| 154 | Guam | 4593.87 (3422.45, 6223.03) |
| 155 | Palau | 4592.81 (3380.34, 6224.27) |
| 156 | Myanmar | 4457.19 (3241.87, 6140.80) |
| 157 | Sri Lanka | 4456.87 (3241.61, 6140.83) |
| 158 | Cambodia | 4455.89 (3241.40, 6139.61) |
| 159 | Thailand | 4454.81 (3240.11, 6139.51) |
| 160 | Mauritius | 4454.22 (3257.31, 6064.74) |
| 161 | Lao People's Democratic Republic | 4454.10 (3239.77, 6138.66) |
| 162 | Timor-Leste | 4452.35 (3238.18, 6138.52) |
| 163 | Viet Nam | 4451.64 (3238.42, 6136.29) |
| 164 | Malaysia | 4450.04 (3237.08, 6134.75) |
| 165 | Seychelles | 4449.53 (3237.40, 6133.04) |
| 166 | Maldives | 4437.80 (3228.82, 6121.72) |
| 167 | Japan | 4399.71 (3334.34, 5746.55) |
| 168 | Canada | 4384.76 (3249.50, 5879.23) |
| 169 | Mongolia | 4383.65 (3285.29, 5965.72) |
| 170 | Kazakhstan | 4382.11 (3251.12, 5968.65) |
| 171 | Lithuania | 4381.84 (3272.98, 5943.30) |
| 172 | Uzbekistan | 4381.58 (3282.82, 5964.03) |
| 173 | Republic of Moldova | 4381.34 (3283.06, 5963.76) |
| 174 | Armenia | 4381.23 (3263.40, 5992.35) |
| 175 | Kyrgyzstan | 4380.23 (3249.34, 5966.70) |
| 176 | Azerbaijan | 4380.12 (3281.17, 5965.69) |
| 177 | Belarus | 4379.76 (3249.07, 5965.92) |
| 178 | Latvia | 4378.85 (3247.75, 5965.22) |
| 179 | Tajikistan | 4378.74 (3279.87, 5960.29) |
| 180 | Estonia | 4378.04 (3269.39, 5939.09) |
| 181 | Georgia | 4376.00 (3258.33, 5987.33) |
| 182 | Greenland | 4375.20 (3233.50, 5825.76) |
| 183 | Turkmenistan | 4372.83 (3274.35, 5954.80) |
| 184 | Italy | 4306.40 (3226.70, 5697.30) |
| 185 | China | 4179.92 (3197.52, 5614.57) |
| 186 | United States of America | 4168.22 (3571.78, 4848.98) |
| 187 | Republic of Korea | 4013.07 (3074.09, 5246.48) |
| 188 | Singapore | 4011.36 (3019.38, 5226.93) |
| 189 | Montenegro | 4008.41 (2930.89, 5569.64) |
| 190 | Croatia | 4007.54 (2930.19, 5566.18) |
| 191 | Slovakia | 4006.95 (2929.61, 5564.92) |
| 192 | Brunei Darussalam | 4004.78 (3066.20, 5234.21) |
| 193 | Albania | 4004.16 (2944.00, 5472.74) |
| 194 | Romania | 4003.85 (2944.92, 5474.47) |
| 195 | Bulgaria | 4003.32 (2944.23, 5473.78) |
| 196 | Czechia | 3999.58 (2914.91, 5476.62) |
| 197 | Serbia | 3999.37 (2897.96, 5519.84) |
| 198 | North Macedonia | 3998.87 (2898.58, 5525.47) |
| 199 | Slovenia | 3997.89 (2913.32, 5474.60) |
| 200 | Bosnia and Herzegovina | 3996.96 (2958.84, 5519.46) |
| 201 | Hungary | 3993.23 (2949.07, 5459.54) |
| 202 | Democratic People's Republic of Korea | 3788.21 (2872.24, 5079.51) |
| 203 | Poland | 3767.26 (3089.27, 4564.42) |
| 204 | Taiwan (Province of China) | 3220.69 (2405.95, 4330.49) |

| **Table S3. ASR of YLDs in 204 Countries and Territories, 2021** | | |
| --- | --- | --- |
| **Rank** | **Country** | **YLDs per 100,000 (95% UI)** |
| 1 | Somalia | 60.21 (35.73, 94.52) |
| 2 | Niger | 50.17 (29.32, 80.53) |
| 3 | Liberia | 49.07 (28.83, 78.26) |
| 4 | Central African Republic | 47.01 (27.80, 74.09) |
| 5 | Nepal | 46.74 (27.36, 75.13) |
| 6 | Sierra Leone | 46.33 (27.46, 74.07) |
| 7 | Democratic Republic of the Congo | 45.90 (26.64, 73.89) |
| 8 | Togo | 45.32 (26.92, 71.51) |
| 9 | Guinea-Bissau | 44.91 (26.27, 71.77) |
| 10 | Burkina Faso | 44.53 (26.23, 71.12) |
| 11 | Bangladesh | 43.87 (25.43, 70.22) |
| 12 | Burundi | 43.82 (25.52, 70.37) |
| 13 | Gambia | 43.72 (25.31, 70.66) |
| 14 | Chad | 43.42 (25.71, 70.06) |
| 15 | Mali | 42.91 (25.16, 68.57) |
| 16 | Guinea | 42.73 (25.58, 68.85) |
| 17 | Afghanistan | 41.97 (24.79, 67.00) |
| 18 | Pakistan | 41.96 (24.60, 67.71) |
| 19 | Benin | 41.74 (24.53, 67.82) |
| 20 | India | 41.49 (24.17, 66.81) |
| 21 | Yemen | 40.46 (23.80, 64.08) |
| 22 | Cameroon | 40.31 (23.72, 64.77) |
| 23 | Malawi | 40.11 (22.97, 64.29) |
| 24 | Senegal | 39.75 (23.32, 63.44) |
| 25 | Haiti | 39.61 (23.14, 63.27) |
| 26 | Sao Tome and Principe | 39.51 (22.88, 63.05) |
| 27 | Ethiopia | 38.90 (22.79, 62.14) |
| 28 | Madagascar | 38.82 (22.73, 62.92) |
| 29 | Mauritania | 38.73 (22.86, 63.07) |
| 30 | Mozambique | 38.70 (22.88, 61.55) |
| 31 | Côte d'Ivoire | 38.54 (22.79, 61.76) |
| 32 | Zimbabwe | 38.44 (22.03, 61.69) |
| 33 | Bhutan | 37.21 (21.91, 59.95) |
| 34 | Nigeria | 37.10 (21.68, 59.88) |
| 35 | Eritrea | 36.70 (21.20, 59.10) |
| 36 | Lesotho | 36.57 (21.41, 58.23) |
| 37 | Ghana | 36.55 (21.05, 58.69) |
| 38 | Sudan | 36.51 (21.27, 58.10) |
| 39 | Palestine | 35.84 (20.71, 57.10) |
| 40 | Syrian Arab Republic | 35.73 (20.79, 57.27) |
| 41 | Kiribati | 35.50 (20.81, 57.77) |
| 42 | Democratic People's Republic of Korea | 35.39 (20.73, 56.41) |
| 43 | Solomon Islands | 35.14 (20.50, 57.24) |
| 44 | Kenya | 35.13 (20.67, 56.44) |
| 45 | South Sudan | 35.09 (20.27, 56.14) |
| 46 | Rwanda | 34.97 (20.55, 57.08) |
| 47 | Tajikistan | 34.66 (20.17, 55.18) |
| 48 | Cabo Verde | 34.66 (20.20, 57.03) |
| 49 | Uganda | 34.63 (20.13, 56.05) |
| 50 | Comoros | 34.38 (19.76, 55.86) |
| 51 | Honduras | 33.62 (20.07, 53.83) |
| 52 | Vanuatu | 33.49 (19.92, 55.13) |
| 53 | Nicaragua | 33.31 (19.67, 53.61) |
| 54 | Kyrgyzstan | 32.96 (19.34, 54.05) |
| 55 | Cambodia | 32.92 (19.21, 53.88) |
| 56 | United Republic of Tanzania | 32.79 (19.12, 53.38) |
| 57 | Micronesia (Federated States of) | 32.61 (19.26, 52.60) |
| 58 | Republic of Moldova | 32.56 (19.22, 52.49) |
| 59 | Congo | 32.20 (18.89, 51.16) |
| 60 | Morocco | 32.09 (18.59, 51.85) |
| 61 | Marshall Islands | 32.07 (18.77, 51.63) |
| 62 | Ukraine | 31.95 (18.77, 50.84) |
| 63 | Tuvalu | 31.92 (18.62, 51.46) |
| 64 | Djibouti | 31.91 (18.25, 51.45) |
| 65 | Zambia | 31.69 (18.47, 50.75) |
| 66 | Papua New Guinea | 31.63 (18.29, 51.34) |
| 67 | South Africa | 31.63 (18.58, 50.58) |
| 68 | Jordan | 31.32 (18.21, 50.38) |
| 69 | Iran (Islamic Republic of) | 31.11 (18.08, 49.91) |
| 70 | Guatemala | 31.00 (18.32, 48.98) |
| 71 | Philippines | 30.99 (18.02, 50.16) |
| 72 | El Salvador | 30.92 (17.82, 49.27) |
| 73 | Angola | 30.91 (18.04, 50.11) |
| 74 | Egypt | 30.90 (18.05, 48.04) |
| 75 | Eswatini | 30.72 (17.78, 49.41) |
| 76 | Myanmar | 30.35 (17.39, 48.85) |
| 77 | Tunisia | 30.21 (17.70, 48.59) |
| 78 | Timor-Leste | 30.11 (17.49, 48.81) |
| 79 | Cuba | 30.06 (17.38, 48.13) |
| 80 | Jamaica | 30.06 (17.35, 48.33) |
| 81 | Belize | 29.97 (17.44, 48.15) |
| 82 | Bolivia (Plurinational State of) | 29.93 (17.85, 47.94) |
| 83 | Namibia | 29.86 (17.38, 47.60) |
| 84 | Libya | 29.75 (17.52, 47.77) |
| 85 | Lao People's Democratic Republic | 29.52 (17.19, 47.86) |
| 86 | Algeria | 29.46 (17.17, 47.11) |
| 87 | Guyana | 29.40 (17.33, 46.70) |
| 88 | Indonesia | 29.38 (17.08, 47.62) |
| 89 | Lebanon | 29.26 (17.01, 47.39) |
| 90 | Samoa | 29.25 (17.09, 47.96) |
| 91 | Tonga | 29.25 (16.96, 47.59) |
| 92 | Uzbekistan | 28.93 (16.56, 46.98) |
| 93 | Dominica | 28.92 (16.62, 45.81) |
| 94 | Mexico | 28.92 (16.71, 46.48) |
| 95 | Venezuela (Bolivarian Republic of) | 28.92 (16.92, 46.44) |
| 96 | Saint Vincent and the Grenadines | 28.61 (16.58, 45.97) |
| 97 | Iraq | 28.55 (16.67, 44.95) |
| 98 | Viet Nam | 28.53 (16.33, 46.12) |
| 99 | Armenia | 28.34 (16.50, 45.85) |
| 100 | Colombia | 28.24 (16.69, 45.43) |
| 101 | Ecuador | 28.24 (16.30, 45.04) |
| 102 | Tokelau | 28.20 (16.10, 45.63) |
| 103 | Brazil | 28.18 (16.34, 45.39) |
| 104 | Saint Lucia | 27.93 (16.61, 44.59) |
| 105 | Botswana | 27.90 (16.25, 45.55) |
| 106 | Georgia | 27.70 (16.17, 44.97) |
| 107 | Russian Federation | 27.60 (16.04, 44.63) |
| 108 | Costa Rica | 27.59 (16.01, 44.62) |
| 109 | Paraguay | 27.56 (16.05, 43.94) |
| 110 | Grenada | 27.46 (16.01, 43.99) |
| 111 | Suriname | 27.27 (15.68, 43.25) |
| 112 | Albania | 27.13 (15.75, 43.87) |
| 113 | Barbados | 27.07 (15.67, 42.75) |
| 114 | Mongolia | 27.05 (15.80, 44.00) |
| 115 | Belarus | 27.03 (15.85, 43.79) |
| 116 | Bosnia and Herzegovina | 27.00 (15.75, 43.29) |
| 117 | Dominican Republic | 26.92 (15.53, 43.51) |
| 118 | Fiji | 26.61 (15.28, 43.66) |
| 119 | China | 26.52 (15.30, 42.94) |
| 120 | Peru | 26.52 (15.51, 42.69) |
| 121 | Gabon | 26.50 (15.45, 41.89) |
| 122 | Sri Lanka | 26.47 (15.42, 42.16) |
| 123 | Türkiye | 26.31 (15.46, 41.85) |
| 124 | North Macedonia | 26.29 (15.05, 42.30) |
| 125 | Thailand | 25.94 (14.89, 41.51) |
| 126 | Panama | 25.93 (15.13, 41.50) |
| 127 | Nauru | 25.79 (14.97, 41.35) |
| 128 | Azerbaijan | 25.77 (14.91, 42.23) |
| 129 | Serbia | 25.75 (14.95, 41.84) |
| 130 | Niue | 25.57 (14.84, 41.11) |
| 131 | Latvia | 25.47 (14.88, 40.34) |
| 132 | Montenegro | 25.26 (14.76, 41.22) |
| 133 | Antigua and Barbuda | 25.20 (14.50, 40.14) |
| 134 | Maldives | 25.18 (14.60, 41.09) |
| 135 | Estonia | 24.92 (14.55, 39.66) |
| 136 | Lithuania | 24.83 (14.09, 39.07) |
| 137 | American Samoa | 24.82 (14.18, 40.05) |
| 138 | Turkmenistan | 24.78 (14.26, 40.09) |
| 139 | Equatorial Guinea | 24.76 (14.34, 40.04) |
| 140 | Poland | 24.66 (14.36, 40.10) |
| 141 | Bulgaria | 24.62 (14.05, 39.80) |
| 142 | Palau | 24.60 (14.27, 39.75) |
| 143 | Saint Kitts and Nevis | 24.50 (14.32, 39.18) |
| 144 | Bahrain | 24.34 (14.24, 39.18) |
| 145 | Bahamas | 24.14 (13.86, 38.19) |
| 146 | Kazakhstan | 23.92 (13.72, 38.49) |
| 147 | Croatia | 23.87 (13.85, 38.55) |
| 148 | Saudi Arabia | 23.87 (13.62, 38.19) |
| 149 | Romania | 23.83 (13.65, 38.65) |
| 150 | Mauritius | 23.80 (13.68, 38.53) |
| 151 | Oman | 23.73 (13.70, 37.60) |
| 152 | Trinidad and Tobago | 23.71 (13.46, 38.20) |
| 153 | Hungary | 23.50 (13.49, 37.86) |
| 154 | Puerto Rico | 23.47 (13.56, 37.36) |
| 155 | Malaysia | 22.96 (13.11, 36.69) |
| 156 | Seychelles | 22.93 (13.23, 36.56) |
| 157 | Kuwait | 22.84 (13.21, 36.55) |
| 158 | Slovakia | 22.78 (13.34, 37.14) |
| 159 | United Arab Emirates | 22.72 (13.06, 36.97) |
| 160 | Czechia | 22.39 (12.75, 36.35) |
| 161 | United States Virgin Islands | 22.38 (12.69, 35.56) |
| 162 | Slovenia | 22.29 (12.64, 36.23) |
| 163 | Northern Mariana Islands | 21.61 (12.38, 34.98) |
| 164 | Bermuda | 21.60 (12.31, 34.31) |
| 165 | Qatar | 21.56 (12.50, 34.98) |
| 166 | Cook Islands | 21.46 (12.28, 34.20) |
| 167 | Greece | 21.01 (12.02, 33.90) |
| 168 | Argentina | 20.82 (12.24, 33.46) |
| 169 | Spain | 20.79 (11.61, 32.92) |
| 170 | Uruguay | 20.28 (11.68, 32.43) |
| 171 | United Kingdom | 20.23 (11.55, 32.39) |
| 172 | Italy | 20.22 (11.68, 31.99) |
| 173 | Chile | 20.00 (11.59, 32.09) |
| 174 | Guam | 19.99 (11.44, 32.56) |
| 175 | Israel | 19.98 (11.47, 32.02) |
| 176 | France | 19.86 (11.32, 32.09) |
| 177 | Sweden | 19.80 (11.38, 31.51) |
| 178 | Norway | 19.79 (11.23, 31.79) |
| 179 | Japan | 19.78 (11.30, 31.77) |
| 180 | Cyprus | 19.68 (11.26, 31.08) |
| 181 | Portugal | 19.59 (10.93, 31.35) |
| 182 | Malta | 19.35 (11.00, 30.62) |
| 183 | Belgium | 18.99 (10.86, 30.27) |
| 184 | Taiwan (Province of China) | 18.61 (10.74, 29.85) |
| 185 | Iceland | 18.49 (10.54, 29.50) |
| 186 | Finland | 18.42 (10.39, 29.59) |
| 187 | Republic of Korea | 18.28 (10.53, 29.35) |
| 188 | Netherlands | 18.22 (10.17, 29.29) |
| 189 | Germany | 18.22 (10.41, 28.94) |
| 190 | Denmark | 18.13 (10.17, 28.63) |
| 191 | San Marino | 17.97 (10.11, 28.88) |
| 192 | United States of America | 17.75 (10.16, 28.76) |
| 193 | Ireland | 17.49 (9.96, 27.85) |
| 194 | Switzerland | 17.41 (9.78, 27.59) |
| 195 | Canada | 17.34 (9.87, 27.81) |
| 196 | Austria | 17.26 (9.83, 27.35) |
| 197 | Andorra | 17.25 (9.84, 27.38) |
| 198 | New Zealand | 16.61 (9.44, 26.81) |
| 199 | Brunei Darussalam | 16.54 (9.34, 26.46) |
| 200 | Luxembourg | 16.36 (9.24, 26.20) |
| 201 | Singapore | 16.08 (9.18, 25.47) |
| 202 | Greenland | 15.88 (8.98, 25.48) |
| 203 | Australia | 14.53 (8.22, 23.33) |
| 204 | Monaco | 13.43 (7.53, 21.99) |

| **Table S4. Net Drift values (percent change per year) for prevalence, incidence, and YLDS of OM by SDI level, 1990-2021.** | | | | | | | | | |
| --- | --- | --- | --- | --- | --- | --- | --- | --- | --- |
| SDI Level | **Prevalence** | | | **Incidence** | | | **YLDs** | | |
|  | **Net Drift Percent Per Year** | **CILo** | **CIHi** | **Net Drift Percent Per Year** | **CILo** | **CIHi** | **Net Drift Percent Per Year** | **CILo** | **CIHi** |
| Global | -0.661164217 | -0.730789423 | -0.591490178 | 0.0317806 | 0.009064276 | 0.054502083 | -0.669971378 | -0.747989614 | -0.591891814 |
| High SDI | -0.516872321 | -0.551151817 | -0.482581008 | -0.018284988 | -0.031368222 | -0.005200043 | -0.536740372 | -0.571515575 | -0.501953006 |
| High-middle SDI | -0.974839475 | -1.031300928 | -0.918345811 | -0.070712635 | -0.094849227 | -0.046570211 | -0.991259371 | -1.048956326 | -0.933528774 |
| Middle SDI | -1.033499737 | -1.13399702 | -0.932900299 | 0.034479825 | 0.00305827 | 0.065911254 | -1.053962949 | -1.161780432 | -0.946027852 |
| Low-middle SDI | -0.729612914 | -0.884278739 | -0.574705739 | 0.007925639 | -0.006905244 | 0.022758721 | -0.732581067 | -0.898764695 | -0.566118763 |
| Low SDI | -0.413932269 | -0.558186061 | -0.269469218 | 0.00093253 | -0.014402471 | 0.016269883 | -0.402646624 | -0.553660323 | -0.251403604 |

| **Table S5. Statistical tests for Age-Period-Cohort effects for prevalence, incidence, and YLDs of OM by SDI level, 1990-2021.** | | | | | | | | | | |
| --- | --- | --- | --- | --- | --- | --- | --- | --- | --- | --- |
| **SDI Level** | **Test** | **Prevalence** | | | **Incidence** | | | **YLDs** | | |
|  |  | **X2** | **df** | **P-Value** | **X2** | **df** | **P-Value** | **X2** | **df** | **P-Value** |
| **Global** | NetDrift = 0 | 343.8831026 | 1 | 9.10E-77 | 7.519683962 | 1 | 0.006102839 | 281.1707127 | 1 | 4.17E-63 |
|  | THETAa = 0 | 87.28328222 | 1 | 9.40E-21 | 10615.17383 | 1 | 0 | 30.48675126 | 1 | 3.36E-08 |
|  | All Higher-Order Age Deviations = 0 | 5623.655631 | 17 | 0 | 636924.6171 | 17 | 0 | 6600.900074 | 17 | 0 |
|  | All Age Deviations = 0 | 6443.552904 | 18 | 0 | 2407966.87 | 18 | 0 | 6639.922238 | 18 | 0 |
|  | THETAp = 0 | 31.37162328 | 1 | 2.13E-08 | 60.66098172 | 1 | 6.78E-15 | 26.35965426 | 1 | 2.83E-07 |
|  | All Higher-Order Period Deviations = 0 | 2.360484358 | 3 | 0.501032831 | 8.603683835 | 3 | 0.035051685 | 1.048639246 | 3 | 0.789484948 |
|  | All Period Deviations = 0 | 33.62604268 | 4 | 8.89E-07 | 69.43387757 | 4 | 2.99E-14 | 27.34373418 | 4 | 1.69E-05 |
|  | THETAc = 0 | 0.008934491 | 1 | 0.924694159 | 2.976297279 | 1 | 0.084492363 | 0.00189881 | 1 | 0.965242907 |
|  | All Higher-Order Cohort Deviations = 0 | 293.1519863 | 22 | 2.98E-49 | 477.9183239 | 22 | 2.91E-87 | 263.9940734 | 22 | 2.26E-43 |
|  | All Cohort Deviations = 0 | 759.7806329 | 23 | 1.09E-145 | 574.1762487 | 23 | 1.17E-106 | 677.9769459 | 23 | 1.92E-128 |
|  | All Period RR = 1 | 368.592092 | 5 | 1.74E-77 | 78.41741812 | 5 | 1.80E-15 | 301.2657098 | 5 | 5.35E-63 |
|  | All Cohort RR = 1 | 1706.133824 | 24 | 0 | 2286.482006 | 24 | 0 | 1512.294311 | 24 | 4.78E-305 |
|  | All Local Drifts = Net Drift | 757.2800547 | 20 | 1.63E-147 | 536.3732201 | 20 | 6.90E-101 | 675.1623776 | 20 | 3.96E-130 |
|  | All Gradient Shifts = CAT | 227.5331412 | 6 | 2.57E-46 | 167.2031626 | 6 | 1.76E-33 | 203.8031225 | 6 | 2.94E-41 |
| **High SDI** | NetDrift = 0 | 868.5775756 | 1 | 6.65E-191 | 7.501274897 | 1 | 0.006165533 | 909.9365114 | 1 | 6.79E-200 |
|  | THETAa = 0 | 2101.802499 | 1 | 0 | 37722.28062 | 1 | 0 | 1170.796624 | 1 | 1.36E-256 |
|  | All Higher-Order Age Deviations = 0 | 21299.09566 | 17 | 0 | 1021947.384 | 17 | 0 | 18595.32737 | 17 | 0 |
|  | All Age Deviations = 0 | 35585.38309 | 18 | 0 | 3026783.982 | 18 | 0 | 25580.29753 | 18 | 0 |
|  | THETAp = 0 | 12.28863918 | 1 | 0.000455724 | 18.33883934 | 1 | 1.85E-05 | 14.67114986 | 1 | 0.00012799 |
|  | All Higher-Order Period Deviations = 0 | 1.424526132 | 3 | 0.699795874 | 38.11059683 | 3 | 2.68E-08 | 1.671376956 | 3 | 0.643315989 |
|  | All Period Deviations = 0 | 13.58873131 | 4 | 0.008730222 | 58.05556309 | 4 | 7.43E-12 | 16.23290606 | 4 | 0.002722044 |
|  | THETAc = 0 | 2.389313962 | 1 | 0.122167231 | 0.11681678 | 1 | 0.732512926 | 2.649705946 | 1 | 0.103569053 |
|  | All Higher-Order Cohort Deviations = 0 | 271.6433748 | 22 | 6.55E-45 | 467.0024661 | 22 | 5.42E-85 | 264.5503967 | 22 | 1.75E-43 |
|  | All Cohort Deviations = 0 | 470.5030497 | 23 | 4.75E-85 | 867.2213406 | 23 | 2.04E-168 | 433.6678131 | 23 | 2.02E-77 |
|  | All Period RR = 1 | 898.3665181 | 5 | 6.01E-192 | 64.01260726 | 5 | 1.80E-12 | 944.2507769 | 5 | 7.04E-202 |
|  | All Cohort RR = 1 | 1775.310144 | 24 | 0 | 1206.235386 | 24 | 1.15E-239 | 1934.001213 | 24 | 0 |
|  | All Local Drifts = Net Drift | 470.1997591 | 20 | 4.96E-87 | 820.5503737 | 20 | 6.13E-161 | 433.4188656 | 20 | 2.32E-79 |
|  | All Gradient Shifts = CAT | 187.9908299 | 6 | 6.80E-38 | 166.7771714 | 6 | 2.17E-33 | 175.9401068 | 6 | 2.47E-35 |
| **High-middle SDI** | NetDrift = 0 | 1133.362029 | 1 | 1.85E-248 | 32.94140516 | 1 | 9.50E-09 | 1122.012701 | 1 | 5.43E-246 |
|  | THETAa = 0 | 259.7247026 | 1 | 1.97E-58 | 6842.781456 | 1 | 0 | 138.9003377 | 1 | 4.63E-32 |
|  | All Higher-Order Age Deviations = 0 | 8596.356683 | 17 | 0 | 354257.2758 | 17 | 0 | 9682.621025 | 17 | 0 |
|  | All Age Deviations = 0 | 11357.46862 | 18 | 0 | 1202809.583 | 18 | 0 | 10813.14576 | 18 | 0 |
|  | THETAp = 0 | 68.5822661 | 1 | 1.22E-16 | 70.80672833 | 1 | 3.94E-17 | 64.45031169 | 1 | 9.90E-16 |
|  | All Higher-Order Period Deviations = 0 | 61.03753592 | 3 | 3.53E-13 | 5.236644752 | 3 | 0.15526665 | 65.30090783 | 3 | 4.33E-14 |
|  | All Period Deviations = 0 | 138.324401 | 4 | 6.45E-29 | 78.38320893 | 4 | 3.83E-16 | 138.5759663 | 4 | 5.69E-29 |
|  | THETAc = 0 | 5.581154833 | 1 | 0.018154747 | 9.627923747 | 1 | 0.001916411 | 6.280108504 | 1 | 0.012210062 |
|  | All Higher-Order Cohort Deviations = 0 | 816.9116157 | 22 | 1.49E-158 | 327.937706 | 22 | 2.54E-56 | 855.1746386 | 22 | 1.15E-166 |
|  | All Cohort Deviations = 0 | 1152.577294 | 23 | 4.37E-229 | 464.7399408 | 23 | 7.45E-84 | 1160.406984 | 23 | 9.35E-231 |
|  | All Period RR = 1 | 1233.666139 | 5 | 1.50E-264 | 106.9509745 | 5 | 1.81E-21 | 1224.038879 | 5 | 1.82E-262 |
|  | All Cohort RR = 1 | 4994.776856 | 24 | 0 | 474.4028614 | 24 | 3.39E-85 | 5399.28364 | 24 | 0 |
|  | All Local Drifts = Net Drift | 1147.569004 | 20 | 1.21E-230 | 438.952545 | 20 | 1.63E-80 | 1154.346415 | 20 | 4.32E-232 |
|  | All Gradient Shifts = CAT | 224.9719719 | 6 | 9.05E-46 | 159.4518805 | 6 | 7.74E-32 | 215.5225656 | 6 | 9.37E-44 |
| **Middle SDI** | NetDrift = 0 | 401.6681116 | 1 | 2.39E-89 | 4.625941977 | 1 | 0.031491965 | 362.831797 | 1 | 6.81E-81 |
|  | THETAa = 0 | 48.39231666 | 1 | 3.49E-12 | 5736.677653 | 1 | 0 | 18.86574204 | 1 | 1.40E-05 |
|  | All Higher-Order Age Deviations = 0 | 4003.66948 | 17 | 0 | 460030.1857 | 17 | 0 | 4893.121496 | 17 | 0 |
|  | All Age Deviations = 0 | 4766.017479 | 18 | 0 | 1732166.047 | 18 | 0 | 5022.332727 | 18 | 0 |
|  | THETAp = 0 | 28.44825311 | 1 | 9.62E-08 | 122.366602 | 1 | 1.92E-28 | 25.43937969 | 1 | 4.57E-07 |
|  | All Higher-Order Period Deviations = 0 | 1.925113887 | 3 | 0.588094127 | 0.81182087 | 3 | 0.846637598 | 1.072665632 | 3 | 0.783676466 |
|  | All Period Deviations = 0 | 30.65247475 | 4 | 3.60E-06 | 123.6364795 | 4 | 8.93E-26 | 26.68191942 | 4 | 2.31E-05 |
|  | THETAc = 0 | 3.00E-05 | 1 | 0.995633251 | 0.799138795 | 1 | 0.37135098 | 0.000139259 | 1 | 0.99058455 |
|  | All Higher-Order Cohort Deviations = 0 | 423.7834827 | 22 | 5.00E-76 | 233.4185928 | 22 | 2.91E-37 | 417.0012904 | 22 | 1.26E-74 |
|  | All Cohort Deviations = 0 | 1013.992816 | 23 | 1.41E-199 | 293.8948126 | 23 | 7.82E-49 | 977.8576023 | 23 | 6.79E-192 |
|  | All Period RR = 1 | 423.4262878 | 5 | 2.64E-89 | 129.4505161 | 5 | 3.11E-26 | 381.694424 | 5 | 2.61E-80 |
|  | All Cohort RR = 1 | 2778.843307 | 24 | 0 | 1282.139783 | 24 | 7.40E-256 | 2761.751553 | 24 | 0 |
|  | All Local Drifts = Net Drift | 1013.687134 | 20 | 4.70E-202 | 258.4432332 | 20 | 2.25E-43 | 977.5235034 | 20 | 2.42E-194 |
|  | All Gradient Shifts = CAT | 271.5665184 | 6 | 1.00E-55 | 54.30115534 | 6 | 6.41E-10 | 261.5423335 | 6 | 1.40E-53 |
| **Low-middle SDI** | NetDrift = 0 | 84.73232672 | 1 | 3.42E-20 | 1.097025459 | 1 | 0.29491982 | 73.98169231 | 1 | 7.88E-18 |
|  | THETAa = 0 | 10.8731523 | 1 | 0.000975683 | 28454.75577 | 1 | 0 | 3.817547215 | 1 | 0.050718433 |
|  | All Higher-Order Age Deviations = 0 | 2082.211947 | 17 | 0 | 2640188.847 | 17 | 0 | 3091.895685 | 17 | 0 |
|  | All Age Deviations = 0 | 2136.183258 | 18 | 0 | 10920900.83 | 18 | 0 | 3131.481874 | 18 | 0 |
|  | THETAp = 0 | 24.84223217 | 1 | 6.22E-07 | 46.35053357 | 1 | 9.89E-12 | 23.55563112 | 1 | 1.21E-06 |
|  | All Higher-Order Period Deviations = 0 | 1.986817308 | 3 | 0.575147325 | 5.780436817 | 3 | 0.122795028 | 3.088855965 | 3 | 0.378127197 |
|  | All Period Deviations = 0 | 27.16017736 | 4 | 1.85E-05 | 52.3588073 | 4 | 1.16E-10 | 27.13050749 | 4 | 1.87E-05 |
|  | THETAc = 0 | 0.100665774 | 1 | 0.751032137 | 0.179197189 | 1 | 0.672064073 | 0.09850092 | 1 | 0.753636059 |
|  | All Higher-Order Cohort Deviations = 0 | 147.0612408 | 22 | 1.71E-20 | 621.6582773 | 22 | 2.44E-117 | 139.1918149 | 22 | 5.10E-19 |
|  | All Cohort Deviations = 0 | 329.2821083 | 23 | 5.29E-56 | 868.213783 | 23 | 1.26E-168 | 306.9368031 | 23 | 1.81E-51 |
|  | All Period RR = 1 | 108.9608909 | 5 | 6.79E-22 | 53.2034016 | 5 | 3.05E-10 | 98.54697229 | 5 | 1.07E-19 |
|  | All Cohort RR = 1 | 817.4198681 | 24 | 4.32E-157 | 1186.540435 | 24 | 1.81E-235 | 798.7450102 | 24 | 3.81E-153 |
|  | All Local Drifts = Net Drift | 329.1546031 | 20 | 8.65E-58 | 863.2757383 | 20 | 5.10E-170 | 306.7910674 | 20 | 3.31E-53 |
|  | All Gradient Shifts = CAT | 48.06309504 | 6 | 1.15E-08 | 4.176085307 | 6 | 0.652860916 | 43.29704948 | 6 | 1.02E-07 |
| **Low SDI** | NetDrift = 0 | 31.45464059 | 1 | 2.04E-08 | 0.0142039 | 1 | 0.905132658 | 27.15913129 | 1 | 1.87E-07 |
|  | THETAa = 0 | 1.492178895 | 1 | 0.2218787 | 20902.51822 | 1 | 0 | 0.004247413 | 1 | 0.948036909 |
|  | All Higher-Order Age Deviations = 0 | 4006.105739 | 17 | 0 | 4788133.936 | 17 | 0 | 5325.209055 | 17 | 0 |
|  | All Age Deviations = 0 | 4027.591502 | 18 | 0 | 18696128.58 | 18 | 0 | 5674.622638 | 18 | 0 |
|  | THETAp = 0 | 93.02355204 | 1 | 5.17E-22 | 132.0795615 | 1 | 1.44E-30 | 98.61001332 | 1 | 3.07E-23 |
|  | All Higher-Order Period Deviations = 0 | 1.73005619 | 3 | 0.63027242 | 4.186562721 | 3 | 0.24201066 | 1.013825583 | 3 | 0.797906623 |
|  | All Period Deviations = 0 | 94.11379946 | 4 | 1.76E-19 | 141.4487738 | 4 | 1.38E-29 | 100.2027604 | 4 | 8.91E-21 |
|  | THETAc = 0 | 0.235712366 | 1 | 0.627320167 | 0.876726527 | 1 | 0.349099827 | 0.235470783 | 1 | 0.627496665 |
|  | All Higher-Order Cohort Deviations = 0 | 156.3728858 | 22 | 2.98E-22 | 370.822212 | 22 | 4.19E-65 | 162.2839446 | 22 | 2.24E-23 |
|  | All Cohort Deviations = 0 | 304.0223939 | 23 | 7.04E-51 | 714.6730917 | 23 | 3.59E-136 | 303.6682733 | 23 | 8.30E-51 |
|  | All Period RR = 1 | 123.37573 | 5 | 6.05E-25 | 141.4502506 | 5 | 8.80E-29 | 125.3426498 | 5 | 2.31E-25 |
|  | All Cohort RR = 1 | 691.5269781 | 24 | 1.50E-130 | 2630.057599 | 24 | 0 | 698.8288614 | 24 | 4.37E-132 |
|  | All Local Drifts = Net Drift | 303.8121521 | 20 | 1.35E-52 | 712.3096633 | 20 | 5.50E-138 | 303.4484178 | 20 | 1.60E-52 |
|  | All Gradient Shifts = CAT | 13.98420809 | 6 | 0.029813067 | 19.86938625 | 6 | 0.002921581 | 12.85431781 | 6 | 0.045408615 |

| **Table S6. Estimated parameters from Age-Period-Cohort analysis for prevalence, incidence, and YLDs of OM by SDI level, 1990-2021.** | | | | | | | | | | | |
| --- | --- | --- | --- | --- | --- | --- | --- | --- | --- | --- | --- |
| **sdi level** | **result type** | **x value** | **Prevalence** | | | **Incidence** | | | **YLDs** | | |
|  |  |  | **y value** | **CILo** | **CIHi** | **y value** | **CILo** | **CIHi** | **y value** | **CILo** | **CIHi** |
| **Global** | **longitudinal age curve** | 2.5 | 3750.644515 | 3676.714616 | 3826.060966 | 24966.29489 | 24827.60064 | 25105.76392 | 66.99471735 | 65.56148953 | 68.45927671 |
|  |  | 7.5 | 3697.115743 | 3625.748509 | 3769.887731 | 12567.03486 | 12497.22037 | 12637.23936 | 75.18762325 | 73.62373176 | 76.78473441 |
|  |  | 12.5 | 2771.6588 | 2718.521086 | 2825.835173 | 4891.367182 | 4864.031895 | 4918.856089 | 59.74832398 | 58.51793976 | 61.00457796 |
|  |  | 17.5 | 2608.17209 | 2559.227849 | 2658.05237 | 2860.846793 | 2844.828991 | 2876.954783 | 54.98558295 | 53.87544442 | 56.11859661 |
|  |  | 22.5 | 2173.845933 | 2133.854102 | 2214.587275 | 1705.536584 | 1695.853455 | 1715.275003 | 45.81837718 | 44.91145121 | 46.7436173 |
|  |  | 27.5 | 1670.420757 | 1640.060749 | 1701.342775 | 1417.88363 | 1409.89594 | 1425.916574 | 35.05983167 | 34.37402915 | 35.75931676 |
|  |  | 32.5 | 1379.413522 | 1354.63223 | 1404.648157 | 1413.614236 | 1405.869266 | 1421.401873 | 28.734573 | 28.17850597 | 29.3016133 |
|  |  | 37.5 | 1282.714546 | 1261.725512 | 1304.052736 | 1691.466589 | 1683.276722 | 1699.696304 | 26.49594519 | 26.02845229 | 26.97183464 |
|  |  | 42.5 | 1268.879401 | 1248.558768 | 1289.530757 | 1659.042316 | 1651.142802 | 1666.979624 | 26.14979211 | 25.6979354 | 26.60959399 |
|  |  | 47.5 | 1299.911281 | 1279.223491 | 1320.933637 | 1320.551426 | 1313.872566 | 1327.264238 | 26.79475431 | 26.33458763 | 27.26296186 |
|  |  | 52.5 | 1303.065876 | 1281.754769 | 1324.731313 | 1230.475927 | 1223.883604 | 1237.103759 | 26.7195605 | 26.24731498 | 27.20030273 |
|  |  | 57.5 | 1114.928078 | 1094.740126 | 1135.488314 | 1387.600741 | 1379.931223 | 1395.312885 | 22.59807353 | 22.15427761 | 23.05075959 |
|  |  | 62.5 | 905.0898732 | 886.1282899 | 924.4572008 | 1371.70461 | 1363.349776 | 1380.110643 | 18.14256924 | 17.72868347 | 18.56611739 |
|  |  | 67.5 | 736.2555682 | 716.8767001 | 756.1582928 | 1179.475917 | 1170.398722 | 1188.623511 | 14.59078485 | 14.17136495 | 15.02261803 |
|  |  | 72.5 | 625.6554066 | 605.861281 | 646.0962272 | 1039.611879 | 1030.041545 | 1049.271132 | 12.24879973 | 11.82360238 | 12.68928791 |
|  |  | 77.5 | 558.3562361 | 536.8085184 | 580.7688881 | 950.9365992 | 940.3029374 | 961.6905145 | 10.79639611 | 10.33690702 | 11.2763101 |
|  |  | 82.5 | 527.7536815 | 502.0983278 | 554.7199283 | 847.0070969 | 834.7872708 | 859.4057999 | 10.09054322 | 9.546807308 | 10.66524746 |
|  |  | 87.5 | 545.819963 | 510.3316849 | 583.7760829 | 725.4588102 | 710.2632008 | 740.9795195 | 10.2998926 | 9.553121216 | 11.10503941 |
|  |  | 92.5 | 583.5270842 | 526.4557788 | 646.7852983 | 699.046964 | 675.3864063 | 723.5364131 | 10.83441897 | 9.644773433 | 12.17080268 |
|  |  | 97.5 | 623.8086698 | 520.9587618 | 746.9636467 | 774.2411835 | 728.7372832 | 822.5864438 | 11.35780563 | 9.244892426 | 13.95362356 |
|  | **period rate ratio** | 1994.5 | 1.047187468 | 1.036361839 | 1.058126179 | 0.99021074 | 0.987420466 | 0.993008899 | 1.049207939 | 1.037280058 | 1.06127298 |
|  |  | 1999.5 | 1.025545468 | 1.016515845 | 1.0346553 | 0.996673418 | 0.994666581 | 0.998684305 | 1.025967401 | 1.01612103 | 1.035909186 |
|  |  | 2004.5 | 1 | 1 | 1 | 1 | 1 | 1 | 1 | 1 | 1 |
|  |  | 2009.5 | 0.964651511 | 0.95621464 | 0.973162823 | 1.000444495 | 0.998445913 | 1.002447077 | 0.964851852 | 0.955656507 | 0.974135675 |
|  |  | 2014.5 | 0.925253096 | 0.915569301 | 0.935039315 | 0.999564165 | 0.996757723 | 1.00237851 | 0.925829845 | 0.915175231 | 0.936608501 |
|  |  | 2019.5 | 0.889476087 | 0.87799308 | 0.901109276 | 0.999457503 | 0.995671534 | 1.003257867 | 0.888281247 | 0.87557648 | 0.901170362 |
|  | **cohort rate ratio** | 1897 | 1.345466244 | 0.720103675 | 2.513914978 | 0.95887152 | 0.76952366 | 1.194810036 | 1.353626532 | 0.662346865 | 2.766382518 |
|  |  | 1902 | 1.336751796 | 1.018428041 | 1.754572039 | 0.991496469 | 0.901718575 | 1.090212929 | 1.344161385 | 0.987165868 | 1.830259624 |
|  |  | 1907 | 1.322197395 | 1.139911802 | 1.533632645 | 1.008889147 | 0.960335113 | 1.059898048 | 1.327263591 | 1.122854044 | 1.568884799 |
|  |  | 1912 | 1.301986932 | 1.182157069 | 1.433963401 | 1.015008985 | 0.985172651 | 1.045748924 | 1.306274644 | 1.172334029 | 1.45551814 |
|  |  | 1917 | 1.296758939 | 1.208813238 | 1.391103019 | 1.008293571 | 0.98737154 | 1.029658932 | 1.301132493 | 1.203201715 | 1.407034037 |
|  |  | 1922 | 1.26307658 | 1.198777254 | 1.330824757 | 1.009832443 | 0.994508729 | 1.025392271 | 1.267363635 | 1.196090581 | 1.342883731 |
|  |  | 1927 | 1.243098615 | 1.193534501 | 1.294720986 | 1.021828653 | 1.009813589 | 1.033986676 | 1.246881103 | 1.192224115 | 1.304043817 |
|  |  | 1932 | 1.222120009 | 1.182592345 | 1.262968869 | 1.014808999 | 1.005092481 | 1.02461945 | 1.225819957 | 1.182428068 | 1.270804209 |
|  |  | 1937 | 1.18566487 | 1.153705798 | 1.218509247 | 1.013484905 | 1.005231705 | 1.021805865 | 1.188611665 | 1.153689525 | 1.224590898 |
|  |  | 1942 | 1.139938866 | 1.113410564 | 1.167099235 | 1.001249541 | 0.993943724 | 1.008609059 | 1.142341316 | 1.113471523 | 1.171959638 |
|  |  | 1947 | 1.086373773 | 1.064290712 | 1.108915037 | 0.996969034 | 0.990565084 | 1.003414385 | 1.088001443 | 1.064043925 | 1.112498378 |
|  |  | 1952 | 1.044060847 | 1.025155755 | 1.063314572 | 0.999942521 | 0.994331972 | 1.005584727 | 1.044982599 | 1.024513994 | 1.065860143 |
|  |  | 1957 | 1 | 1 | 1 | 1 | 1 | 1 | 1 | 1 | 1 |
|  |  | 1962 | 0.960042697 | 0.944059535 | 0.976296457 | 1.003299009 | 0.998172979 | 1.008451364 | 0.959199876 | 0.941959544 | 0.976755752 |
|  |  | 1967 | 0.925807274 | 0.910344731 | 0.941532454 | 1.007939924 | 1.002764903 | 1.013141653 | 0.924650178 | 0.907995328 | 0.941610519 |
|  |  | 1972 | 0.886303123 | 0.8710115 | 0.901863208 | 1.013010312 | 1.007707452 | 1.018341076 | 0.884197779 | 0.867756133 | 0.90095095 |
|  |  | 1977 | 0.839989653 | 0.824947076 | 0.855306526 | 1.017098605 | 1.011642744 | 1.02258389 | 0.836745586 | 0.820604297 | 0.853204374 |
|  |  | 1982 | 0.799507943 | 0.78480096 | 0.814490531 | 1.019885057 | 1.014283071 | 1.025517983 | 0.79614056 | 0.780378455 | 0.812221029 |
|  |  | 1987 | 0.768891876 | 0.754483946 | 0.783574945 | 1.015802507 | 1.01016396 | 1.021472528 | 0.765276444 | 0.749844444 | 0.781026039 |
|  |  | 1992 | 0.755684182 | 0.74129639 | 0.770351226 | 1.022916666 | 1.017232989 | 1.0286321 | 0.752317846 | 0.736890273 | 0.768068411 |
|  |  | 1997 | 0.754768696 | 0.74004484 | 0.769785497 | 1.038140414 | 1.0322915 | 1.044022468 | 0.752584334 | 0.736770936 | 0.768737137 |
|  |  | 2002 | 0.757590009 | 0.742456816 | 0.773031655 | 1.04823941 | 1.042296839 | 1.054215862 | 0.756515916 | 0.740229665 | 0.773160492 |
|  |  | 2007 | 0.757127174 | 0.74157799 | 0.773002388 | 1.051809789 | 1.045843432 | 1.057810183 | 0.75577977 | 0.739020835 | 0.772918751 |
|  |  | 2012 | 0.758985167 | 0.742740012 | 0.775585634 | 1.052811661 | 1.046837972 | 1.058819438 | 0.757652707 | 0.740027845 | 0.775697332 |
|  |  | 2017 | 0.763188899 | 0.744629847 | 0.782210515 | 1.048669098 | 1.042576979 | 1.054796816 | 0.763185805 | 0.742615665 | 0.78432573 |
|  | **local drifts** | 2.5 | 0.037442285 | -0.019370118 | 0.094286972 | 0.09708668 | 0.090151967 | 0.104021873 | 0.051942453 | -0.012973042 | 0.116900093 |
|  |  | 7.5 | -0.031644426 | -0.07751126 | 0.014243462 | 0.155647176 | 0.148250699 | 0.163044199 | -0.017756273 | -0.06829006 | 0.032803069 |
|  |  | 12.5 | -0.181527816 | -0.227942068 | -0.135091971 | 0.150503991 | 0.140204643 | 0.160804399 | -0.168038753 | -0.218026381 | -0.118026082 |
|  |  | 17.5 | -0.402797044 | -0.452202586 | -0.353366983 | 0.120639628 | 0.107423116 | 0.133857885 | -0.39342335 | -0.446352561 | -0.340465998 |
|  |  | 22.5 | -0.660428151 | -0.71354361 | -0.607284277 | 0.077529526 | 0.061862921 | 0.093198583 | -0.663197159 | -0.720100426 | -0.606261277 |
|  |  | 27.5 | -0.848342135 | -0.905762425 | -0.790888572 | 0.048435019 | 0.030960358 | 0.065912733 | -0.861614137 | -0.923229824 | -0.79996013 |
|  |  | 32.5 | -0.912279146 | -0.974938072 | -0.849580573 | 0.057901666 | 0.039009623 | 0.076797277 | -0.929024278 | -0.996362559 | -0.861640196 |
|  |  | 37.5 | -0.88926002 | -0.956760283 | -0.821713753 | 0.082576338 | 0.062475973 | 0.102680741 | -0.906931238 | -0.979607941 | -0.834201194 |
|  |  | 42.5 | -0.845444607 | -0.916912868 | -0.773924797 | 0.073428062 | 0.051699315 | 0.095161527 | -0.863158786 | -0.940242263 | -0.786015326 |
|  |  | 47.5 | -0.807638873 | -0.884476836 | -0.730741343 | 0.061162466 | 0.036789102 | 0.085541769 | -0.822745809 | -0.905776518 | -0.739645529 |
|  |  | 52.5 | -0.827588177 | -0.912432671 | -0.742671034 | 0.029915311 | 0.002690686 | 0.057147347 | -0.841634702 | -0.933593814 | -0.749590229 |
|  |  | 57.5 | -0.846713593 | -0.942987245 | -0.750346373 | -0.029295286 | -0.05907647 | 0.000494773 | -0.860159779 | -0.964965675 | -0.75524297 |
|  |  | 62.5 | -0.815299609 | -0.928457609 | -0.702012363 | -0.067487637 | -0.101040039 | -0.033923966 | -0.826930253 | -0.950747691 | -0.702958035 |
|  |  | 67.5 | -0.720251444 | -0.8576969 | -0.582615442 | -0.0991568 | -0.138980865 | -0.059316854 | -0.729176467 | -0.880407199 | -0.577714997 |
|  |  | 72.5 | -0.594628816 | -0.768841497 | -0.420110282 | -0.072225755 | -0.122767055 | -0.021658879 | -0.601896776 | -0.794688181 | -0.408730709 |
|  |  | 77.5 | -0.485234576 | -0.715259555 | -0.25467667 | -0.017778602 | -0.08584184 | 0.050331003 | -0.490376293 | -0.74644078 | -0.233651184 |
|  |  | 82.5 | -0.377414745 | -0.68955521 | -0.064293197 | 0.013497587 | -0.08217649 | 0.109263275 | -0.380494538 | -0.729973083 | -0.029785659 |
|  |  | 87.5 | -0.31875407 | -0.78626905 | 0.150963934 | 0.029070991 | -0.125000788 | 0.183380447 | -0.321450883 | -0.848063104 | 0.207958263 |
|  |  | 92.5 | -0.287833339 | -1.118304025 | 0.549612162 | 0.083940412 | -0.205877509 | 0.374600009 | -0.295625468 | -1.237013126 | 0.654735296 |
|  |  | 97.5 | -0.241124833 | -2.089056328 | 1.641683771 | 0.180367267 | -0.476038156 | 0.841101979 | -0.252701964 | -2.361471365 | 1.901612044 |
| **High SDI** | **longitudinal age curve** | 2.5 | 2983.796034 | 2936.074843 | 3032.292858 | 22640.85082 | 22533.00892 | 22749.20884 | 49.45394227 | 48.6550941 | 50.2659064 |
|  |  | 7.5 | 2287.382012 | 2251.480284 | 2323.856223 | 9430.450585 | 9385.471013 | 9475.645719 | 43.23129037 | 42.55271241 | 43.92068944 |
|  |  | 12.5 | 1486.999221 | 1463.760814 | 1510.606558 | 3922.750396 | 3903.887197 | 3941.704739 | 29.9295851 | 29.46420551 | 30.40231523 |
|  |  | 17.5 | 1324.041057 | 1304.064817 | 1344.323302 | 2249.689897 | 2238.836556 | 2260.595852 | 26.53667565 | 26.13827207 | 26.94115176 |
|  |  | 22.5 | 950.5212652 | 936.4296579 | 964.8249262 | 1385.46519 | 1378.708837 | 1392.254652 | 19.83487936 | 19.54379301 | 20.13030116 |
|  |  | 27.5 | 795.8939988 | 784.4997742 | 807.4537153 | 1300.566334 | 1294.475856 | 1306.685468 | 16.4918922 | 16.2580331 | 16.72911519 |
|  |  | 32.5 | 714.0498946 | 704.2548658 | 723.9811562 | 1333.888222 | 1327.974922 | 1339.827853 | 14.67044378 | 14.47085101 | 14.87278947 |
|  |  | 37.5 | 707.9513612 | 699.3958769 | 716.6115019 | 1491.458285 | 1485.721036 | 1497.217688 | 14.45384216 | 14.28045552 | 14.62933398 |
|  |  | 42.5 | 745.9283647 | 737.3665909 | 754.5895517 | 1439.048273 | 1433.66327 | 1444.453502 | 15.24418768 | 15.0705184 | 15.41985829 |
|  |  | 47.5 | 831.1983989 | 822.063398 | 840.4349105 | 1179.136273 | 1174.544346 | 1183.746152 | 17.05363306 | 16.86777122 | 17.24154286 |
|  |  | 52.5 | 877.9556392 | 868.3385697 | 887.6792201 | 1091.192379 | 1086.76268 | 1095.640133 | 17.95308722 | 17.75789608 | 18.15042386 |
|  |  | 57.5 | 742.7102196 | 733.8860827 | 751.6404565 | 1176.904313 | 1172.004132 | 1181.824982 | 15.01527907 | 14.83764159 | 15.19504325 |
|  |  | 62.5 | 592.288676 | 584.2760802 | 600.4111542 | 1136.580893 | 1131.41909 | 1141.766246 | 11.85087493 | 11.69070203 | 12.01324234 |
|  |  | 67.5 | 494.3248899 | 486.1540036 | 502.6331058 | 966.8816005 | 961.4698548 | 972.3238069 | 9.789609582 | 9.627543531 | 9.954403785 |
|  |  | 72.5 | 437.3430122 | 429.0319786 | 445.8150437 | 848.1206983 | 842.6199258 | 853.6573809 | 8.567746457 | 8.404056309 | 8.734624882 |
|  |  | 77.5 | 412.6790522 | 403.602044 | 421.9602022 | 781.5575426 | 775.6306278 | 787.5297475 | 8.003923824 | 7.826263939 | 8.185616673 |
|  |  | 82.5 | 413.6464465 | 403.0053867 | 424.5684756 | 701.4712495 | 695.013481 | 707.9890208 | 7.945943267 | 7.738819862 | 8.158610165 |
|  |  | 87.5 | 455.3831963 | 441.164057 | 470.060632 | 606.467782 | 599.0489724 | 613.9784685 | 8.632505161 | 8.357792138 | 8.91624775 |
|  |  | 92.5 | 513.5869288 | 491.8583375 | 536.2754137 | 592.2865948 | 581.5989013 | 603.1706896 | 9.560484834 | 9.144421226 | 9.995478991 |
|  |  | 97.5 | 585.5232775 | 547.2746345 | 626.4450916 | 672.9737767 | 653.4631519 | 693.0669354 | 10.6832298 | 9.957560296 | 11.46178336 |
|  | **period rate ratio** | 1994.5 | 1.062243192 | 1.054459838 | 1.070083997 | 0.996349664 | 0.994199019 | 0.998504961 | 1.065521084 | 1.057633262 | 1.073467733 |
|  |  | 1999.5 | 1.032131932 | 1.024850449 | 1.039465148 | 1.000497471 | 0.998633336 | 1.002365087 | 1.033528492 | 1.026154125 | 1.040955854 |
|  |  | 2004.5 | 1 | 1 | 1 | 1 | 1 | 1 | 1 | 1 | 1 |
|  |  | 2009.5 | 0.973518417 | 0.966619303 | 0.980466772 | 0.997494678 | 0.995628924 | 0.999363928 | 0.972426931 | 0.965457925 | 0.97944624 |
|  |  | 2014.5 | 0.953763091 | 0.946605325 | 0.960974981 | 0.993276459 | 0.991108999 | 0.995448659 | 0.952734919 | 0.945508209 | 0.960016863 |
|  |  | 2019.5 | 0.934032566 | 0.926369212 | 0.941759314 | 0.994804049 | 0.992203902 | 0.99741101 | 0.931955018 | 0.924221205 | 0.939753548 |
|  | **cohort rate ratio** | 1897 | 1.235327706 | 0.991449852 | 1.539194884 | 0.945748355 | 0.853282852 | 1.048233829 | 1.249799859 | 0.99364951 | 1.571982548 |
|  |  | 1902 | 1.235802419 | 1.114106827 | 1.370791006 | 0.997259867 | 0.951627642 | 1.045080238 | 1.247575811 | 1.120574768 | 1.388970598 |
|  |  | 1907 | 1.230293191 | 1.156820142 | 1.308432731 | 1.022568325 | 0.996363875 | 1.049461954 | 1.238848234 | 1.162835404 | 1.31982991 |
|  |  | 1912 | 1.220921599 | 1.16890653 | 1.275251283 | 1.031848967 | 1.014667825 | 1.049321033 | 1.228399365 | 1.174942691 | 1.284288172 |
|  |  | 1917 | 1.206069246 | 1.164768522 | 1.248834424 | 1.019994055 | 1.007068991 | 1.033085004 | 1.213695994 | 1.171441496 | 1.257474633 |
|  |  | 1922 | 1.193389272 | 1.160917223 | 1.226769597 | 1.032317393 | 1.022331692 | 1.042400629 | 1.200036257 | 1.166989817 | 1.234018497 |
|  |  | 1927 | 1.187768901 | 1.160579402 | 1.215595382 | 1.038043132 | 1.029772421 | 1.04638027 | 1.194034873 | 1.166492657 | 1.222227392 |
|  |  | 1932 | 1.173432088 | 1.150349734 | 1.196977602 | 1.039870739 | 1.032837516 | 1.046951856 | 1.178740656 | 1.155463929 | 1.20248629 |
|  |  | 1937 | 1.144110443 | 1.124696082 | 1.163859933 | 1.034241872 | 1.028140822 | 1.040379127 | 1.148423784 | 1.12893352 | 1.168250534 |
|  |  | 1942 | 1.103325995 | 1.086954811 | 1.119943755 | 1.021007058 | 1.015598175 | 1.026444749 | 1.106293862 | 1.089930889 | 1.12290249 |
|  |  | 1947 | 1.066188126 | 1.052215794 | 1.080345996 | 1.013482027 | 1.008737853 | 1.018248513 | 1.067664324 | 1.053744846 | 1.081767672 |
|  |  | 1952 | 1.03537871 | 1.022846464 | 1.048064505 | 1.007867136 | 1.003611916 | 1.012140398 | 1.036293172 | 1.023825668 | 1.048912497 |
|  |  | 1957 | 1 | 1 | 1 | 1 | 1 | 1 | 1 | 1 | 1 |
|  |  | 1962 | 0.966917537 | 0.9556176 | 0.978351093 | 1.004289997 | 1.000299717 | 1.008296195 | 0.965584512 | 0.954380591 | 0.976919961 |
|  |  | 1967 | 0.93929617 | 0.927848163 | 0.950885426 | 1.013360873 | 1.009203435 | 1.017535438 | 0.937050011 | 0.925719548 | 0.948519155 |
|  |  | 1972 | 0.910575371 | 0.898753918 | 0.922552314 | 1.01782789 | 1.013498458 | 1.022175816 | 0.907286059 | 0.895603522 | 0.919120987 |
|  |  | 1977 | 0.879029553 | 0.86685948 | 0.891370486 | 1.014932432 | 1.010433193 | 1.019451704 | 0.874576852 | 0.862561235 | 0.886759848 |
|  |  | 1982 | 0.851360066 | 0.839035459 | 0.863865709 | 1.009423708 | 1.004779404 | 1.014089479 | 0.846499153 | 0.834336583 | 0.858839023 |
|  |  | 1987 | 0.824236896 | 0.811856884 | 0.836805691 | 1.008065598 | 1.003299692 | 1.012854143 | 0.818999705 | 0.806775343 | 0.831409293 |
|  |  | 1992 | 0.798660828 | 0.786347767 | 0.811166694 | 1.000481362 | 0.995722681 | 1.005262785 | 0.792488992 | 0.780308742 | 0.80485937 |
|  |  | 1997 | 0.789925469 | 0.777270924 | 0.80278604 | 1.001924432 | 0.997041751 | 1.006831024 | 0.78181785 | 0.769298985 | 0.794540436 |
|  |  | 2002 | 0.788823953 | 0.775763849 | 0.802103925 | 1.008434064 | 1.00345831 | 1.013434491 | 0.779380268 | 0.766426384 | 0.792553094 |
|  |  | 2007 | 0.789925362 | 0.776364793 | 0.80372279 | 1.016940948 | 1.011904401 | 1.022002564 | 0.779988491 | 0.766472543 | 0.79374278 |
|  |  | 2012 | 0.790608009 | 0.776371607 | 0.805105466 | 1.022966108 | 1.017882485 | 1.02807512 | 0.780962059 | 0.766616843 | 0.795575708 |
|  |  | 2017 | 0.792892969 | 0.776534053 | 0.809596511 | 1.025547147 | 1.020268899 | 1.030852701 | 0.783690186 | 0.766756447 | 0.800997905 |
|  | **local drifts** | 2.5 | -0.018429297 | -0.069742911 | 0.032910665 | 0.111191376 | 0.103351225 | 0.119032142 | -0.033325749 | -0.089287155 | 0.022667002 |
|  |  | 7.5 | -0.13857112 | -0.182328568 | -0.094794491 | 0.073624282 | 0.065089176 | 0.082160116 | -0.164782278 | -0.210691742 | -0.118851692 |
|  |  | 12.5 | -0.295121167 | -0.340499119 | -0.249722553 | 0.022651155 | 0.01145423 | 0.033849333 | -0.326016525 | -0.37228797 | -0.27972359 |
|  |  | 17.5 | -0.454727555 | -0.50295972 | -0.406472009 | -0.035444986 | -0.04910674 | -0.021781365 | -0.48315636 | -0.531628985 | -0.434660113 |
|  |  | 22.5 | -0.587248362 | -0.637965754 | -0.536505083 | -0.070323829 | -0.085636246 | -0.055009066 | -0.611204598 | -0.661726856 | -0.560656645 |
|  |  | 27.5 | -0.650339989 | -0.702529708 | -0.598122839 | -0.056161951 | -0.072293396 | -0.040027903 | -0.670623142 | -0.722444349 | -0.618774884 |
|  |  | 32.5 | -0.642737767 | -0.69604354 | -0.58940338 | 0.002419947 | -0.014093334 | 0.018935955 | -0.663417892 | -0.716274523 | -0.610533122 |
|  |  | 37.5 | -0.638830122 | -0.692484058 | -0.585147198 | 0.047394085 | 0.030576363 | 0.064214635 | -0.662078327 | -0.715285545 | -0.608842595 |
|  |  | 42.5 | -0.642804147 | -0.696148527 | -0.589431113 | 0.055405365 | 0.037892654 | 0.072921141 | -0.666463738 | -0.719376093 | -0.613523184 |
|  |  | 47.5 | -0.63492386 | -0.688277668 | -0.581541388 | 0.023993391 | 0.005276381 | 0.042713905 | -0.655489188 | -0.708416624 | -0.602533538 |
|  |  | 52.5 | -0.645184697 | -0.701031631 | -0.589306354 | -0.041565639 | -0.061833285 | -0.021293882 | -0.664812365 | -0.720317963 | -0.609275735 |
|  |  | 57.5 | -0.663876677 | -0.725076686 | -0.60263894 | -0.122703623 | -0.144453497 | -0.100949012 | -0.683328983 | -0.74438769 | -0.622232715 |
|  |  | 62.5 | -0.645612213 | -0.715002031 | -0.576173898 | -0.160086883 | -0.183983911 | -0.136184134 | -0.66406879 | -0.733614292 | -0.594474565 |
|  |  | 67.5 | -0.575693331 | -0.655446034 | -0.495876604 | -0.135620827 | -0.162859612 | -0.10837461 | -0.594077028 | -0.674378339 | -0.513710796 |
|  |  | 72.5 | -0.461836353 | -0.555685476 | -0.367898662 | -0.084046085 | -0.116577146 | -0.05150443 | -0.478517799 | -0.57347111 | -0.383473805 |
|  |  | 77.5 | -0.333066957 | -0.448695085 | -0.217304528 | 0.00703444 | -0.034670991 | 0.048757271 | -0.346853731 | -0.464421049 | -0.229147548 |
|  |  | 82.5 | -0.235102874 | -0.377825326 | -0.092175953 | 0.042873087 | -0.011596765 | 0.097372612 | -0.245009342 | -0.390876612 | -0.098928464 |
|  |  | 87.5 | -0.188254165 | -0.383730358 | 0.007605609 | 0.065083686 | -0.016632017 | 0.146866175 | -0.197015481 | -0.398019108 | 0.004393784 |
|  |  | 92.5 | -0.172322165 | -0.491591282 | 0.147971316 | 0.124258206 | -0.019776671 | 0.268500586 | -0.186598961 | -0.517109659 | 0.145009789 |
|  |  | 97.5 | -0.144696893 | -0.802939846 | 0.51791397 | 0.294479361 | -0.014703759 | 0.604618562 | -0.16798709 | -0.853948814 | 0.522720597 |
| **High-middle SDI** | **longitudinal age curve** | 2.5 | 3919.119998 | 3845.859355 | 3993.7762 | 20157.06871 | 20025.95218 | 20289.0437 | 69.92839485 | 68.62330622 | 71.25830385 |
|  |  | 7.5 | 3849.423599 | 3780.216889 | 3919.897318 | 10756.66539 | 10686.89011 | 10826.89623 | 76.6872377 | 75.32554362 | 78.07354774 |
|  |  | 12.5 | 2784.955275 | 2735.719776 | 2835.076879 | 4636.024997 | 4605.815552 | 4666.432586 | 58.16158193 | 57.15011478 | 59.19095045 |
|  |  | 17.5 | 2480.343783 | 2438.11096 | 2523.30816 | 2783.858 | 2765.785372 | 2802.048721 | 51.0715288 | 50.21503268 | 51.9426338 |
|  |  | 22.5 | 1826.774225 | 1796.454143 | 1857.606041 | 1705.066682 | 1693.927419 | 1716.279197 | 38.58786126 | 37.95887658 | 39.22726831 |
|  |  | 27.5 | 1432.788779 | 1409.692294 | 1456.263678 | 1395.432844 | 1386.480711 | 1404.442779 | 30.15256329 | 29.6751577 | 30.63764924 |
|  |  | 32.5 | 1216.08133 | 1197.052693 | 1235.412451 | 1374.439064 | 1365.963663 | 1382.967053 | 25.41138373 | 25.02049391 | 25.80838034 |
|  |  | 37.5 | 1145.388228 | 1129.411231 | 1161.59124 | 1644.409829 | 1635.591928 | 1653.27527 | 23.72216639 | 23.39658128 | 24.05228232 |
|  |  | 42.5 | 1136.196302 | 1120.88781 | 1151.713869 | 1623.248583 | 1614.763747 | 1631.778004 | 23.46909921 | 23.1577249 | 23.7846602 |
|  |  | 47.5 | 1171.075828 | 1155.568138 | 1186.791631 | 1311.774211 | 1304.561054 | 1319.02725 | 24.18359582 | 23.86817135 | 24.5031887 |
|  |  | 52.5 | 1176.47038 | 1160.583148 | 1192.575093 | 1253.064801 | 1245.879014 | 1260.292033 | 24.15037481 | 23.82860545 | 24.47648918 |
|  |  | 57.5 | 996.5024466 | 981.6751698 | 1011.553675 | 1450.181901 | 1441.735105 | 1458.678185 | 20.17869986 | 19.88115348 | 20.48069938 |
|  |  | 62.5 | 803.2475876 | 789.4834331 | 817.2517117 | 1466.577829 | 1457.278009 | 1475.936998 | 16.06176418 | 15.78773809 | 16.34054652 |
|  |  | 67.5 | 658.9155665 | 644.8919863 | 673.2440983 | 1299.369126 | 1289.10893 | 1309.710985 | 13.02434847 | 12.74754239 | 13.30716525 |
|  |  | 72.5 | 567.2497581 | 552.9279477 | 581.9425287 | 1164.69891 | 1153.890162 | 1175.608907 | 11.0948268 | 10.81386474 | 11.38308872 |
|  |  | 77.5 | 512.5672037 | 497.0739811 | 528.5433321 | 1059.573518 | 1047.847389 | 1071.430871 | 9.931107973 | 9.62864758 | 10.24306942 |
|  |  | 82.5 | 489.9720772 | 471.6246455 | 509.0332719 | 928.5527293 | 915.4615658 | 941.8310973 | 9.406915631 | 9.050194238 | 9.777697512 |
|  |  | 87.5 | 513.1872929 | 487.4761368 | 540.2545432 | 768.2898498 | 752.3169982 | 784.6018296 | 9.739761681 | 9.242382365 | 10.26390749 |
|  |  | 92.5 | 552.0984799 | 509.3589242 | 598.4242486 | 715.9400273 | 690.7948789 | 742.000467 | 10.32360015 | 9.501907682 | 11.21634978 |
|  |  | 97.5 | 587.2047072 | 503.7169824 | 684.5299647 | 774.7236986 | 722.8203146 | 830.3540964 | 10.76709969 | 9.178521975 | 12.63062137 |
|  | **period rate ratio** | 1994.5 | 1.05645362 | 1.046635206 | 1.066364139 | 0.997570588 | 0.994236362 | 1.000915995 | 1.058746522 | 1.048872384 | 1.068713615 |
|  |  | 1999.5 | 1.041537333 | 1.032838414 | 1.050309517 | 1.001078693 | 0.998404377 | 1.003760173 | 1.04352419 | 1.034838242 | 1.052283043 |
|  |  | 2004.5 | 1 | 1 | 1 | 1 | 1 | 1 | 1 | 1 | 1 |
|  |  | 2009.5 | 0.934299841 | 0.926317523 | 0.942350944 | 0.995036692 | 0.992350232 | 0.997730424 | 0.932703653 | 0.924755588 | 0.940720029 |
|  |  | 2014.5 | 0.880156188 | 0.871599137 | 0.888797248 | 0.988476275 | 0.985116382 | 0.991847627 | 0.879361185 | 0.870780741 | 0.888026178 |
|  |  | 2019.5 | 0.840846393 | 0.831234517 | 0.850569415 | 0.981577644 | 0.977336683 | 0.985837007 | 0.839495114 | 0.82980461 | 0.849298784 |
|  | **cohort rate ratio** | 1897 | 1.486505161 | 0.863699304 | 2.558410761 | 1.071220914 | 0.831394011 | 1.380229146 | 1.487348097 | 0.844300553 | 2.620162162 |
|  |  | 1902 | 1.459117218 | 1.173489922 | 1.81426616 | 1.075396065 | 0.971955238 | 1.189845633 | 1.462355533 | 1.167501904 | 1.831674704 |
|  |  | 1907 | 1.428517898 | 1.274660297 | 1.600946847 | 1.081864245 | 1.029556821 | 1.136829188 | 1.429092211 | 1.271245439 | 1.606538348 |
|  |  | 1912 | 1.397426411 | 1.299386701 | 1.50286329 | 1.082526458 | 1.051793511 | 1.114157408 | 1.397231583 | 1.297203797 | 1.504972543 |
|  |  | 1917 | 1.37686888 | 1.30440117 | 1.453362628 | 1.059660908 | 1.038142044 | 1.08162582 | 1.378170019 | 1.30451092 | 1.455988273 |
|  |  | 1922 | 1.339117008 | 1.286420622 | 1.393972026 | 1.052355742 | 1.036803284 | 1.068141493 | 1.341368124 | 1.288033745 | 1.396910952 |
|  |  | 1927 | 1.305589013 | 1.26554297 | 1.346902247 | 1.067601525 | 1.055468718 | 1.079873801 | 1.306925994 | 1.266612994 | 1.348522052 |
|  |  | 1932 | 1.263888941 | 1.231715189 | 1.296903107 | 1.035848522 | 1.025985308 | 1.045806556 | 1.267569641 | 1.235278989 | 1.300704382 |
|  |  | 1937 | 1.218328226 | 1.192146715 | 1.245084727 | 1.03198909 | 1.023484814 | 1.04056403 | 1.221031967 | 1.19488389 | 1.247752252 |
|  |  | 1942 | 1.161549691 | 1.139334241 | 1.184198313 | 1.004119894 | 0.9964197 | 1.011879593 | 1.164331147 | 1.142230842 | 1.186859056 |
|  |  | 1947 | 1.106380806 | 1.087828425 | 1.125249589 | 0.995145152 | 0.988343266 | 1.00199385 | 1.10865352 | 1.090258471 | 1.127358933 |
|  |  | 1952 | 1.058172662 | 1.042521935 | 1.074058344 | 1.000158613 | 0.994210485 | 1.006142327 | 1.059009988 | 1.043536766 | 1.074712643 |
|  |  | 1957 | 1 | 1 | 1 | 1 | 1 | 1 | 1 | 1 | 1 |
|  |  | 1962 | 0.941642958 | 0.9285094 | 0.954962287 | 0.99990765 | 0.994344844 | 1.005501577 | 0.940660716 | 0.927731072 | 0.953770559 |
|  |  | 1967 | 0.889594866 | 0.87699591 | 0.902374819 | 1.001592588 | 0.995933943 | 1.007283384 | 0.887976484 | 0.875597495 | 0.900530485 |
|  |  | 1972 | 0.828959226 | 0.816591491 | 0.841514276 | 1.003276456 | 0.997420163 | 1.009167135 | 0.825388761 | 0.813273597 | 0.837684402 |
|  |  | 1977 | 0.756054865 | 0.744064937 | 0.768238001 | 1.004322015 | 0.998208741 | 1.010472728 | 0.750826989 | 0.739112589 | 0.762727054 |
|  |  | 1982 | 0.704035897 | 0.692404046 | 0.715863154 | 1.006883051 | 1.000540181 | 1.013266132 | 0.698572794 | 0.687221553 | 0.710111529 |
|  |  | 1987 | 0.666346351 | 0.655066472 | 0.677820463 | 1.000584951 | 0.994181676 | 1.007029468 | 0.660512721 | 0.64951194 | 0.671699821 |
|  |  | 1992 | 0.63097199 | 0.61988172 | 0.642260676 | 0.993069092 | 0.986643095 | 0.999536942 | 0.624181418 | 0.613364315 | 0.635189289 |
|  |  | 1997 | 0.608032105 | 0.596767324 | 0.619509524 | 1.000558962 | 0.993904858 | 1.007257615 | 0.600137227 | 0.589152502 | 0.611326762 |
|  |  | 2002 | 0.603755691 | 0.59210156 | 0.615639207 | 1.008830396 | 1.002026052 | 1.015680946 | 0.595504419 | 0.584117221 | 0.607113607 |
|  |  | 2007 | 0.608060455 | 0.595882418 | 0.620487374 | 1.010629749 | 1.00379429 | 1.017511755 | 0.59935965 | 0.587431863 | 0.611529629 |
|  |  | 2012 | 0.610109178 | 0.597197713 | 0.62329979 | 1.00900316 | 1.002156883 | 1.015896208 | 0.60167854 | 0.588916226 | 0.614717424 |
|  |  | 2017 | 0.611779172 | 0.596308042 | 0.627651699 | 0.997184064 | 0.99009914 | 1.004319686 | 0.604474971 | 0.588773436 | 0.620595239 |
|  | **local drifts** | 2.5 | -0.078320764 | -0.144880607 | -0.011716554 | 0.027243633 | 0.015707753 | 0.038780844 | -0.083539957 | -0.153490173 | -0.013540736 |
|  |  | 7.5 | -0.318849361 | -0.370967711 | -0.266703747 | 0.058708292 | 0.047397933 | 0.07001993 | -0.339965117 | -0.393051346 | -0.286850596 |
|  |  | 12.5 | -0.607137322 | -0.659007649 | -0.55523991 | 0.028978662 | 0.01438738 | 0.043572072 | -0.635681346 | -0.687432395 | -0.583903329 |
|  |  | 17.5 | -0.920918849 | -0.974745115 | -0.867063325 | -0.002312625 | -0.020060418 | 0.015438318 | -0.950341538 | -1.003594902 | -0.897059527 |
|  |  | 22.5 | -1.219527662 | -1.274696456 | -1.164328039 | -0.030646379 | -0.050562059 | -0.01072673 | -1.251365274 | -1.305653593 | -1.197047093 |
|  |  | 27.5 | -1.386803842 | -1.442959482 | -1.330616206 | -0.027564192 | -0.048833874 | -0.006289985 | -1.42020217 | -1.475333776 | -1.365039715 |
|  |  | 32.5 | -1.431343425 | -1.489715433 | -1.372936829 | 0.011561653 | -0.010787358 | 0.033915659 | -1.464725579 | -1.522000465 | -1.407417383 |
|  |  | 37.5 | -1.40926784 | -1.469822659 | -1.348675805 | 0.028113839 | 0.00494057 | 0.051292479 | -1.442574879 | -1.502063861 | -1.383049967 |
|  |  | 42.5 | -1.305989656 | -1.367749312 | -1.244191329 | 0.018440264 | -0.005862402 | 0.042748836 | -1.335522957 | -1.396286188 | -1.27472228 |
|  |  | 47.5 | -1.149976268 | -1.214763831 | -1.085146214 | 0.025657384 | -0.000991241 | 0.052313111 | -1.172975722 | -1.236834737 | -1.109075416 |
|  |  | 52.5 | -1.065106674 | -1.135424749 | -0.994738584 | 0.000893605 | -0.028218788 | 0.030014476 | -1.082710646 | -1.152259992 | -1.013112365 |
|  |  | 57.5 | -1.013039193 | -1.090613685 | -0.935403859 | -0.09436189 | -0.125058631 | -0.063655714 | -1.027031253 | -1.104129474 | -0.949872926 |
|  |  | 62.5 | -0.934144554 | -1.023454585 | -0.844753934 | -0.159342163 | -0.192879395 | -0.125793662 | -0.944988476 | -1.034229169 | -0.855667311 |
|  |  | 67.5 | -0.852106671 | -0.958086658 | -0.746013281 | -0.270444649 | -0.308995831 | -0.231878559 | -0.854120985 | -0.960626008 | -0.747501428 |
|  |  | 72.5 | -0.763920322 | -0.897672784 | -0.629987342 | -0.266576431 | -0.315450616 | -0.217678283 | -0.760928621 | -0.896047532 | -0.625625488 |
|  |  | 77.5 | -0.664262847 | -0.840376448 | -0.487836457 | -0.204368396 | -0.270358282 | -0.138334844 | -0.65817094 | -0.837034627 | -0.478984629 |
|  |  | 82.5 | -0.551601701 | -0.786027559 | -0.316621934 | -0.167202003 | -0.258982352 | -0.075337199 | -0.541931952 | -0.781282419 | -0.302004088 |
|  |  | 87.5 | -0.481099386 | -0.837360202 | -0.123558635 | -0.151822258 | -0.304927259 | 0.001517871 | -0.471571271 | -0.837355362 | -0.104437901 |
|  |  | 92.5 | -0.435954339 | -1.096072468 | 0.22856964 | -0.080358799 | -0.38611221 | 0.226333088 | -0.436551671 | -1.118480805 | 0.250080337 |
|  |  | 97.5 | -0.409531117 | -2.003041278 | 1.209890814 | -0.075655598 | -0.824791985 | 0.679139515 | -0.40884523 | -2.069163339 | 1.279621889 |
| **Middle SDI** | **longitudinal age curve** | 2.5 | 4232.753524 | 4130.966167 | 4337.048931 | 22866.86623 | 22726.74122 | 23007.85519 | 76.45773977 | 74.55637333 | 78.40759562 |
|  |  | 7.5 | 4254.451271 | 4154.880526 | 4356.408206 | 11892.42105 | 11819.61276 | 11965.67783 | 86.34950054 | 84.27606926 | 88.47394413 |
|  |  | 12.5 | 3166.140246 | 3092.817965 | 3241.200799 | 4793.51272 | 4764.014747 | 4823.193339 | 67.53847722 | 65.93853721 | 69.17723834 |
|  |  | 17.5 | 2901.821487 | 2836.242442 | 2968.916838 | 2887.154913 | 2869.390462 | 2905.029344 | 60.73490369 | 59.32883782 | 62.17429266 |
|  |  | 22.5 | 2277.710592 | 2227.233093 | 2329.332102 | 1762.437915 | 1751.474454 | 1773.470003 | 48.19204178 | 47.09904488 | 49.31040314 |
|  |  | 27.5 | 1772.290377 | 1733.70321 | 1811.736382 | 1431.735332 | 1422.89437 | 1440.631226 | 37.35808876 | 36.52554805 | 38.20960589 |
|  |  | 32.5 | 1479.799852 | 1448.040359 | 1512.255918 | 1420.704658 | 1412.157285 | 1429.303765 | 30.96090182 | 30.28022334 | 31.65688148 |
|  |  | 37.5 | 1377.60664 | 1350.741768 | 1405.005826 | 1736.562783 | 1727.317555 | 1745.857494 | 28.56030173 | 27.98925861 | 29.1429954 |
|  |  | 42.5 | 1353.108709 | 1327.225265 | 1379.49693 | 1714.305652 | 1705.319585 | 1723.33907 | 27.97720969 | 27.42812315 | 28.53728845 |
|  |  | 47.5 | 1366.778214 | 1340.719329 | 1393.343592 | 1350.459324 | 1342.918754 | 1358.042234 | 28.25941007 | 27.70655669 | 28.82329501 |
|  |  | 52.5 | 1353.517129 | 1326.93999 | 1380.626579 | 1251.564936 | 1244.13866 | 1259.035538 | 27.82538219 | 27.26390752 | 28.3984199 |
|  |  | 57.5 | 1150.597902 | 1125.434981 | 1176.323425 | 1420.019953 | 1411.276694 | 1428.817379 | 23.3827897 | 22.85544504 | 23.92230181 |
|  |  | 62.5 | 930.8054321 | 907.1324581 | 955.0961877 | 1403.051376 | 1393.450489 | 1412.718413 | 18.69710287 | 18.2046414 | 19.20288614 |
|  |  | 67.5 | 759.0343881 | 734.6291561 | 784.2503902 | 1197.577069 | 1187.030416 | 1208.217429 | 15.06882448 | 14.56558292 | 15.58945306 |
|  |  | 72.5 | 648.2457912 | 622.9721074 | 674.5448164 | 1052.017668 | 1040.708402 | 1063.449831 | 12.70670762 | 12.18970851 | 13.24563409 |
|  |  | 77.5 | 578.7300112 | 550.8466044 | 608.0248534 | 967.470849 | 954.5581284 | 980.5582456 | 11.19072104 | 10.62494321 | 11.78662653 |
|  |  | 82.5 | 547.0929587 | 513.0416718 | 583.4042768 | 882.7130323 | 867.039491 | 898.6699055 | 10.43282951 | 9.747206178 | 11.16667992 |
|  |  | 87.5 | 565.4034757 | 516.3491123 | 619.1181174 | 795.5526758 | 774.2293314 | 817.4632947 | 10.5978369 | 9.619183502 | 11.67605825 |
|  |  | 92.5 | 600.3606424 | 517.7313155 | 696.1775156 | 803.6124129 | 767.1760489 | 841.7792906 | 11.04924616 | 9.418066328 | 12.96294128 |
|  |  | 97.5 | 620.4031912 | 470.9955637 | 817.2054035 | 892.8562265 | 819.8445786 | 972.3699613 | 11.17756201 | 8.274641811 | 15.09888831 |
|  | **period rate ratio** | 1994.5 | 1.083854144 | 1.068951079 | 1.098964985 | 0.987313842 | 0.983680557 | 0.990960547 | 1.087605411 | 1.071850849 | 1.103591541 |
|  |  | 1999.5 | 1.043365556 | 1.031711575 | 1.055151177 | 0.994921608 | 0.99244512 | 0.997404275 | 1.044776733 | 1.032630453 | 1.057065883 |
|  |  | 2004.5 | 1 | 1 | 1 | 1 | 1 | 1 | 1 | 1 | 1 |
|  |  | 2009.5 | 0.947212891 | 0.936586038 | 0.95796032 | 1.001046714 | 0.998553538 | 1.003546116 | 0.946882984 | 0.935825715 | 0.9580709 |
|  |  | 2014.5 | 0.889489885 | 0.876990605 | 0.902167311 | 0.999739032 | 0.996054583 | 1.003437109 | 0.88911389 | 0.875954873 | 0.902470589 |
|  |  | 2019.5 | 0.838196908 | 0.82324571 | 0.853419638 | 0.996198359 | 0.991137936 | 1.001284619 | 0.835980062 | 0.820149574 | 0.85211611 |
|  | **cohort rate ratio** | 1897 | 1.614369932 | 0.571233973 | 4.562386696 | 0.978557373 | 0.699525941 | 1.368890667 | 1.626752558 | 0.522483929 | 5.064890489 |
|  |  | 1902 | 1.588645654 | 1.038199146 | 2.430935359 | 0.989458449 | 0.860896581 | 1.137219084 | 1.602779302 | 1.010909501 | 2.541178501 |
|  |  | 1907 | 1.562904348 | 1.254384143 | 1.947306186 | 0.996580295 | 0.929134723 | 1.068921717 | 1.576782372 | 1.244939118 | 1.997079706 |
|  |  | 1912 | 1.536979697 | 1.341274322 | 1.761240449 | 0.999281247 | 0.959089601 | 1.041157167 | 1.54927996 | 1.339787459 | 1.791529229 |
|  |  | 1917 | 1.494599254 | 1.362755058 | 1.639199148 | 0.997402473 | 0.971034211 | 1.02448676 | 1.506086991 | 1.365701812 | 1.660902844 |
|  |  | 1922 | 1.443300963 | 1.349212217 | 1.543951089 | 0.996210777 | 0.977296672 | 1.015490937 | 1.453847369 | 1.354232628 | 1.560789578 |
|  |  | 1927 | 1.387832615 | 1.318118093 | 1.4612343 | 0.997258969 | 0.982949192 | 1.011777067 | 1.3976669 | 1.324269296 | 1.475132565 |
|  |  | 1932 | 1.329313932 | 1.276140343 | 1.384703132 | 0.996690559 | 0.985433837 | 1.008075867 | 1.337732541 | 1.282051103 | 1.395832308 |
|  |  | 1937 | 1.263833365 | 1.221989985 | 1.307109547 | 0.997148577 | 0.987724067 | 1.006663012 | 1.270680211 | 1.227087323 | 1.315821757 |
|  |  | 1942 | 1.194206548 | 1.160342208 | 1.229059211 | 0.996909305 | 0.988602884 | 1.005285518 | 1.199129884 | 1.164023626 | 1.235294926 |
|  |  | 1947 | 1.127730279 | 1.09997775 | 1.156183006 | 0.996967961 | 0.989734026 | 1.004254769 | 1.130853749 | 1.102192791 | 1.160259995 |
|  |  | 1952 | 1.065486998 | 1.042313483 | 1.089175725 | 0.998085184 | 0.991842946 | 1.004366709 | 1.067310978 | 1.043437365 | 1.091730815 |
|  |  | 1957 | 1 | 1 | 1 | 1 | 1 | 1 | 1 | 1 | 1 |
|  |  | 1962 | 0.94004996 | 0.921362969 | 0.959115959 | 1.000221948 | 0.994597615 | 1.005878086 | 0.938928496 | 0.919767918 | 0.958488227 |
|  |  | 1967 | 0.888781456 | 0.871184434 | 0.906733919 | 0.999850003 | 0.994251145 | 1.00548039 | 0.887217864 | 0.8692031 | 0.905605995 |
|  |  | 1972 | 0.831676835 | 0.814569445 | 0.849143511 | 1.00390854 | 0.998162378 | 1.009687781 | 0.828746859 | 0.811274214 | 0.846595817 |
|  |  | 1977 | 0.760265719 | 0.743922031 | 0.776968471 | 1.00932028 | 1.003378096 | 1.015297655 | 0.755656206 | 0.7390103 | 0.772677055 |
|  |  | 1982 | 0.705003954 | 0.689380863 | 0.720981104 | 1.013796426 | 1.007675648 | 1.019954382 | 0.700164625 | 0.684275179 | 0.716423037 |
|  |  | 1987 | 0.666460534 | 0.651416331 | 0.681852177 | 1.008798545 | 1.002644787 | 1.014990072 | 0.660860562 | 0.645582054 | 0.676500656 |
|  |  | 1992 | 0.644050249 | 0.629207307 | 0.659243334 | 1.012296751 | 1.006103613 | 1.018528012 | 0.637911656 | 0.622836668 | 0.653351515 |
|  |  | 1997 | 0.636019042 | 0.620888166 | 0.651518653 | 1.026106203 | 1.019723494 | 1.032528863 | 0.630168837 | 0.614789084 | 0.645933335 |
|  |  | 2002 | 0.637234207 | 0.621648842 | 0.653210312 | 1.034859111 | 1.028367118 | 1.041392088 | 0.632143603 | 0.616266996 | 0.648429233 |
|  |  | 2007 | 0.637492993 | 0.621402858 | 0.653999755 | 1.03762903 | 1.031107811 | 1.044191493 | 0.632027943 | 0.61561254 | 0.648881065 |
|  |  | 2012 | 0.639980734 | 0.623046663 | 0.657375064 | 1.041224804 | 1.034672989 | 1.047818106 | 0.634422601 | 0.617026409 | 0.652309254 |
|  |  | 2017 | 0.64453901 | 0.624753933 | 0.664950652 | 1.036616978 | 1.029897116 | 1.043380686 | 0.639892188 | 0.619103262 | 0.661379187 |
|  | **local drifts** | 2.5 | 0.013045267 | -0.062163925 | 0.088311059 | 0.094476688 | 0.085950351 | 0.103003751 | 0.020287246 | -0.061471012 | 0.102112388 |
|  |  | 7.5 | -0.132201614 | -0.191316203 | -0.073052013 | 0.137713034 | 0.129017853 | 0.14640897 | -0.130661789 | -0.192828141 | -0.068456716 |
|  |  | 12.5 | -0.370955888 | -0.430019662 | -0.311857079 | 0.117924466 | 0.106176886 | 0.129673425 | -0.37495258 | -0.435762248 | -0.314105772 |
|  |  | 17.5 | -0.697999794 | -0.760174383 | -0.635786253 | 0.094107102 | 0.079347511 | 0.10886887 | -0.708152606 | -0.771775365 | -0.644489053 |
|  |  | 22.5 | -1.077000349 | -1.142651865 | -1.011305233 | 0.064731536 | 0.047559205 | 0.081906814 | -1.099944526 | -1.166988418 | -1.032855155 |
|  |  | 27.5 | -1.334008901 | -1.403393302 | -1.264575673 | 0.046217121 | 0.027262601 | 0.065175232 | -1.364795633 | -1.43565717 | -1.293883151 |
|  |  | 32.5 | -1.420939606 | -1.496007329 | -1.345814675 | 0.051226419 | 0.030666353 | 0.071790711 | -1.451441138 | -1.528160224 | -1.374662281 |
|  |  | 37.5 | -1.390787548 | -1.471501856 | -1.310007119 | 0.05700316 | 0.035005788 | 0.079005368 | -1.419448449 | -1.502072824 | -1.336754765 |
|  |  | 42.5 | -1.303775885 | -1.38886487 | -1.218613478 | 0.038464343 | 0.01461277 | 0.062321604 | -1.332035144 | -1.419268875 | -1.244724219 |
|  |  | 47.5 | -1.208874596 | -1.300608932 | -1.117055 | 0.022979602 | -0.004056673 | 0.050023187 | -1.23319265 | -1.327399919 | -1.138895437 |
|  |  | 52.5 | -1.185177488 | -1.28737683 | -1.082872336 | 0.015098123 | -0.015538153 | 0.045743787 | -1.209434486 | -1.314694045 | -1.104062656 |
|  |  | 57.5 | -1.17536911 | -1.292855614 | -1.057742768 | 0.014740248 | -0.019464434 | 0.048956632 | -1.201557591 | -1.323070452 | -1.079895097 |
|  |  | 62.5 | -1.132251112 | -1.271748178 | -0.992556945 | 0.011114913 | -0.028233557 | 0.05047887 | -1.157101194 | -1.302102735 | -1.011886625 |
|  |  | 67.5 | -1.063782412 | -1.235990752 | -0.891273804 | 0.002706086 | -0.045551338 | 0.050986808 | -1.085649816 | -1.265635852 | -0.905335678 |
|  |  | 72.5 | -0.986488482 | -1.208588572 | -0.763889074 | 0.00183216 | -0.06115259 | 0.064856604 | -1.004775631 | -1.238306552 | -0.770692505 |
|  |  | 77.5 | -0.889342334 | -1.188292646 | -0.589487562 | -0.000125848 | -0.086735147 | 0.086558528 | -0.902986876 | -1.219353243 | -0.585607277 |
|  |  | 82.5 | -0.779304126 | -1.210652235 | -0.346072603 | -0.006727127 | -0.13654336 | 0.12325786 | -0.788993949 | -1.248614277 | -0.327234401 |
|  |  | 87.5 | -0.655310663 | -1.337825618 | 0.031925723 | -0.003839745 | -0.221082596 | 0.213876095 | -0.664247227 | -1.396813991 | 0.0737621 |
|  |  | 92.5 | -0.537119887 | -1.819405658 | 0.761913154 | 0.020727349 | -0.401426033 | 0.44467005 | -0.545062416 | -1.932382603 | 0.86188359 |
|  |  | 97.5 | -0.43326295 | -3.462106039 | 2.690609044 | 0.066361186 | -0.928009085 | 1.070711799 | -0.436831993 | -3.741117964 | 2.980880454 |
| **Low-middle SDI** | **longitudinal age curve** | 2.5 | 4415.938343 | 4268.701101 | 4568.254133 | 29233.48501 | 29149.48811 | 29317.72395 | 80.01721629 | 77.27269406 | 82.85921671 |
|  |  | 7.5 | 4459.334739 | 4313.021169 | 4610.611803 | 14579.30437 | 14537.4031 | 14621.32641 | 92.47075061 | 89.36732932 | 95.6819431 |
|  |  | 12.5 | 3470.525283 | 3357.260769 | 3587.611022 | 5465.667086 | 5449.883881 | 5481.496001 | 76.51962539 | 73.97185758 | 79.15514441 |
|  |  | 17.5 | 3400.26134 | 3291.105263 | 3513.037796 | 3151.952563 | 3142.828997 | 3161.102615 | 72.90379647 | 70.51227672 | 75.37642787 |
|  |  | 22.5 | 2934.354134 | 2841.621968 | 3030.112478 | 1830.150037 | 1824.770084 | 1835.545851 | 61.84126215 | 59.84179506 | 63.90753654 |
|  |  | 27.5 | 2220.753215 | 2150.865721 | 2292.911543 | 1503.040623 | 1498.616004 | 1507.478305 | 46.56529486 | 45.0656091 | 48.11488691 |
|  |  | 32.5 | 1787.514828 | 1731.378359 | 1845.471409 | 1496.381539 | 1492.051248 | 1500.724398 | 37.17898449 | 35.98316391 | 38.41454551 |
|  |  | 37.5 | 1636.087795 | 1588.715413 | 1684.872729 | 1811.422347 | 1806.733305 | 1816.123559 | 33.75466618 | 32.75271629 | 34.78726706 |
|  |  | 42.5 | 1611.937515 | 1565.811029 | 1659.422819 | 1779.624316 | 1775.062221 | 1784.198135 | 33.16795502 | 32.19432047 | 34.17103465 |
|  |  | 47.5 | 1642.240675 | 1594.984485 | 1690.896972 | 1402.726922 | 1398.860349 | 1406.604183 | 33.77396186 | 32.77664845 | 34.80162108 |
|  |  | 52.5 | 1651.812505 | 1602.373754 | 1702.776612 | 1298.177691 | 1294.320448 | 1302.04643 | 33.78758929 | 32.74820206 | 34.85996538 |
|  |  | 57.5 | 1445.927079 | 1397.842852 | 1495.665349 | 1469.125618 | 1464.580013 | 1473.685331 | 29.25380093 | 28.25032047 | 30.29292605 |
|  |  | 62.5 | 1194.719211 | 1148.71996 | 1242.560453 | 1451.574696 | 1446.593158 | 1456.573389 | 23.93247788 | 22.97840793 | 24.92616109 |
|  |  | 67.5 | 969.7560475 | 922.3361537 | 1019.613931 | 1241.231607 | 1235.775971 | 1246.711327 | 19.20011384 | 18.22460596 | 20.2278377 |
|  |  | 72.5 | 821.1595667 | 771.8865923 | 873.5778554 | 1094.378598 | 1088.488129 | 1100.300944 | 16.02844443 | 15.02331344 | 17.10082346 |
|  |  | 77.5 | 732.1016089 | 676.7344932 | 791.998592 | 1011.275408 | 1004.422823 | 1018.174744 | 14.0596389 | 12.94071144 | 15.27531519 |
|  |  | 82.5 | 694.8188192 | 625.5183366 | 771.7970253 | 919.8102197 | 911.3289907 | 928.3703789 | 13.15934239 | 11.76920069 | 14.71368334 |
|  |  | 87.5 | 717.3962418 | 616.6815557 | 834.5593653 | 820.6467593 | 809.0231319 | 832.4373889 | 13.40210337 | 11.39706424 | 15.75988087 |
|  |  | 92.5 | 762.975988 | 597.3674122 | 974.4963424 | 830.8173475 | 811.1554523 | 850.9558347 | 14.04297481 | 10.77952979 | 18.29441035 |
|  |  | 97.5 | 782.0271303 | 505.9662199 | 1208.710006 | 945.5904689 | 907.3447479 | 985.448295 | 14.13527685 | 8.787314102 | 22.73801179 |
|  | **period rate ratio** | 1994.5 | 1.052958668 | 1.032213755 | 1.074120501 | 1.000890875 | 0.999231685 | 1.00255282 | 1.054961426 | 1.033046035 | 1.077341737 |
|  |  | 1999.5 | 1.024134812 | 1.008649766 | 1.039857589 | 1.000389215 | 0.999349776 | 1.001429736 | 1.023457325 | 1.007393484 | 1.039777318 |
|  |  | 2004.5 | 1 | 1 | 1 | 1 | 1 | 1 | 1 | 1 | 1 |
|  |  | 2009.5 | 0.967993835 | 0.953552646 | 0.982653731 | 1.000118437 | 0.999086377 | 1.001151563 | 0.969142581 | 0.954138498 | 0.984382608 |
|  |  | 2014.5 | 0.921267775 | 0.903016223 | 0.939888223 | 1.000853046 | 0.999199536 | 1.002509293 | 0.922645905 | 0.903374702 | 0.942328209 |
|  |  | 2019.5 | 0.873996295 | 0.851207125 | 0.897395593 | 1.00336819 | 1.001016808 | 1.005725095 | 0.873404446 | 0.849186432 | 0.898313136 |
|  | **cohort rate ratio** | 1897 | 1.365228518 | 0.300238991 | 6.207884257 | 0.979589476 | 0.845353123 | 1.135141654 | 1.362896331 | 0.260009045 | 7.143929982 |
|  |  | 1902 | 1.349978671 | 0.708599532 | 2.571893336 | 0.985555777 | 0.923903806 | 1.051321776 | 1.349796109 | 0.670541156 | 2.717133048 |
|  |  | 1907 | 1.332914178 | 0.947878964 | 1.874353451 | 0.996321181 | 0.963560555 | 1.030195655 | 1.331283631 | 0.921700709 | 1.922875929 |
|  |  | 1912 | 1.313775301 | 1.057494963 | 1.632164313 | 0.998817128 | 0.978855591 | 1.019185735 | 1.312744993 | 1.040406211 | 1.656371712 |
|  |  | 1917 | 1.290026645 | 1.110221281 | 1.498952302 | 0.998964992 | 0.985574029 | 1.012537897 | 1.28952203 | 1.099124207 | 1.512901869 |
|  |  | 1922 | 1.26591689 | 1.135529827 | 1.41127563 | 1.000145764 | 0.990525727 | 1.009859232 | 1.265610573 | 1.128276537 | 1.419660935 |
|  |  | 1927 | 1.240751903 | 1.143630918 | 1.346120728 | 1.000334229 | 0.993115165 | 1.007605769 | 1.240970885 | 1.139240744 | 1.351785166 |
|  |  | 1932 | 1.209717256 | 1.135559629 | 1.288717742 | 1.00215376 | 0.996521495 | 1.007817858 | 1.209776104 | 1.132511423 | 1.292312105 |
|  |  | 1937 | 1.167894573 | 1.109265695 | 1.229622208 | 1.003508158 | 0.998825935 | 1.008212329 | 1.167914878 | 1.107097687 | 1.232072993 |
|  |  | 1942 | 1.124765639 | 1.076763413 | 1.174907809 | 1.002730374 | 0.998630931 | 1.006846645 | 1.125062818 | 1.075452651 | 1.17696148 |
|  |  | 1947 | 1.083251017 | 1.042786225 | 1.125286025 | 1.002610911 | 0.999005411 | 1.006229425 | 1.083499794 | 1.041794584 | 1.126874552 |
|  |  | 1952 | 1.040976907 | 1.00630733 | 1.076840929 | 1.002036651 | 0.998901165 | 1.00518198 | 1.041041142 | 1.005371711 | 1.077976082 |
|  |  | 1957 | 1 | 1 | 1 | 1 | 1 | 1 | 1 | 1 | 1 |
|  |  | 1962 | 0.95904869 | 0.930385037 | 0.988595423 | 0.999747726 | 0.996974072 | 1.002529096 | 0.958545252 | 0.929139939 | 0.988881179 |
|  |  | 1967 | 0.917641057 | 0.890278598 | 0.945844494 | 1.000588733 | 0.997817438 | 1.003367725 | 0.916701283 | 0.888673141 | 0.945613414 |
|  |  | 1972 | 0.87352251 | 0.847019954 | 0.900854308 | 1.00211431 | 0.999324867 | 1.00491154 | 0.872235251 | 0.845119699 | 0.900220802 |
|  |  | 1977 | 0.814633604 | 0.789344166 | 0.840733279 | 1.00191126 | 0.999094577 | 1.004735885 | 0.813652965 | 0.787792185 | 0.840362675 |
|  |  | 1982 | 0.767842004 | 0.743537238 | 0.792941246 | 1.001855203 | 0.998991421 | 1.004727195 | 0.766938629 | 0.742109233 | 0.792598764 |
|  |  | 1987 | 0.735316207 | 0.711681292 | 0.759736037 | 1.002971468 | 1.000083341 | 1.005867936 | 0.734216876 | 0.710088234 | 0.759165404 |
|  |  | 1992 | 0.717562531 | 0.694280982 | 0.741624787 | 1.005525822 | 1.002634764 | 1.008425216 | 0.715466099 | 0.691719896 | 0.740027491 |
|  |  | 1997 | 0.709403742 | 0.686002184 | 0.733603597 | 1.007932194 | 1.005007228 | 1.010865674 | 0.707084667 | 0.683230934 | 0.731771209 |
|  |  | 2002 | 0.710132624 | 0.686264995 | 0.734830347 | 1.008118385 | 1.005180393 | 1.011064966 | 0.707658922 | 0.683336312 | 0.732847269 |
|  |  | 2007 | 0.708218112 | 0.68382639 | 0.733479874 | 1.006244985 | 1.003311358 | 1.009187189 | 0.705704306 | 0.680820944 | 0.731497132 |
|  |  | 2012 | 0.710395385 | 0.685017971 | 0.736712939 | 1.000737198 | 0.99781948 | 1.003663446 | 0.707961614 | 0.681932581 | 0.734984162 |
|  |  | 2017 | 0.715619785 | 0.687023459 | 0.745406391 | 0.99365463 | 0.990708386 | 0.996609636 | 0.714115234 | 0.684245025 | 0.745289405 |
|  | **local drifts** | 2.5 | -0.006893748 | -0.095023237 | 0.081313483 | -0.0472648 | -0.05019296 | -0.044336554 | -0.004855256 | -0.09988311 | 0.09026299 |
|  |  | 7.5 | -0.120322775 | -0.190923128 | -0.049672482 | -0.005040506 | -0.008227464 | -0.001853446 | -0.127047672 | -0.200295519 | -0.053746065 |
|  |  | 12.5 | -0.29678231 | -0.367667764 | -0.225846422 | 0.022634752 | 0.01806534 | 0.027204373 | -0.307163172 | -0.379021965 | -0.235252547 |
|  |  | 17.5 | -0.540449451 | -0.616281283 | -0.464559758 | 0.029471003 | 0.023428082 | 0.035514289 | -0.551330515 | -0.627959576 | -0.474642362 |
|  |  | 22.5 | -0.833344745 | -0.916842369 | -0.749776757 | 0.023351988 | 0.0158942 | 0.030810333 | -0.841550563 | -0.926226802 | -0.756801953 |
|  |  | 27.5 | -1.026458649 | -1.119917116 | -0.932911847 | 0.015497917 | 0.006877946 | 0.024118631 | -1.031855769 | -1.126990408 | -0.936629593 |
|  |  | 32.5 | -1.098306111 | -1.203230087 | -0.993270703 | 0.011251475 | 0.0017071 | 0.020796761 | -1.101156085 | -1.208239809 | -0.993956288 |
|  |  | 37.5 | -1.057081241 | -1.173194666 | -0.940831392 | 0.009872583 | -0.000515613 | 0.020261858 | -1.061815915 | -1.180608483 | -0.942880544 |
|  |  | 42.5 | -0.952962751 | -1.079894466 | -0.825868161 | 0.003743715 | -0.007829953 | 0.015318724 | -0.959332571 | -1.089489745 | -0.829004123 |
|  |  | 47.5 | -0.851258765 | -0.991021495 | -0.711298744 | -0.004038502 | -0.017349835 | 0.009274604 | -0.858230612 | -1.001872476 | -0.71438033 |
|  |  | 52.5 | -0.809912893 | -0.966241271 | -0.653337746 | -0.012173254 | -0.027261577 | 0.002917346 | -0.814883573 | -0.976065614 | -0.653439176 |
|  |  | 57.5 | -0.784116186 | -0.964286844 | -0.603617753 | -0.015727054 | -0.032584016 | 0.001132749 | -0.786198427 | -0.972817358 | -0.599227809 |
|  |  | 62.5 | -0.759767808 | -0.975608462 | -0.543456693 | -0.008730291 | -0.028218348 | 0.010761565 | -0.75984993 | -0.984612354 | -0.534577301 |
|  |  | 67.5 | -0.709855714 | -0.981106734 | -0.437861633 | 0.005197201 | -0.018915808 | 0.029316026 | -0.709733221 | -0.993954487 | -0.424696027 |
|  |  | 72.5 | -0.631575659 | -0.988465041 | -0.27339986 | 0.011907447 | -0.019778066 | 0.043603002 | -0.630104986 | -1.006711597 | -0.252065627 |
|  |  | 77.5 | -0.542837843 | -1.0301033 | -0.053173397 | 0.017542712 | -0.026185358 | 0.061289907 | -0.540605749 | -1.058533663 | -0.019966642 |
|  |  | 82.5 | -0.456906799 | -1.152679226 | 0.243763071 | 0.018960274 | -0.045553089 | 0.083515276 | -0.453637308 | -1.198248201 | 0.296585282 |
|  |  | 87.5 | -0.385161019 | -1.457857668 | 0.699212645 | 0.01995614 | -0.085034344 | 0.125056948 | -0.379812563 | -1.535317432 | 0.789252412 |
|  |  | 92.5 | -0.339315077 | -2.296266345 | 1.656832833 | 0.049189661 | -0.148776864 | 0.247548677 | -0.336525665 | -2.457421261 | 1.830485158 |
|  |  | 97.5 | -0.301468258 | -4.714176867 | 4.315593911 | 0.083967414 | -0.356967322 | 0.52685335 | -0.297474439 | -5.111485085 | 4.760767014 |
| **Low SDI** | **longitudinal age curve** | 2.5 | 3782.454854 | 3691.15649 | 3876.011423 | 29664.32436 | 29594.60632 | 29734.20664 | 67.63643383 | 66.00784596 | 69.30520325 |
|  |  | 7.5 | 3829.881851 | 3738.53526 | 3923.460385 | 15345.9152 | 15309.83677 | 15382.07866 | 78.9223477 | 77.05299675 | 80.83705021 |
|  |  | 12.5 | 2922.25788 | 2852.653838 | 2993.560243 | 5613.175918 | 5599.924791 | 5626.458401 | 64.48388088 | 62.96256828 | 66.04195169 |
|  |  | 17.5 | 2893.340667 | 2825.156254 | 2963.170694 | 3188.577623 | 3181.024978 | 3196.148199 | 62.12351356 | 60.67173506 | 63.61003082 |
|  |  | 22.5 | 3037.864654 | 2967.62205 | 3109.769876 | 1824.514318 | 1820.124446 | 1828.914777 | 63.74064324 | 62.27682031 | 65.23887348 |
|  |  | 27.5 | 2390.134363 | 2334.979675 | 2446.591864 | 1523.45776 | 1519.778021 | 1527.146408 | 49.91245304 | 48.76816047 | 51.08359521 |
|  |  | 32.5 | 1981.333034 | 1935.586288 | 2028.160985 | 1527.870436 | 1524.221413 | 1531.528195 | 41.07439099 | 40.13156374 | 42.03936848 |
|  |  | 37.5 | 1843.044117 | 1803.299289 | 1883.664923 | 1838.966224 | 1834.987877 | 1842.953197 | 37.9371322 | 37.12304452 | 38.76907237 |
|  |  | 42.5 | 1840.54627 | 1801.197309 | 1880.754848 | 1799.958013 | 1796.076847 | 1803.847566 | 37.83776406 | 37.03241332 | 38.66062891 |
|  |  | 47.5 | 1896.565037 | 1855.499267 | 1938.539671 | 1411.678443 | 1408.379212 | 1414.985402 | 39.01000357 | 38.16897364 | 39.86956507 |
|  |  | 52.5 | 1932.706973 | 1888.891576 | 1977.538728 | 1304.798648 | 1301.481751 | 1308.123999 | 39.58418835 | 38.68946795 | 40.49959978 |
|  |  | 57.5 | 1734.952743 | 1691.035385 | 1780.010665 | 1480.298979 | 1476.346079 | 1484.262463 | 35.21080102 | 34.31932224 | 36.12543685 |
|  |  | 62.5 | 1459.91376 | 1416.65428 | 1504.494227 | 1461.167517 | 1456.787472 | 1465.560732 | 29.35293984 | 28.47969086 | 30.25296454 |
|  |  | 67.5 | 1190.497808 | 1145.145688 | 1237.646044 | 1245.831241 | 1241.026327 | 1250.65476 | 23.67923 | 22.77010786 | 24.62464986 |
|  |  | 72.5 | 1009.385969 | 961.356862 | 1059.814596 | 1100.083663 | 1094.832335 | 1105.360178 | 19.82263966 | 18.86638114 | 20.82736696 |
|  |  | 77.5 | 909.1979896 | 853.5475923 | 968.4767338 | 1023.24781 | 1017.01053 | 1029.523343 | 17.56351371 | 16.46520685 | 18.73508281 |
|  |  | 82.5 | 875.2012092 | 802.3887181 | 954.6210451 | 930.5751058 | 922.6327422 | 938.5858402 | 16.67170308 | 15.24423065 | 18.23284427 |
|  |  | 87.5 | 908.6690803 | 797.0383638 | 1035.934448 | 821.1958407 | 809.8754434 | 832.6744739 | 17.10785236 | 14.93094382 | 19.6021508 |
|  |  | 92.5 | 968.3210272 | 770.3666329 | 1217.142036 | 819.1465814 | 798.73789 | 840.0767389 | 17.95356698 | 14.12704451 | 22.81656061 |
|  |  | 97.5 | 980.8384118 | 612.4582707 | 1570.791083 | 925.8639251 | 880.7909354 | 973.2434491 | 17.81754591 | 10.81419467 | 29.35631843 |
|  | **period rate ratio** | 1994.5 | 1.006719023 | 0.989850182 | 1.023875339 | 1.002069513 | 1.00044805 | 1.003693604 | 1.005773058 | 0.988368067 | 1.023484549 |
|  |  | 1999.5 | 1.007186139 | 0.995559128 | 1.018948941 | 1.000724039 | 0.999807091 | 1.001641828 | 1.005844501 | 0.994046821 | 1.0177822 |
|  |  | 2004.5 | 1 | 1 | 1 | 1 | 1 | 1 | 1 | 1 | 1 |
|  |  | 2009.5 | 0.97727639 | 0.966326004 | 0.988350866 | 0.999788442 | 0.998885845 | 1.000691854 | 0.978385914 | 0.967241227 | 0.989659013 |
|  |  | 2014.5 | 0.944253141 | 0.928573305 | 0.960197746 | 1.00050805 | 0.998898791 | 1.002119901 | 0.946424635 | 0.930184485 | 0.962948323 |
|  |  | 2019.5 | 0.909217986 | 0.888310586 | 0.930617466 | 1.002568882 | 1.000208911 | 1.004934421 | 0.90978291 | 0.888026455 | 0.932072393 |
|  | **cohort rate ratio** | 1897 | 1.161076211 | 0.242909305 | 5.549799613 | 0.983125705 | 0.832279093 | 1.161312544 | 1.150822465 | 0.218283417 | 6.067306262 |
|  |  | 1902 | 1.154949427 | 0.608293667 | 2.19286876 | 0.986138977 | 0.918512513 | 1.058744512 | 1.14519968 | 0.582780259 | 2.25038904 |
|  |  | 1907 | 1.148876425 | 0.835271014 | 1.580226079 | 0.993635314 | 0.959894529 | 1.028562105 | 1.13869435 | 0.815799341 | 1.589391849 |
|  |  | 1912 | 1.14313596 | 0.94755174 | 1.379090731 | 0.995233028 | 0.976306747 | 1.014526207 | 1.134449173 | 0.933474635 | 1.378692979 |
|  |  | 1917 | 1.136383602 | 1.005494413 | 1.284311155 | 0.996190078 | 0.9842049 | 1.008321206 | 1.129718475 | 0.995739544 | 1.281724565 |
|  |  | 1922 | 1.127244244 | 1.033471426 | 1.229525611 | 0.995495001 | 0.987026586 | 1.004036072 | 1.121929529 | 1.02646747 | 1.226269614 |
|  |  | 1927 | 1.116915592 | 1.046684472 | 1.191859125 | 0.995544859 | 0.989171177 | 1.001959609 | 1.112463327 | 1.041355396 | 1.188426793 |
|  |  | 1932 | 1.102179124 | 1.048667659 | 1.158421176 | 0.998665799 | 0.993727874 | 1.003628262 | 1.097860818 | 1.043965135 | 1.154538916 |
|  |  | 1937 | 1.083498683 | 1.040985772 | 1.127747783 | 1.001259201 | 0.99717999 | 1.0053551 | 1.080148301 | 1.037494329 | 1.124555884 |
|  |  | 1942 | 1.064140492 | 1.029072853 | 1.100403128 | 1.001180982 | 0.997634839 | 1.004739729 | 1.061997508 | 1.026915724 | 1.098277766 |
|  |  | 1947 | 1.044894133 | 1.014940911 | 1.075731343 | 1.00028599 | 0.99718019 | 1.003401462 | 1.043602204 | 1.013693713 | 1.074393128 |
|  |  | 1952 | 1.023493301 | 0.997523829 | 1.050138862 | 1.000964864 | 0.998279342 | 1.003657612 | 1.022930854 | 0.99702429 | 1.049510572 |
|  |  | 1957 | 1 | 1 | 1 | 1 | 1 | 1 | 1 | 1 | 1 |
|  |  | 1962 | 0.975109385 | 0.953119056 | 0.997607074 | 0.999555702 | 0.997202399 | 1.001914558 | 0.975203912 | 0.953299405 | 0.997611731 |
|  |  | 1967 | 0.950274274 | 0.929105314 | 0.971925552 | 0.99859785 | 0.996274325 | 1.000926794 | 0.950722381 | 0.929647549 | 0.972274973 |
|  |  | 1972 | 0.925827697 | 0.905081392 | 0.94704955 | 0.997533145 | 0.995226082 | 0.999845557 | 0.926859999 | 0.90620504 | 0.947985743 |
|  |  | 1977 | 0.893715428 | 0.873353284 | 0.914552312 | 0.997604397 | 0.9952911 | 0.999923072 | 0.895666836 | 0.875386197 | 0.91641733 |
|  |  | 1982 | 0.859446909 | 0.839529385 | 0.879836969 | 0.997770206 | 0.995426689 | 1.00011924 | 0.861919991 | 0.842086194 | 0.882220937 |
|  |  | 1987 | 0.830348081 | 0.810836865 | 0.850328796 | 0.996614245 | 0.994261806 | 0.998972249 | 0.832826541 | 0.813403723 | 0.852713147 |
|  |  | 1992 | 0.812567277 | 0.793349437 | 0.832250644 | 0.997566363 | 0.995217576 | 0.999920694 | 0.814092561 | 0.794978167 | 0.83366654 |
|  |  | 1997 | 0.802068701 | 0.782901214 | 0.821705458 | 0.997810305 | 0.995448381 | 1.000177835 | 0.803885759 | 0.784815713 | 0.823419184 |
|  |  | 2002 | 0.801681362 | 0.78226012 | 0.821584778 | 0.996825852 | 0.994463054 | 0.999194264 | 0.803769347 | 0.784438854 | 0.823576189 |
|  |  | 2007 | 0.802113116 | 0.782365905 | 0.822358755 | 0.994631722 | 0.99227596 | 0.996993076 | 0.804548412 | 0.784864405 | 0.824726085 |
|  |  | 2012 | 0.808130915 | 0.787714903 | 0.829076071 | 0.99075049 | 0.98840404 | 0.993102511 | 0.811546256 | 0.7910909 | 0.83253053 |
|  |  | 2017 | 0.816574496 | 0.79426705 | 0.839508459 | 0.985605667 | 0.983247126 | 0.987969866 | 0.822139512 | 0.79945289 | 0.845469927 |
|  | **local drifts** | 2.5 | 0.027275142 | -0.025976965 | 0.080555615 | -0.04788372 | -0.049747384 | -0.046020022 | 0.044925294 | -0.010778921 | 0.100660541 |
|  |  | 7.5 | -0.099912825 | -0.14502519 | -0.054780079 | -0.0224722 | -0.024612314 | -0.020332041 | -0.094209089 | -0.139590363 | -0.048807191 |
|  |  | 12.5 | -0.264565291 | -0.311719086 | -0.217389192 | -0.008497272 | -0.011743241 | -0.005251197 | -0.26454236 | -0.310873392 | -0.218189796 |
|  |  | 17.5 | -0.440348504 | -0.49191296 | -0.388757327 | -0.001616064 | -0.006084089 | 0.00285216 | -0.440824347 | -0.491333385 | -0.390289671 |
|  |  | 22.5 | -0.591091599 | -0.648411025 | -0.533739102 | 6.60E-05 | -0.005641861 | 0.005774119 | -0.588289716 | -0.644654419 | -0.531893037 |
|  |  | 27.5 | -0.654073011 | -0.719501805 | -0.588601097 | -0.004437583 | -0.011208237 | 0.002333529 | -0.646516808 | -0.711131457 | -0.581860108 |
|  |  | 32.5 | -0.649429951 | -0.724228326 | -0.574575221 | -0.009800399 | -0.017441421 | -0.002158793 | -0.63654767 | -0.710645264 | -0.562394778 |
|  |  | 37.5 | -0.595295548 | -0.679532745 | -0.510986906 | -0.010336852 | -0.018804519 | -0.001868469 | -0.583217197 | -0.666860606 | -0.499503356 |
|  |  | 42.5 | -0.532831192 | -0.626535257 | -0.43903877 | -0.01438915 | -0.023998483 | -0.004778893 | -0.522956972 | -0.616184738 | -0.429641752 |
|  |  | 47.5 | -0.486127572 | -0.591299344 | -0.380844531 | -0.012185688 | -0.023453758 | -0.000916349 | -0.477643979 | -0.582499437 | -0.372677929 |
|  |  | 52.5 | -0.454077608 | -0.57353412 | -0.334477574 | -0.009183883 | -0.022127774 | 0.003761684 | -0.444413496 | -0.563865415 | -0.324818081 |
|  |  | 57.5 | -0.418664585 | -0.558301736 | -0.278831355 | -0.006500609 | -0.021131373 | 0.008132296 | -0.405744 | -0.545988901 | -0.265301332 |
|  |  | 62.5 | -0.385323886 | -0.554892862 | -0.215465769 | 0.00279952 | -0.014285614 | 0.019887572 | -0.36935644 | -0.54056852 | -0.197849631 |
|  |  | 67.5 | -0.350752427 | -0.566790166 | -0.134245305 | 0.018248788 | -0.003087402 | 0.039589531 | -0.335736127 | -0.555181445 | -0.115806558 |
|  |  | 72.5 | -0.309010888 | -0.594974293 | -0.022224839 | 0.024880392 | -0.003154838 | 0.052923482 | -0.295219861 | -0.587652371 | -0.001927128 |
|  |  | 77.5 | -0.262763378 | -0.65993495 | 0.135996126 | 0.025968044 | -0.013166715 | 0.06511812 | -0.248919729 | -0.658127877 | 0.161974025 |
|  |  | 82.5 | -0.210515296 | -0.807397936 | 0.389959032 | 0.021533917 | -0.03920485 | 0.08230959 | -0.193830347 | -0.813354758 | 0.429563642 |
|  |  | 87.5 | -0.162818245 | -1.154462364 | 0.838774305 | 0.014567647 | -0.092452496 | 0.121702429 | -0.14174142 | -1.177972031 | 0.905354925 |
|  |  | 92.5 | -0.131557929 | -2.065203656 | 1.840266115 | 0.030882006 | -0.184936829 | 0.247167482 | -0.110616996 | -2.14547261 | 1.966552832 |
|  |  | 97.5 | -0.1150669 | -4.666903999 | 4.654105225 | 0.054040065 | -0.443179236 | 0.553742642 | -0.098066803 | -4.925575956 | 4.974564473 |

| **Table S7. Age-Standardized Rates and Frontier Analysis Results for Otitis Media by Country, 2021** | | | | | | | | | | | | | |
| --- | --- | --- | --- | --- | --- | --- | --- | --- | --- | --- | --- | --- | --- |
| **location** | **SDI** | **Prevalence** | | | | **Incidence** | | | | **YLDs** | | | |
|  |  | **ASR** | **Effective difference** | **frontier** | **Effective difference rank (ASR rank)** | **ASR** | **Effective difference** | **frontier** | **Effective difference rank (ASR rank)** | **ASR** | **Effective difference** | **frontier** | **Effective difference rank (ASR rank)** |
| Afghanistan | 0.337199998 | 2049.877494 | 529.0804463 | 1520.797048 | 48 (17) | 5176.796486 | 726.3485853 | 4450.4479 | 185 (105) | 41.97074364 | 12.13831292 | 29.83243072 | 39 (17) |
| Albania | 0.706849791 | 1346.311477 | 479.7203736 | 866.5911033 | 61 (117) | 4004.163339 | 785.0347065 | 3219.128632 | 174 (193) | 27.12759805 | 10.73883102 | 16.38876703 | 60 (112) |
| Algeria | 0.659500924 | 1460.820142 | 377.7615087 | 1083.058633 | 118 (91) | 5177.038896 | 1402.693555 | 3774.345341 | 94 (103) | 29.45715219 | 8.710183531 | 20.74696866 | 106 (86) |
| American Samoa | 0.723727533 | 1265.663183 | 398.5144569 | 867.1487264 | 106 (133) | 4602.346362 | 1383.228282 | 3219.118079 | 116 (146) | 24.81910804 | 8.42825832 | 16.39084972 | 111 (137) |
| Andorra | 0.869444113 | 941.9052084 | 200.1851032 | 741.7201052 | 186 (194) | 5316.097483 | 2096.980288 | 3219.117195 | 22 (68) | 17.2524818 | 3.765048322 | 13.48743347 | 196 (197) |
| Angola | 0.453721949 | 1542.89045 | 255.3218672 | 1287.568583 | 167 (73) | 5816.040081 | 1365.60436 | 4450.435722 | 127 (33) | 30.90700469 | 4.981802702 | 25.92520199 | 172 (73) |
| Antigua and Barbuda | 0.749886887 | 1292.437037 | 448.017471 | 844.419566 | 74 (128) | 5183.791092 | 1964.674107 | 3219.116986 | 49 (83) | 25.20427799 | 9.545677416 | 15.65860057 | 83 (133) |
| Argentina | 0.723122973 | 1092.882803 | 225.752535 | 867.1302679 | 178 (169) | 4955.491236 | 1736.372115 | 3219.119121 | 79 (133) | 20.81504072 | 4.44663696 | 16.36840376 | 182 (168) |
| Armenia | 0.701833194 | 1408.361988 | 540.5780084 | 867.7839795 | 44 (105) | 4381.228428 | 1162.093105 | 3219.135324 | 147 (174) | 28.33798266 | 11.959254 | 16.37872866 | 42 (99) |
| Australia | 0.844252814 | 793.2057635 | 51.74747686 | 741.4582867 | 203 (203) | 4967.12095 | 1748.003502 | 3219.117449 | 77 (131) | 14.53367373 | 1.06300431 | 13.47066942 | 203 (203) |
| Austria | 0.853837004 | 940.023389 | 198.4278225 | 741.5955665 | 187 (195) | 5313.021842 | 2093.903884 | 3219.117957 | 24 (70) | 17.25985876 | 3.789145752 | 13.47071301 | 195 (196) |
| Azerbaijan | 0.694851274 | 1288.928602 | 422.0629915 | 866.8656106 | 88 (130) | 4380.116555 | 1158.358681 | 3221.757874 | 151 (176) | 25.77280334 | 9.389546188 | 16.38325716 | 87 (128) |
| Bahamas | 0.805020668 | 1244.231323 | 502.8986888 | 741.3326342 | 58 (141) | 5183.655269 | 1964.538035 | 3219.117234 | 50 (84) | 24.14470817 | 10.63654045 | 13.50816772 | 62 (145) |
| Bahrain | 0.753043204 | 1230.586825 | 390.270077 | 840.316748 | 109 (144) | 5154.708176 | 1935.590471 | 3219.117705 | 65 (127) | 24.3404403 | 8.751097806 | 15.58934249 | 103 (144) |
| Bangladesh | 0.492420885 | 2154.899261 | 876.1807966 | 1278.718464 | 2 (10) | 6223.391588 | 2040.09835 | 4183.293238 | 33 (11) | 43.87104965 | 18.36600151 | 25.50504814 | 3 (11) |
| Barbados | 0.746748764 | 1383.827366 | 537.0331215 | 846.7942445 | 45 (111) | 5189.949941 | 1970.832562 | 3219.117379 | 46 (79) | 27.07112741 | 11.37214993 | 15.69897748 | 52 (113) |
| Belarus | 0.784484711 | 1344.669129 | 562.1211303 | 782.5479985 | 37 (118) | 4379.755678 | 1160.638758 | 3219.11692 | 148 (177) | 27.02709483 | 12.62927787 | 14.39781696 | 37 (115) |
| Belgium | 0.853654016 | 1024.493074 | 282.5591439 | 741.9339305 | 162 (183) | 5317.952077 | 2098.835247 | 3219.11683 | 20 (66) | 18.99422464 | 5.523741835 | 13.47048281 | 167 (183) |
| Belize | 0.610229002 | 1517.192449 | 344.8341807 | 1172.358268 | 134 (80) | 5178.732552 | 1390.453947 | 3788.278605 | 101 (95) | 29.96643643 | 7.629626399 | 22.33681003 | 124 (81) |
| Benin | 0.373486574 | 2006.684734 | 493.7061522 | 1512.978582 | 60 (21) | 5815.721693 | 1365.277991 | 4450.443702 | 128 (35) | 41.73807528 | 11.88958811 | 29.84848717 | 44 (19) |
| Bermuda | 0.821365422 | 1121.855971 | 380.0207565 | 741.8352146 | 117 (164) | 5185.138385 | 1966.020563 | 3219.117822 | 48 (82) | 21.59765531 | 8.123042954 | 13.47461236 | 118 (164) |
| Bhutan | 0.473062378 | 1847.014126 | 553.1136621 | 1293.900463 | 41 (34) | 6229.637615 | 2019.213697 | 4210.423917 | 43 (10) | 37.21300662 | 11.51330566 | 25.69970096 | 50 (33) |
| Bolivia (Plurinational State of) | 0.599010799 | 1540.760155 | 357.2776651 | 1183.48249 | 126 (74) | 5180.987978 | 1392.634446 | 3788.353531 | 99 (88) | 29.92937119 | 7.139581228 | 22.78978996 | 137 (82) |
| Bosnia and Herzegovina | 0.723077893 | 1343.225222 | 475.9579091 | 867.2673132 | 64 (120) | 3996.961958 | 777.8413521 | 3219.120606 | 181 (200) | 27.000717 | 10.61752661 | 16.38319039 | 63 (116) |
| Botswana | 0.642721629 | 1438.096913 | 306.0586088 | 1132.038304 | 152 (98) | 5814.615129 | 2026.314842 | 3788.300287 | 39 (41) | 27.90126093 | 6.444835416 | 21.45642551 | 149 (105) |
| Brazil | 0.653043887 | 1424.961486 | 338.9934817 | 1085.968004 | 135 (101) | 5675.500235 | 1887.203261 | 3788.296973 | 72 (57) | 28.17612168 | 7.360545587 | 20.81557609 | 132 (103) |
| Brunei Darussalam | 0.810234367 | 876.736112 | 134.8343789 | 741.901733 | 200 (200) | 4004.778609 | 785.6598027 | 3219.118806 | 173 (192) | 16.5404294 | 3.079318238 | 13.46111116 | 198 (199) |
| Bulgaria | 0.768150939 | 1231.33187 | 415.4847926 | 815.8470773 | 91 (143) | 4003.322475 | 784.2054692 | 3219.117006 | 176 (195) | 24.62160579 | 9.633889827 | 14.98771596 | 78 (141) |
| Burkina Faso | 0.285118402 | 2129.228279 | 609.2709821 | 1519.957297 | 30 (12) | 5816.693833 | 1365.89175 | 4450.802083 | 125 (30) | 44.5270646 | 14.69210655 | 29.83495804 | 17 (10) |
| Burundi | 0.289374365 | 2156.933623 | 636.1929352 | 1520.740688 | 21 (9) | 6117.022696 | 1666.590974 | 4450.431722 | 89 (23) | 43.81909297 | 13.97120949 | 29.84788348 | 23 (12) |
| Cabo Verde | 0.533534539 | 1682.235306 | 431.7931502 | 1250.442155 | 84 (51) | 5807.243129 | 2016.962526 | 3790.280603 | 44 (52) | 34.65555424 | 10.12768822 | 24.52786602 | 70 (48) |
| Cambodia | 0.473621491 | 1609.906268 | 324.83605 | 1285.070218 | 145 (58) | 4455.889631 | 251.0508288 | 4204.838802 | 197 (158) | 32.91873406 | 7.256055118 | 25.66267894 | 134 (55) |
| Cameroon | 0.479691223 | 1938.836992 | 664.172993 | 1274.663999 | 17 (25) | 5814.221371 | 1622.666611 | 4191.55476 | 90 (43) | 40.3098485 | 14.81109688 | 25.49875163 | 15 (22) |
| Canada | 0.87317068 | 921.9505841 | 180.7557513 | 741.1948328 | 190 (197) | 4384.762342 | 1165.645468 | 3219.116874 | 143 (168) | 17.33795654 | 3.860617755 | 13.47733879 | 192 (195) |
| Central African Republic | 0.30916769 | 2290.428998 | 770.9084157 | 1519.520583 | 6 (5) | 5816.311243 | 1365.870741 | 4450.440502 | 126 (32) | 47.0127376 | 17.16581663 | 29.84692097 | 5 (4) |
| Chad | 0.240436019 | 2082.73581 | 560.9234747 | 1521.812335 | 38 (15) | 5813.42022 | 636.241038 | 5177.179182 | 189 (46) | 43.41521651 | 13.56177889 | 29.85343762 | 28 (14) |
| Chile | 0.771514716 | 1053.895616 | 246.1879408 | 807.707675 | 173 (178) | 4970.129077 | 1751.011824 | 3219.117254 | 76 (130) | 20.00385176 | 5.112059546 | 14.89179221 | 170 (173) |
| China | 0.72162976 | 1332.108262 | 465.5003774 | 866.6078841 | 69 (121) | 4179.924598 | 960.8032174 | 3219.121381 | 159 (185) | 26.51607932 | 10.11722691 | 16.39885241 | 71 (120) |
| Colombia | 0.655442913 | 1438.155185 | 350.7206653 | 1087.434519 | 130 (97) | 5176.114087 | 1387.80068 | 3788.313408 | 108 (110) | 28.24499893 | 7.371311773 | 20.87368716 | 131 (100) |
| Comoros | 0.475978688 | 1720.676852 | 442.2554765 | 1278.421376 | 78 (48) | 6127.365634 | 1935.386061 | 4191.979573 | 66 (18) | 34.38225024 | 8.876115409 | 25.50613483 | 97 (50) |
| Congo | 0.583075236 | 1603.371822 | 410.2526878 | 1193.119134 | 99 (62) | 5813.612269 | 2025.326948 | 3788.285321 | 40 (45) | 32.19534208 | 8.94821911 | 23.24712298 | 94 (59) |
| Cook Islands | 0.779109955 | 1104.506493 | 301.0488419 | 803.457651 | 154 (167) | 4598.958561 | 1379.841127 | 3219.117433 | 118 (151) | 21.46230445 | 6.688750704 | 14.77355374 | 144 (166) |
| Costa Rica | 0.700340477 | 1408.675552 | 542.4123573 | 866.2631946 | 42 (104) | 5182.290201 | 1957.59594 | 3224.694261 | 54 (86) | 27.58783204 | 11.21104967 | 16.37678237 | 54 (108) |
| Croatia | 0.798341027 | 1194.943378 | 446.5830402 | 748.3603381 | 76 (150) | 4007.539654 | 788.4226496 | 3219.117004 | 171 (190) | 23.87417953 | 10.2556058 | 13.61857373 | 68 (147) |
| Cuba | 0.668729864 | 1519.191487 | 609.0801574 | 910.11133 | 31 (79) | 5175.523378 | 1792.836987 | 3382.686391 | 74 (113) | 30.06411435 | 11.95804004 | 18.10607431 | 43 (79) |
| Cyprus | 0.835630545 | 1058.658149 | 317.0529832 | 741.6051654 | 149 (176) | 5317.094441 | 2097.977257 | 3219.117184 | 21 (67) | 19.68231402 | 6.191475558 | 13.49083846 | 157 (180) |
| Czechia | 0.828450433 | 1125.332043 | 383.8915007 | 741.4405423 | 112 (163) | 3999.577966 | 780.4602492 | 3219.117717 | 177 (196) | 22.38833093 | 8.921914329 | 13.4664166 | 95 (160) |
| Côte d'Ivoire | 0.425941883 | 1862.639804 | 435.4028592 | 1427.236945 | 82 (32) | 5810.872656 | 1360.437513 | 4450.435143 | 134 (50) | 38.54094579 | 9.544296971 | 28.99664882 | 84 (31) |
| Democratic People's Republic of Korea | 0.569854634 | 1776.591358 | 573.7337388 | 1202.857619 | 35 (38) | 3788.207969 | 0 | 3788.207969 | 204 (202) | 35.39443455 | 11.96444106 | 23.42999349 | 41 (42) |
| Democratic Republic of the Congo | 0.383179849 | 2235.341346 | 727.3002772 | 1508.041069 | 10 (6) | 5813.205394 | 1362.783228 | 4450.422165 | 131 (47) | 45.89735051 | 16.02853769 | 29.86881283 | 11 (7) |
| Denmark | 0.896424204 | 996.3860862 | 255.026034 | 741.3600522 | 169 (185) | 5313.02526 | 2093.908186 | 3219.117074 | 23 (69) | 18.13318888 | 4.648741308 | 13.48444757 | 180 (190) |
| Djibouti | 0.487958371 | 1606.335605 | 329.3980646 | 1276.93754 | 142 (60) | 6140.04066 | 1950.636209 | 4189.404451 | 61 (12) | 31.90957125 | 6.34550366 | 25.56406759 | 153 (64) |
| Dominica | 0.746967185 | 1469.006471 | 622.0847247 | 846.9217466 | 26 (90) | 5172.194328 | 1953.076777 | 3219.117551 | 58 (120) | 28.92232146 | 13.24150671 | 15.68081474 | 32 (93) |
| Dominican Republic | 0.619388201 | 1373.473405 | 214.4465951 | 1159.02681 | 181 (114) | 5175.451573 | 1387.196029 | 3788.255545 | 111 (114) | 26.9216979 | 4.78059317 | 22.14110473 | 177 (117) |
| Ecuador | 0.661017053 | 1444.610939 | 368.8215291 | 1075.78941 | 119 (96) | 5181.307883 | 1400.789387 | 3780.518496 | 95 (87) | 28.24181946 | 7.496296243 | 20.74552321 | 127 (101) |
| Egypt | 0.606787094 | 1524.732845 | 348.7582378 | 1175.974607 | 131 (76) | 5174.931526 | 1386.654845 | 3788.276681 | 112 (117) | 30.90011622 | 8.396910586 | 22.50320563 | 113 (74) |
| El Salvador | 0.563775188 | 1565.649813 | 364.0987824 | 1201.551031 | 122 (70) | 5179.119014 | 1390.29039 | 3788.828624 | 102 (92) | 30.915406 | 7.569322198 | 23.3460838 | 125 (72) |
| Equatorial Guinea | 0.657857456 | 1264.707476 | 177.7340416 | 1086.973434 | 192 (134) | 5814.864167 | 2026.529956 | 3788.33421 | 37 (38) | 24.76240627 | 4.013005393 | 20.74940088 | 187 (139) |
| Eritrea | 0.403863943 | 1830.306354 | 390.6544895 | 1439.651865 | 108 (35) | 6132.095955 | 1681.673601 | 4450.422354 | 83 (15) | 36.69918474 | 7.447137695 | 29.25204705 | 129 (35) |
| Estonia | 0.844917787 | 1246.210609 | 504.2503557 | 741.9602533 | 57 (139) | 4378.043891 | 1158.926778 | 3219.117113 | 150 (180) | 24.91738459 | 11.44560672 | 13.47177787 | 51 (135) |
| Eswatini | 0.585459713 | 1574.586498 | 381.6264159 | 1192.960082 | 113 (67) | 5818.45216 | 2030.161861 | 3788.290299 | 34 (24) | 30.71537659 | 7.483215647 | 23.23216095 | 128 (75) |
| Ethiopia | 0.358823295 | 1931.582448 | 412.5389254 | 1519.043523 | 97 (27) | 6639.680393 | 2189.248726 | 4450.431667 | 13 (3) | 38.89665515 | 9.05092054 | 29.84573461 | 91 (27) |
| Fiji | 0.675051631 | 1348.965634 | 455.1016173 | 893.8640164 | 73 (116) | 4605.599762 | 1155.523951 | 3450.07581 | 154 (139) | 26.60958568 | 9.637643213 | 16.97194247 | 77 (118) |
| Finland | 0.859831368 | 995.2072219 | 253.586004 | 741.6212179 | 170 (186) | 5286.384855 | 2067.267573 | 3219.117282 | 30 (76) | 18.4211304 | 4.943575196 | 13.4775552 | 173 (186) |
| France | 0.838364875 | 1072.712539 | 331.0322655 | 741.680274 | 139 (172) | 5307.654176 | 2088.537158 | 3219.117018 | 27 (73) | 19.85734554 | 6.388146661 | 13.46919888 | 150 (176) |
| Gabon | 0.634691393 | 1344.2982 | 210.6367435 | 1133.661457 | 183 (119) | 5814.748405 | 2026.466031 | 3788.282374 | 38 (40) | 26.50496902 | 4.818590777 | 21.68637825 | 175 (121) |
| Gambia | 0.40971416 | 2094.948699 | 657.7606927 | 1437.188006 | 19 (13) | 5814.830107 | 1364.379677 | 4450.45043 | 129 (39) | 43.71987279 | 14.56182724 | 29.15804556 | 18 (13) |
| Georgia | 0.732473604 | 1380.870186 | 525.6116629 | 855.258523 | 50 (112) | 4376.000495 | 1156.882974 | 3219.117521 | 152 (181) | 27.70057883 | 11.83001418 | 15.87056465 | 46 (106) |
| Germany | 0.902957091 | 988.922514 | 247.1430233 | 741.7794908 | 172 (188) | 5308.881856 | 2089.764673 | 3219.117183 | 26 (72) | 18.22451867 | 4.763436692 | 13.46108198 | 178 (188) |
| Ghana | 0.56493039 | 1771.191835 | 560.733551 | 1210.458284 | 39 (39) | 5817.17528 | 2028.339544 | 3788.835736 | 35 (26) | 36.55339139 | 13.23525442 | 23.31813697 | 33 (37) |
| Greece | 0.791854408 | 1117.967729 | 357.1251549 | 760.8425739 | 127 (165) | 5298.960023 | 2079.843104 | 3219.116919 | 28 (74) | 21.00631439 | 7.153122203 | 13.85319219 | 136 (167) |
| Greenland | 0.826210336 | 852.3082217 | 110.8450002 | 741.4632215 | 201 (201) | 4375.201324 | 1156.0842 | 3219.117124 | 153 (182) | 15.88238266 | 2.396246799 | 13.48613586 | 202 (202) |
| Grenada | 0.668993028 | 1400.25296 | 500.2152672 | 900.0376928 | 59 (107) | 5170.193444 | 1703.979244 | 3466.214199 | 81 (122) | 27.45568396 | 9.725440901 | 17.73024306 | 76 (110) |
| Guam | 0.803982203 | 1033.13344 | 291.4277151 | 741.7057248 | 159 (182) | 4593.868283 | 1374.751178 | 3219.117105 | 120 (154) | 19.98538998 | 6.500525684 | 13.48486429 | 148 (174) |
| Guatemala | 0.539972424 | 1573.000525 | 344.9641757 | 1228.036349 | 133 (69) | 5180.985781 | 1390.816182 | 3790.169599 | 100 (89) | 31.00103517 | 7.026689415 | 23.97434576 | 139 (70) |
| Guinea | 0.336401293 | 2049.833887 | 528.2318825 | 1521.602005 | 49 (18) | 5817.142937 | 1366.690615 | 4450.452323 | 122 (27) | 42.73421702 | 12.89744918 | 29.83676784 | 35 (16) |
| Guinea-Bissau | 0.353109621 | 2150.86058 | 631.4507735 | 1519.409807 | 24 (11) | 5817.057109 | 1366.571684 | 4450.485425 | 123 (28) | 44.9131607 | 15.06437472 | 29.84878598 | 13 (9) |
| Guyana | 0.650812335 | 1497.624231 | 407.6931978 | 1089.931034 | 102 (82) | 5177.133988 | 1388.848485 | 3788.285503 | 106 (102) | 29.39570768 | 8.593036365 | 20.80267132 | 109 (87) |
| Haiti | 0.448278285 | 1982.730247 | 682.4122279 | 1300.318019 | 15 (22) | 5180.087533 | 729.6489737 | 4450.43856 | 183 (90) | 39.61377089 | 13.58288783 | 26.03088306 | 27 (25) |
| Honduras | 0.513037248 | 1694.041028 | 425.1322972 | 1268.908731 | 85 (50) | 5179.22462 | 1387.57926 | 3791.64536 | 109 (91) | 33.62354714 | 8.508018862 | 25.11552828 | 110 (51) |
| Hungary | 0.790754768 | 1179.01861 | 413.5430208 | 765.4755896 | 94 (154) | 3993.229 | 774.1114374 | 3219.117563 | 182 (201) | 23.50473573 | 9.523202599 | 13.98153314 | 85 (153) |
| Iceland | 0.87636168 | 1001.388301 | 259.9567616 | 741.4315393 | 164 (184) | 5284.509478 | 2065.391116 | 3219.118363 | 32 (78) | 18.48541042 | 4.99566251 | 13.48974791 | 171 (185) |
| India | 0.575401649 | 2026.898019 | 831.3705475 | 1195.527471 | 3 (19) | 6624.709991 | 2836.4505 | 3788.259491 | 5 (5) | 41.49242917 | 18.18256923 | 23.30985995 | 4 (20) |
| Indonesia | 0.656868336 | 1456.765024 | 359.5685739 | 1097.19645 | 125 (93) | 4821.525022 | 1033.228168 | 3788.296854 | 157 (134) | 29.38319022 | 8.611227454 | 20.77196277 | 108 (88) |
| Iran (Islamic Republic of) | 0.697207398 | 1554.334839 | 687.3091713 | 867.0256677 | 13 (71) | 5669.871418 | 2450.708048 | 3219.16337 | 10 (58) | 31.11446846 | 14.73342734 | 16.38104112 | 16 (69) |
| Iraq | 0.662626231 | 1427.596492 | 351.631387 | 1075.965105 | 129 (100) | 5175.407967 | 1398.540261 | 3776.867706 | 97 (115) | 28.54581261 | 7.847049037 | 20.69876357 | 120 (97) |
| Ireland | 0.87375385 | 953.9427307 | 212.1234212 | 741.8193096 | 182 (193) | 5325.8825 | 2106.765534 | 3219.116966 | 14 (60) | 17.48583476 | 4.016153071 | 13.46968169 | 186 (193) |
| Israel | 0.809011652 | 1071.868365 | 330.3199927 | 741.5483723 | 140 (173) | 5320.617786 | 2101.500811 | 3219.116975 | 17 (63) | 19.9809134 | 6.500537987 | 13.48037542 | 147 (175) |
| Italy | 0.805773534 | 1054.785588 | 313.588217 | 741.1973714 | 151 (177) | 4306.403354 | 1087.285534 | 3219.117821 | 156 (184) | 20.22048988 | 6.74520354 | 13.47528634 | 141 (172) |
| Jamaica | 0.683263064 | 1521.323784 | 654.7289484 | 866.594836 | 20 (78) | 5178.319585 | 1886.434405 | 3291.88518 | 73 (97) | 30.06391266 | 13.68295247 | 16.3809602 | 26 (80) |
| Japan | 0.871241813 | 1035.896385 | 294.0139874 | 741.8823978 | 158 (181) | 4399.708761 | 1180.591684 | 3219.117077 | 141 (167) | 19.77500922 | 6.299627088 | 13.47538213 | 155 (179) |
| Jordan | 0.725307227 | 1549.72785 | 682.7440492 | 866.9838011 | 14 (72) | 5171.760151 | 1952.636699 | 3219.123452 | 59 (121) | 31.32156746 | 14.94251833 | 16.37904913 | 14 (68) |
| Kazakhstan | 0.725144495 | 1204.065569 | 337.5439456 | 866.5216233 | 136 (149) | 4382.112058 | 1162.995106 | 3219.116952 | 144 (170) | 23.91963384 | 7.545957907 | 16.37367593 | 126 (146) |
| Kenya | 0.523768077 | 1734.221868 | 470.3300959 | 1263.891772 | 66 (47) | 6637.701974 | 2846.675725 | 3791.026249 | 4 (4) | 35.13362457 | 10.05906878 | 25.0745558 | 73 (44) |
| Kiribati | 0.527186583 | 1769.236161 | 515.3152249 | 1253.920936 | 53 (40) | 4609.042711 | 818.2733286 | 3790.769383 | 161 (138) | 35.49773271 | 11.05404306 | 24.44368964 | 57 (41) |
| Kuwait | 0.846651055 | 1162.545761 | 420.9919 | 741.5538615 | 89 (155) | 5166.633712 | 1947.516634 | 3219.117078 | 62 (124) | 22.83776558 | 9.365770576 | 13.47199501 | 88 (157) |
| Kyrgyzstan | 0.603979328 | 1620.301611 | 442.001712 | 1178.299899 | 79 (56) | 4380.232057 | 591.957413 | 3788.274644 | 192 (175) | 32.95529584 | 10.46397961 | 22.49131622 | 65 (54) |
| Lao People's Democratic Republic | 0.489136091 | 1453.485405 | 176.8552086 | 1276.630196 | 193 (95) | 4454.104252 | 265.7804456 | 4188.323806 | 196 (161) | 29.51957207 | 3.967151819 | 25.55242025 | 189 (85) |
| Latvia | 0.830663516 | 1273.787726 | 531.6895103 | 742.0982156 | 46 (132) | 4378.848579 | 1159.731423 | 3219.117156 | 149 (178) | 25.47369323 | 12.00842315 | 13.46527008 | 40 (131) |
| Lebanon | 0.744746351 | 1458.594997 | 612.0769303 | 846.5180662 | 29 (92) | 5169.78934 | 1950.67218 | 3219.11716 | 60 (123) | 29.26134387 | 13.56082175 | 15.70052212 | 29 (89) |
| Lesotho | 0.510393066 | 1853.01024 | 584.5126245 | 1268.497615 | 34 (33) | 5816.644197 | 2025.003482 | 3791.640715 | 41 (31) | 36.57401646 | 11.15278316 | 25.42123331 | 55 (36) |
| Liberia | 0.352442452 | 2349.970486 | 829.7750895 | 1520.195396 | 4 (3) | 5811.762627 | 1361.33371 | 4450.428917 | 133 (49) | 49.07497525 | 19.24725039 | 29.82772486 | 2 (3) |
| Libya | 0.725771399 | 1478.976238 | 612.6140745 | 866.3621631 | 28 (85) | 5177.490445 | 1958.373181 | 3219.117265 | 53 (101) | 29.74513488 | 13.38605416 | 16.35908072 | 30 (84) |
| Lithuania | 0.856484049 | 1245.88892 | 504.6470659 | 741.2418545 | 56 (140) | 4381.842969 | 1162.726052 | 3219.116917 | 145 (171) | 24.83385897 | 11.36521254 | 13.46864643 | 53 (136) |
| Luxembourg | 0.884428955 | 905.1395116 | 163.9693798 | 741.1701318 | 195 (198) | 5298.683996 | 2079.567065 | 3219.116931 | 29 (75) | 16.36426965 | 2.893289565 | 13.47098008 | 199 (200) |
| Madagascar | 0.400246943 | 1905.11548 | 446.1014578 | 1459.014023 | 77 (29) | 6123.996272 | 1673.561459 | 4450.434813 | 88 (21) | 38.82308241 | 9.421193094 | 29.40188931 | 86 (28) |
| Malawi | 0.384553634 | 2017.32233 | 508.3922772 | 1508.930053 | 55 (20) | 6382.922811 | 1932.484288 | 4450.438522 | 67 (6) | 40.10500591 | 10.26466321 | 29.84034269 | 67 (23) |
| Malaysia | 0.742523828 | 1153.836844 | 304.7149874 | 849.1218564 | 153 (158) | 4450.041598 | 1230.924298 | 3219.1173 | 138 (164) | 22.96372257 | 7.220923877 | 15.74279869 | 135 (155) |
| Maldives | 0.650886627 | 1256.227339 | 173.5137266 | 1082.713613 | 194 (137) | 4437.799936 | 649.5343931 | 3788.265543 | 188 (166) | 25.17832691 | 4.348362853 | 20.82996406 | 184 (134) |
| Mali | 0.268579941 | 2060.891089 | 540.784156 | 1520.106933 | 43 (16) | 5814.098052 | 1225.628664 | 4588.469388 | 140 (44) | 42.91471733 | 13.07554542 | 29.83917191 | 34 (15) |
| Malta | 0.801585034 | 1041.606731 | 297.6946025 | 743.9121282 | 157 (180) | 5285.123658 | 2066.006187 | 3219.117471 | 31 (77) | 19.34837076 | 5.837223353 | 13.51114741 | 162 (182) |
| Marshall Islands | 0.574091128 | 1609.383602 | 413.0988756 | 1196.284726 | 95 (59) | 4604.855753 | 816.540352 | 3788.315401 | 163 (141) | 32.065656 | 8.78675236 | 23.27890364 | 102 (61) |
| Mauritania | 0.4989451 | 1865.047577 | 592.2119134 | 1272.835664 | 33 (31) | 5815.541919 | 2022.800319 | 3792.7416 | 42 (36) | 38.72515279 | 13.29741375 | 25.42773904 | 31 (29) |
| Mauritius | 0.718260446 | 1194.447605 | 327.4147375 | 867.0328675 | 144 (151) | 4454.221551 | 1235.090719 | 3219.130832 | 137 (160) | 23.79835677 | 7.42339939 | 16.37495738 | 130 (150) |
| Mexico | 0.664575304 | 1471.128582 | 397.4416526 | 1073.68693 | 107 (87) | 5680.67312 | 1905.043218 | 3775.629902 | 71 (56) | 28.9162001 | 8.216299726 | 20.69990038 | 117 (95) |
| Micronesia (Federated States of) | 0.587534967 | 1629.959432 | 437.1208557 | 1192.838577 | 80 (55) | 4604.851337 | 816.6033397 | 3788.247997 | 162 (142) | 32.60948721 | 9.551174627 | 23.05831258 | 82 (57) |
| Monaco | 0.908262831 | 757.6985732 | 16.25501665 | 741.4435565 | 204 (204) | 5319.568349 | 2100.450127 | 3219.118222 | 18 (64) | 13.4267737 | 0.008110397 | 13.4186633 | 204 (204) |
| Mongolia | 0.617621565 | 1349.053134 | 183.9132366 | 1165.139897 | 189 (115) | 4383.650794 | 595.3405939 | 3788.3102 | 191 (169) | 27.05238 | 4.84312451 | 22.20925549 | 174 (114) |
| Montenegro | 0.795800584 | 1260.764597 | 511.7802146 | 748.9843825 | 54 (136) | 4008.408689 | 789.2904388 | 3219.11825 | 170 (189) | 25.25994049 | 11.57795918 | 13.68198131 | 49 (132) |
| Morocco | 0.562698301 | 1586.07714 | 380.1249969 | 1205.952143 | 116 (66) | 5178.890606 | 1389.930704 | 3788.959902 | 104 (94) | 32.08743305 | 8.722966931 | 23.36446612 | 105 (60) |
| Mozambique | 0.326462614 | 1934.128802 | 412.9507134 | 1521.178089 | 96 (26) | 6125.02214 | 1674.434354 | 4450.587786 | 86 (19) | 38.70150443 | 8.847892722 | 29.85361171 | 99 (30) |
| Myanmar | 0.53390084 | 1486.396606 | 236.9256196 | 1249.470987 | 176 (83) | 4457.185277 | 666.860122 | 3790.325155 | 186 (156) | 30.34587881 | 5.85842348 | 24.48745533 | 161 (76) |
| Namibia | 0.617564872 | 1527.415189 | 361.7928867 | 1165.622303 | 123 (75) | 5815.998513 | 2027.685032 | 3788.313481 | 36 (34) | 29.85532514 | 7.665797087 | 22.18952806 | 123 (83) |
| Nauru | 0.625177834 | 1310.324068 | 160.0111158 | 1150.312952 | 197 (125) | 4604.144507 | 815.8417821 | 3788.302725 | 164 (143) | 25.78506647 | 3.834707367 | 21.9503591 | 194 (127) |
| Nepal | 0.433174635 | 2291.206286 | 929.4591469 | 1361.747139 | 1 (4) | 6234.131705 | 1783.688955 | 4450.44275 | 75 (9) | 46.73743868 | 19.90629764 | 26.83114103 | 1 (5) |
| Netherlands | 0.888464256 | 990.7636652 | 249.4617001 | 741.3019651 | 171 (187) | 5321.17669 | 2102.059827 | 3219.116863 | 16 (62) | 18.21788323 | 4.714318342 | 13.50356489 | 179 (189) |
| New Zealand | 0.849442499 | 898.3397885 | 157.0900375 | 741.249751 | 198 (199) | 5409.008435 | 2189.891188 | 3219.117248 | 12 (59) | 16.61282201 | 3.132035557 | 13.48078645 | 197 (198) |
| Nicaragua | 0.523958472 | 1680.031476 | 415.4739815 | 1264.557495 | 92 (52) | 5176.102062 | 1384.882837 | 3791.219225 | 114 (111) | 33.3130322 | 8.355250767 | 24.95778143 | 114 (53) |
| Niger | 0.168072774 | 2381.989139 | 163.0816658 | 2218.907474 | 196 (2) | 5815.462551 | 1.492253257 | 5813.970298 | 202 (37) | 50.17407496 | 4.374510915 | 45.79956404 | 183 (2) |
| Nigeria | 0.503390833 | 1800.743401 | 531.0057808 | 1269.73762 | 47 (36) | 6342.178464 | 2549.89154 | 3792.286924 | 7 (7) | 37.09984678 | 11.6753581 | 25.42448868 | 48 (34) |
| Niue | 0.72622205 | 1296.893692 | 436.4802238 | 860.4134683 | 81 (127) | 4601.968024 | 1382.849174 | 3219.11885 | 117 (147) | 25.56859477 | 9.588810639 | 15.97978413 | 79 (130) |
| North Macedonia | 0.750629703 | 1309.561159 | 464.3351196 | 845.226039 | 70 (126) | 3998.865135 | 779.7462432 | 3219.118892 | 179 (198) | 26.29206689 | 10.64906128 | 15.64300561 | 61 (124) |
| Northern Mariana Islands | 0.771535213 | 1109.701959 | 299.3642181 | 810.3377411 | 155 (166) | 4594.026322 | 1374.909307 | 3219.117015 | 119 (153) | 21.61044881 | 6.700360357 | 14.91008845 | 143 (163) |
| Norway | 0.91613281 | 1070.050657 | 328.399835 | 741.6508221 | 143 (174) | 5790.99788 | 2571.880108 | 3219.117772 | 6 (53) | 19.79124977 | 6.368157129 | 13.42309264 | 151 (178) |
| Oman | 0.773391602 | 1193.481834 | 381.4964114 | 811.9854223 | 114 (153) | 5164.076909 | 1944.960001 | 3219.116907 | 63 (125) | 23.7252217 | 8.792533515 | 14.93268819 | 101 (151) |
| Pakistan | 0.504028689 | 2087.684941 | 816.1684987 | 1271.516442 | 5 (14) | 6767.997729 | 2975.747101 | 3792.250628 | 3 (1) | 41.95865202 | 16.55205531 | 25.40659671 | 8 (18) |
| Palau | 0.754046931 | 1253.01009 | 412.0646664 | 840.9454235 | 98 (138) | 4592.812059 | 1373.694031 | 3219.118028 | 121 (155) | 24.59688545 | 9.011364603 | 15.58552084 | 92 (142) |
| Palestine | 0.631011665 | 1759.282735 | 618.2257989 | 1141.056936 | 27 (42) | 5178.207889 | 1389.940256 | 3788.267633 | 103 (98) | 35.84282601 | 14.10565901 | 21.737167 | 21 (39) |
| Panama | 0.708864828 | 1331.428849 | 463.7475689 | 867.6812805 | 71 (122) | 5172.842767 | 1953.713364 | 3219.129403 | 57 (119) | 25.93438202 | 9.566425805 | 16.36795622 | 80 (126) |
| Papua New Guinea | 0.417797443 | 1586.135195 | 152.0803884 | 1434.054807 | 199 (65) | 4600.04875 | 149.5995123 | 4450.449237 | 199 (150) | 31.63061732 | 2.466749832 | 29.16386749 | 201 (66) |
| Paraguay | 0.635718099 | 1390.185226 | 255.2174259 | 1134.9678 | 168 (109) | 5177.717216 | 1389.390314 | 3788.326902 | 105 (100) | 27.55906126 | 5.914531397 | 21.64452987 | 160 (109) |
| Peru | 0.662054037 | 1385.769344 | 317.4123688 | 1068.356976 | 147 (110) | 5176.304926 | 1399.297428 | 3777.007498 | 96 (106) | 26.52087776 | 5.775944455 | 20.7449333 | 163 (119) |
| Philippines | 0.651219329 | 1522.466241 | 432.4368347 | 1090.029406 | 83 (77) | 4820.528389 | 1032.186282 | 3788.342107 | 158 (135) | 30.98951612 | 10.17412127 | 20.81539485 | 69 (71) |
| Poland | 0.812042809 | 1219.331636 | 478.1153914 | 741.2162447 | 62 (146) | 3767.258428 | 548.1415435 | 3219.116885 | 194 (203) | 24.66133687 | 11.13779727 | 13.5235396 | 56 (140) |
| Portugal | 0.744151851 | 1053.036694 | 206.1557603 | 846.8809335 | 184 (179) | 5319.369154 | 2100.25196 | 3219.117194 | 19 (65) | 19.59089251 | 3.872744653 | 15.71814786 | 191 (181) |
| Puerto Rico | 0.825525847 | 1212.387628 | 471.0714591 | 741.3161688 | 65 (147) | 5185.722072 | 1966.605005 | 3219.117067 | 47 (81) | 23.46642441 | 9.989357891 | 13.47706652 | 74 (154) |
| Qatar | 0.846860584 | 1101.363637 | 359.6753279 | 741.6883086 | 124 (168) | 5140.428543 | 1921.311626 | 3219.116917 | 69 (129) | 21.55964725 | 8.079861356 | 13.4797859 | 119 (165) |
| Republic of Korea | 0.886675267 | 958.0874141 | 216.4888079 | 741.5986062 | 179 (191) | 4013.06791 | 793.9487469 | 3219.119163 | 168 (187) | 18.28037577 | 4.791420418 | 13.48895535 | 176 (187) |
| Republic of Moldova | 0.732214875 | 1599.922222 | 744.9025463 | 855.0196761 | 8 (63) | 4381.340532 | 1162.223333 | 3219.117199 | 146 (173) | 32.55543356 | 16.63252742 | 15.92290614 | 6 (58) |
| Romania | 0.768453864 | 1194.440391 | 380.2138304 | 814.2265603 | 115 (152) | 4003.847727 | 784.7307687 | 3219.116958 | 175 (194) | 23.82888167 | 8.855444274 | 14.97343739 | 98 (149) |
| Russian Federation | 0.808536005 | 1373.884611 | 632.0413825 | 741.8432284 | 23 (113) | 4765.6672 | 1546.549479 | 3219.11772 | 93 (137) | 27.60185145 | 14.11997948 | 13.48187197 | 20 (107) |
| Rwanda | 0.435588706 | 1753.002147 | 405.8752545 | 1347.126893 | 103 (45) | 6127.450334 | 1676.996478 | 4450.453856 | 85 (17) | 34.97363412 | 8.319192084 | 26.65444203 | 115 (46) |
| Saint Kitts and Nevis | 0.754987055 | 1262.400184 | 422.3228914 | 840.0772926 | 87 (135) | 5175.07372 | 1955.956501 | 3219.117219 | 56 (116) | 24.50281362 | 8.950458115 | 15.55235551 | 93 (143) |
| Saint Lucia | 0.672509735 | 1424.429242 | 519.1550471 | 905.2741949 | 52 (102) | 5177.821407 | 1724.69686 | 3453.124547 | 80 (99) | 27.92799905 | 10.94138194 | 16.98661712 | 58 (104) |
| Saint Vincent and the Grenadines | 0.637195963 | 1455.453491 | 317.1602275 | 1138.293263 | 148 (94) | 5172.965143 | 1384.667646 | 3788.297497 | 115 (118) | 28.60904451 | 7.002116729 | 21.60692778 | 140 (96) |
| Samoa | 0.593392769 | 1470.842149 | 281.9395884 | 1188.90256 | 163 (89) | 4600.666098 | 812.3363573 | 3788.329741 | 166 (149) | 29.24635059 | 6.353735033 | 22.89261555 | 152 (91) |
| San Marino | 0.888005474 | 978.3619205 | 236.9746561 | 741.3872643 | 175 (189) | 5323.535831 | 2104.418312 | 3219.117518 | 15 (61) | 17.97084581 | 4.465301559 | 13.50554425 | 181 (191) |
| Sao Tome and Principe | 0.505413747 | 1899.31624 | 630.0589207 | 1269.25732 | 25 (30) | 5807.6337 | 2015.382109 | 3792.251591 | 45 (51) | 39.51063496 | 14.08097857 | 25.42965639 | 22 (26) |
| Saudi Arabia | 0.815143493 | 1209.725606 | 468.2106159 | 741.51499 | 68 (148) | 5162.133322 | 1943.01649 | 3219.116833 | 64 (126) | 23.87095535 | 10.39270475 | 13.4782506 | 66 (148) |
| Senegal | 0.408054193 | 1917.026779 | 469.6875481 | 1447.33923 | 67 (28) | 5814.482362 | 1363.978028 | 4450.504334 | 130 (42) | 39.75432465 | 10.53538533 | 29.21893932 | 64 (24) |
| Serbia | 0.792416294 | 1284.78298 | 523.5547441 | 761.2282356 | 51 (131) | 3999.372769 | 780.2556776 | 3219.117091 | 178 (197) | 25.7504296 | 11.79388015 | 13.95654944 | 47 (129) |
| Seychelles | 0.730150775 | 1152.541777 | 298.0586742 | 854.4831026 | 156 (159) | 4449.525554 | 1230.408098 | 3219.117456 | 139 (165) | 22.93313379 | 7.073671075 | 15.85946272 | 138 (156) |
| Sierra Leone | 0.358665881 | 2217.018588 | 697.1627942 | 1519.855794 | 12 (7) | 5812.753079 | 1362.315066 | 4450.438013 | 132 (48) | 46.33485151 | 16.48045561 | 29.85439591 | 9 (6) |
| Singapore | 0.856097766 | 851.6974684 | 109.979315 | 741.7181534 | 202 (202) | 4011.360286 | 792.2424403 | 3219.117846 | 169 (188) | 16.07632192 | 2.593002618 | 13.4833193 | 200 (201) |
| Slovakia | 0.81061053 | 1146.468399 | 404.8242201 | 741.6441793 | 104 (160) | 4006.947814 | 787.8291521 | 3219.118662 | 172 (191) | 22.78396028 | 9.315299091 | 13.46866119 | 89 (158) |
| Slovenia | 0.842430731 | 1130.517068 | 389.1772769 | 741.3397911 | 111 (162) | 3997.887522 | 778.7704833 | 3219.117039 | 180 (199) | 22.29433316 | 8.817482314 | 13.47685085 | 100 (162) |
| Solomon Islands | 0.429360316 | 1748.804955 | 410.0135842 | 1338.79137 | 100 (46) | 4602.800323 | 152.3578863 | 4450.442436 | 198 (144) | 35.14410194 | 7.747417349 | 27.39668459 | 122 (43) |
| Somalia | 0.077688109 | 2925.496933 | 282.5761293 | 2642.920804 | 161 (1) | 6134.461033 | 0.090926748 | 6134.370107 | 203 (14) | 60.21279745 | 5.998931049 | 54.2138664 | 159 (1) |
| South Africa | 0.679626598 | 1613.3184 | 745.728807 | 867.5895934 | 7 (57) | 6335.656509 | 3060.813779 | 3274.842731 | 2 (8) | 31.6254013 | 15.23948806 | 16.38591324 | 12 (67) |
| South Sudan | 0.278371125 | 1763.298891 | 242.5050527 | 1520.793838 | 174 (41) | 6135.142664 | 1684.093518 | 4451.049145 | 82 (13) | 35.0855916 | 5.267510247 | 29.81808136 | 168 (45) |
| Spain | 0.769283698 | 1132.599855 | 316.2766034 | 816.3232512 | 150 (161) | 6702.592671 | 3483.475463 | 3219.117207 | 1 (2) | 20.78607754 | 5.725200918 | 15.06087662 | 164 (169) |
| Sri Lanka | 0.701534935 | 1315.558976 | 447.7655338 | 867.7934418 | 75 (124) | 4456.872316 | 1235.879098 | 3220.993219 | 136 (157) | 26.46630602 | 10.088526 | 16.37778002 | 72 (122) |
| Sudan | 0.541949735 | 1788.52496 | 564.9794526 | 1223.545508 | 36 (37) | 5176.261471 | 1386.418734 | 3789.842737 | 113 (108) | 36.51161125 | 12.50670624 | 24.00490501 | 38 (38) |
| Suriname | 0.633665739 | 1393.462759 | 257.5031931 | 1135.959566 | 165 (108) | 5175.729682 | 1387.43488 | 3788.294802 | 110 (112) | 27.26593663 | 5.5907128 | 21.67522383 | 166 (111) |
| Sweden | 0.886880299 | 1077.925309 | 336.5861942 | 741.3391143 | 137 (171) | 5757.842627 | 2538.725047 | 3219.11758 | 8 (54) | 19.80353819 | 6.317205658 | 13.48633253 | 154 (177) |
| Switzerland | 0.933059111 | 956.7985134 | 215.2992801 | 741.4992333 | 180 (192) | 5311.002733 | 2091.885001 | 3219.117732 | 25 (71) | 17.40814055 | 3.982221009 | 13.42591954 | 188 (194) |
| Syrian Arab Republic | 0.623004075 | 1756.124948 | 598.0893502 | 1158.035598 | 32 (44) | 5185.865882 | 1397.558386 | 3788.307496 | 98 (80) | 35.73336876 | 13.73728799 | 21.99608077 | 25 (40) |
| Taiwan (Province of China) | 0.874747053 | 969.3258058 | 227.828254 | 741.4975518 | 177 (190) | 3220.686614 | 1.569389925 | 3219.117224 | 201 (204) | 18.61339867 | 5.155192714 | 13.45820596 | 169 (184) |
| Tajikistan | 0.541511187 | 1700.450744 | 476.5708633 | 1223.879881 | 63 (49) | 4378.736493 | 588.9304158 | 3789.806077 | 193 (179) | 34.66329908 | 10.80097438 | 23.8623247 | 59 (47) |
| Thailand | 0.682547933 | 1290.723872 | 423.8189474 | 866.9049245 | 86 (129) | 4454.807197 | 1168.389502 | 3286.417696 | 142 (159) | 25.93553694 | 9.563836795 | 16.37170015 | 81 (125) |
| Timor-Leste | 0.444667619 | 1481.645934 | 178.3324971 | 1303.313437 | 191 (84) | 4452.346092 | 1.912767407 | 4450.433325 | 200 (162) | 30.10912343 | 3.858179677 | 26.25094376 | 193 (78) |
| Togo | 0.408533695 | 2166.250743 | 721.9740168 | 1444.276726 | 11 (8) | 5816.868389 | 1366.384329 | 4450.48406 | 124 (29) | 45.32051739 | 16.04743366 | 29.27308373 | 10 (8) |
| Tokelau | 0.686425621 | 1421.799984 | 554.7461767 | 867.0538072 | 40 (103) | 4600.964073 | 1344.244539 | 3256.719533 | 135 (148) | 28.20354859 | 11.84412236 | 16.35942623 | 45 (102) |
| Tonga | 0.626349936 | 1470.9886 | 317.9559758 | 1153.032624 | 146 (88) | 4602.46441 | 814.1656537 | 3788.298756 | 165 (145) | 29.2515017 | 7.275183066 | 21.97631863 | 133 (90) |
| Trinidad and Tobago | 0.768763254 | 1224.599214 | 408.6734657 | 815.9257479 | 101 (145) | 5176.300432 | 1957.182939 | 3219.117493 | 55 (107) | 23.70785488 | 8.726118643 | 14.98173623 | 104 (152) |
| Tunisia | 0.682432216 | 1500.327099 | 633.7592078 | 866.5678915 | 22 (81) | 5176.167619 | 1915.593238 | 3260.574381 | 70 (109) | 30.21050176 | 13.82052465 | 16.38997711 | 24 (77) |
| Turkmenistan | 0.682160776 | 1242.967282 | 356.6618364 | 886.3054453 | 128 (142) | 4372.830865 | 1103.584853 | 3269.246012 | 155 (183) | 24.78266734 | 8.409815664 | 16.37285167 | 112 (138) |
| Tuvalu | 0.576620529 | 1596.251095 | 399.8525575 | 1196.398537 | 105 (64) | 4598.448032 | 810.1604401 | 3788.287592 | 167 (152) | 31.92410578 | 8.65611165 | 23.26799413 | 107 (63) |
| Türkiye | 0.712692673 | 1322.887102 | 456.0373544 | 866.8497472 | 72 (123) | 5178.350621 | 1959.226161 | 3219.12446 | 52 (96) | 26.3109293 | 9.921801165 | 16.38912813 | 75 (123) |
| Uganda | 0.423261181 | 1757.560002 | 330.3197504 | 1427.240252 | 141 (43) | 6131.83794 | 1681.408089 | 4450.42985 | 84 (16) | 34.62927859 | 5.641284984 | 28.98799361 | 165 (49) |
| Ukraine | 0.760773913 | 1573.159639 | 739.1915761 | 833.9680634 | 9 (68) | 4774.912668 | 1555.795426 | 3219.117242 | 92 (136) | 31.95317435 | 16.57524778 | 15.37792657 | 7 (62) |
| United Arab Emirates | 0.849317734 | 1155.895715 | 414.0605005 | 741.8352146 | 93 (157) | 5148.678932 | 1929.561831 | 3219.117101 | 68 (128) | 22.72437904 | 9.217732942 | 13.5066461 | 90 (159) |
| United Kingdom | 0.859000182 | 1089.89706 | 348.4684842 | 741.4285761 | 132 (170) | 5721.194672 | 2502.077327 | 3219.117345 | 9 (55) | 20.22525521 | 6.72873765 | 13.49651756 | 142 (171) |
| United Republic of Tanzania | 0.446568273 | 1671.96305 | 365.3873139 | 1306.575736 | 121 (54) | 6124.742144 | 1674.319628 | 4450.422516 | 87 (20) | 32.79105803 | 6.672318385 | 26.11873964 | 145 (56) |
| United States Virgin Islands | 0.821830853 | 1161.139541 | 419.2090838 | 741.9304576 | 90 (156) | 5183.574209 | 1964.457147 | 3219.117062 | 51 (85) | 22.384674 | 8.915770998 | 13.46890301 | 96 (161) |
| United States of America | 0.862448354 | 934.8023048 | 193.2802416 | 741.5220631 | 188 (196) | 4168.218722 | 949.0992058 | 3219.119516 | 160 (186) | 17.75223401 | 4.268158787 | 13.48407522 | 185 (192) |
| Uruguay | 0.719283445 | 1067.26639 | 200.2459603 | 867.0204297 | 185 (175) | 4956.187595 | 1737.06528 | 3219.122315 | 78 (132) | 20.28484478 | 3.912544794 | 16.37229999 | 190 (170) |
| Uzbekistan | 0.662621694 | 1437.643382 | 366.977491 | 1070.665891 | 120 (99) | 4381.583119 | 606.9910326 | 3774.592087 | 190 (172) | 28.93299976 | 8.233024699 | 20.69997506 | 116 (92) |
| Vanuatu | 0.473100706 | 1672.21293 | 389.9393138 | 1282.273616 | 110 (53) | 4605.380449 | 404.0090618 | 4201.371387 | 195 (140) | 33.49313216 | 7.80787138 | 25.68526078 | 121 (52) |
| Venezuela (Bolivarian Republic of) | 0.596513059 | 1471.931237 | 282.7416954 | 1189.189541 | 160 (86) | 5176.920656 | 1388.588436 | 3788.33222 | 107 (104) | 28.91950491 | 6.033385613 | 22.8861193 | 158 (94) |
| Viet Nam | 0.627933721 | 1406.067552 | 256.0498744 | 1150.017678 | 166 (106) | 4451.643117 | 663.3361912 | 3788.306926 | 187 (163) | 28.52621592 | 6.551522406 | 21.97469351 | 146 (98) |
| Yemen | 0.450376375 | 1977.94503 | 668.0772228 | 1309.867807 | 16 (23) | 5179.030269 | 728.5969786 | 4450.433291 | 184 (93) | 40.46027145 | 14.33524028 | 26.12503118 | 19 (21) |
| Zambia | 0.505948954 | 1605.232658 | 335.7269586 | 1269.505699 | 138 (61) | 6121.823471 | 2329.593296 | 3792.230175 | 11 (22) | 31.69312404 | 6.274228273 | 25.41889577 | 156 (65) |
| Zimbabwe | 0.473819486 | 1945.911475 | 662.2445897 | 1283.666885 | 18 (24) | 5817.602218 | 1597.652557 | 4219.949661 | 91 (25) | 38.43708128 | 12.82561582 | 25.61146547 | 36 (32) |
